# A transcriptional MAPK Pathway Activity Score (MPAS) is a clinically relevant biomarker in multiple cancer types

**DOI:** 10.1038/s41698-018-0051-4

**Published:** 2018-03-07

**Authors:** Marie-Claire Wagle, Daniel Kirouac, Christiaan Klijn, Bonnie Liu, Shilpi Mahajan, Melissa Junttila, John Moffat, Mark Merchant, Ling Huw, Matthew Wongchenko, Kwame Okrah, Shrividhya Srinivasan, Zineb Mounir, Teiko Sumiyoshi, Peter M. Haverty, Robert L. Yauch, Yibing Yan, Omar Kabbarah, Garret Hampton, Lukas Amler, Saroja Ramanujan, Mark R. Lackner, Shih-Min A. Huang

**Affiliations:** 1 Department of Oncology Biomarker Development, Genentech, 1 DNA Way, South San Francisco, CA 94080 USA; 2 Department of Pre-Clinical and Translational PKPD, Genentech, 1 DNA Way, South San Francisco, CA 94080 USA; 3 Department of Bioinformatics, Genentech, 1 DNA Way, South San Francisco, CA 94080 USA; 4 Department of Translational Oncology, Genentech, 1 DNA Way, South San Francisco, CA 94080 USA; 5 Department of Biochemical and Cellular pharmacology, Genentech, 1 DNA Way, South San Francisco, CA 94080 USA; 6 Department of Biostatistics, Genentech, 1 DNA Way, South San Francisco, CA 94080 USA; 7 Present Address: Bristol-Myers Squibb, 3551 Lawrenceville Princeton, Lawrence Township, NJ 08648 USA

## Abstract

*KRAS*- and *BRAF*-mutant tumors are often dependent on MAPK signaling for proliferation and survival and thus sensitive to MAPK pathway inhibitors. However, clinical studies have shown that MEK inhibitors are not uniformly effective in these cancers indicating that mutational status of these oncogenes does not accurately capture MAPK pathway activity. A number of transcripts are regulated by this pathway and are recurrently identified in genome-based MAPK transcriptional signatures. To test whether the transcriptional output of only 10 of these targets could quantify MAPK pathway activity with potential predictive or prognostic clinical utility, we created a MAPK Pathway Activity Score (MPAS) derived from aggregated gene expression. In vitro, MPAS predicted sensitivity to MAPK inhibitors in multiple cell lines, comparable to or better than larger genome-based statistical models. Bridging in vitro studies and clinical samples, median MPAS from a given tumor type correlated with cobimetinib (MEK inhibitor) sensitivity of cancer cell lines originating from the same tissue type. Retrospective analyses of clinical datasets showed that MPAS was associated with the sensitivity of melanomas to vemurafenib (HR: 0.596) and negatively prognostic of overall or progression-free survival in both adjuvant and metastatic CRC (HR: 1.5 and 1.4), adrenal cancer (HR: 1.7), and HER2+ breast cancer (HR: 1.6). MPAS thus demonstrates potential clinical utility that warrants further exploration.

## Biomarker: Gene signature predicts drug responses and patient outcomes

A clinical score based on the activity of genes that regulate cell signaling can predict drug sensitivity and patient outcomes across a range of cancer types. Marie-Claire Wagle, Daniel Kirouac and their colleagues at Genentech in South San Francisco, California, USA developed an index that aggregates expression levels of 10 genes involved in modulating the mitogen-activated protein kinase (MAPK) pathway. This “MAPK Pathway Activity Score”, or MPAS, performed as well or better than other more complicated, genome-based tools at predicting whether drugs that inhibit MAPK-related enzymes were active against tumor cell lines. Retrospective analyses of clinical datasets also showed that MPAS correlated with survival outcomes in patients with melanoma, colon cancer, and breast cancer. The authors suggest that MPAS should be evaluated more broadly and perhaps implemented as a clinically informative biomarker.

## Introduction

The mitogen-activated protein kinase (MAPK) pathway is a conserved developmental pathway that regulates organ development and tissue homeostasis by transmitting signals through a series of phosphorylation events emanating from receptor tyrosine kinases (RTKs), RAS family members, RAF family members, mitogen-activated extracellular signal-regulated kinase 1/2 (MEK1/2) (*MAP2K1/2*), and extracellular signal-regulated kinase 1/2 (ERK1/2) (*MAPK3/1*). Nuclear translocation of activated ERK1/2 then triggers the transcriptional activation of multiple target genes involved in modulating the cellular processes of differentiation, proliferation, survival, migration, and angiogenesis.^1^ Enhanced MAPK pathway activity can be driven by genetic alterations (including amplification and genetic mutations) in RTKs and RAS/RAF family members, as well as aberrant growth factor signaling.^1,2^ The high prevalence of aberrant MAPK signaling in cancer has motivated the clinical development of inhibitors targeting critical pathway nodes, including *BRAF*^V600^-mutant selective inhibitors, pan-RAF inhibitors, MEK1/2 inhibitors, and ERK1/2 inhibitors.^3–6^ Currently, there are at least 20 different MEK, RAF, and ERK inhibitors being tested alone or in combination with other drugs in active clinical trials according to www.clinicaltrials.gov. While single genetic events may not always correspond to pathway hyperactivation in tumors,^7^ in some instances, these drugs have demonstrated therapeutic benefit alone or in combination in pre-selected patient populations defined by genetic mutations. For example, treatment of *BRAF*^V600E^ or *BRAF*^V600K^ mutant melanoma patients with *BRAF* inhibitors (vemurafenib or dabrafenib) increases overall survival,^4,8^ and the combination with MEK1/2 inhibitors (cobimetinib or trametinib) achieves deeper MAPK pathway inhibition, resulting in improved clinical efficacy.^9–11^ However, clinical responses to MAPK pathway inhibition in mutant *BRAF* populations outside of melanoma are more variable,^12,13^ and the therapeutic benefit of MEK1/2 inhibitors trametinib, selumetinib, and refametinib does not appear to be associated with *KRAS* mutation status in lung cancer or pancreatic cancer.^14–16^ ERK phosphorylation also varies extensively across cell lines and does not predict MEK inhibitor sensitivity.^17^ Together, these observations suggest that neither *BRAF/ KRAS* mutation status nor ERK phosphorylation status alone are sufficiently reliable biomarkers of MAPK pathway activity in multiple cancer indications and that additional, clinically translatable biomarkers are needed.

Developmental pathways such as MAPK often regulate the activity of a conserved set of transcriptional targets across multiple tissues.^18^ For example, WNT pathway activity is reflected in the levels of downstream transcriptional targets such as *AXIN2*, *NKD1*, *RNF43*, *ZNRF3*, etc.^19,20^ Likewise, transcript levels of *GLI1* and *GLI2* are also representative of Sonic Hedgehog (SHH) pathway activity and *GLI1 t*ranscript levels have been measured in SHH inhibitor clinical trials as a pharmacodynamic biomarker to assess on-target activity.^21^ For the MAPK pathway, while multiple predictive transcriptional gene signatures have been identified from in vitro data, clinically translating these large gene sets to multivariate biomarkers is challenging due to many technical and practical issues. However, genes that are common between these signatures include direct targets of the MAPK pathway.^17,22–24^ We hypothesized that a simple aggregated gene signature consisting of direct, biologically conserved transcriptional targets of MAPK/ERK signaling may sufficiently capture the activation status of the pathway across different tumor types.

Here we show that a MAPK Pathway Activity Score (MPAS), derived from the transcript levels of 10 MAPK target genes, correlates exclusively with MAPK inhibitor sensitivity in multiple cell lines and primary tumor samples. Retrospective analysis of CoBRIM clinical trial data shows that high MPAS correlates with improved progression-free survival (PFS) of vemurafenib-treated patients in *BRAF*^V600E^ melanoma.^9^ Furthermore, we demonstrate that MPAS is associated with poor prognosis in adjuvant and metastatic colorectal cancer (CRC), adrenal cancers, and HER2-positive breast cancer. These findings illustrate that MPAS could potentially serve as a predictive and/or prognostic biomarker, thus paving the way for further exploration of the clinical utility of MPAS in the near future.

## Results

### MPAS correlates with and predicts the sensitivity of cell lines to MEK1/2 inhibitors

To assess whether a small, hypothesis-driven gene set could represent MAPK pathway activity, we identified 10 genes (SPRY2, SPRY4, ETV4, ETV5, DUSP4, DUSP6, CCND1, EPHA2, and EPHA4) that have been reported in multiple gene signatures predictive of sensitivity to MAPK inhibition.^17,22–24^ Drugs used in these studies include the MEK inhibitors selumetinib, PD0325901, CI-1040, and RDEA-119 and the BRAF inhibitors, vemurafenib, dabrafenib, and AZ628. These genes also correlate with the extent of tumor response to cobimetinib and the ERK inhibitor, GDC-0994, observed in both pancreatic ductal adenocarcinoma and non-small cell lung cancer (NSCLC) genetically engineered mouse models.^25^ Furthermore, these genes were the most consistently and dose-dependently inhibited by cobimetinib in three cobimetinib-sensitive cell lines: MEL-JUSO (melanoma), NCI-H2347 (NSCLC), and HUP-T4 (pancreatic cancer), compared to other MAPK pathway targets (INPP5F, MAP2K3, TRIB2, and ETV1) that have also been identified as responsive genes in the literature (Supplementary Table 1).^17,22–24^ To determine how expression patterns of these genes vary across different tissue types, we measured the expression of each gene in tumor specimens from CRC, NSCLC, and melanoma patients (Supplementary Figure 1a). All 10 MAPK genes were robustly expressed across these tissue types, and the expression of each gene correlated well with the majority of other MPAS genes in at least one of the indications tested (Supplementary Figure 1b).

**Fig. 1:**
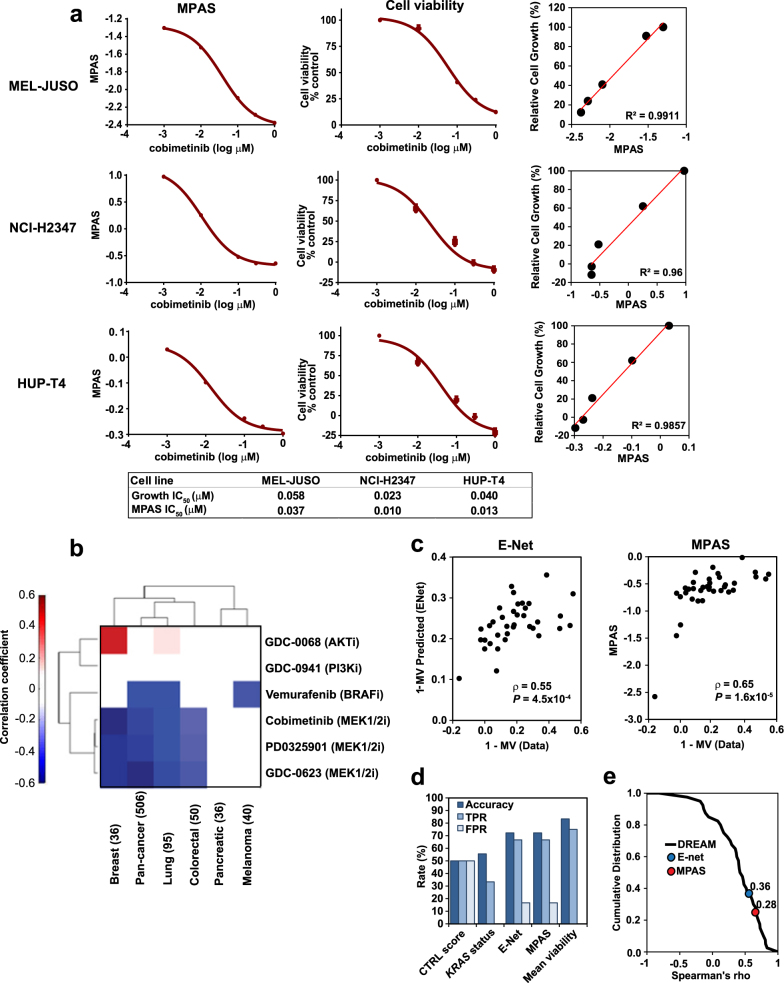
Aggregated gene expression data from a set of 10 MAPK-specific genes predicts MEK inhibitor sensitivity. **a** MPAS and corresponding cell viability data (relative to vehicle (DMSO) control on day of treatment) in response to 0–10 μM cobimetinib treatment for 72 h in melanoma (MEL-JUSO), NSCLC (NCI-H2347), and pancreatic (HUP-T4) cell lines. Error bars represent standard deviation across triplicate samples. Correlations of MPAS vs. relative cell growth for each cell line are also shown. **b** Rank correlation coefficients (filtered for *P* < 0.05) between MPAS and sensitivity (mean viabilities) to MAPK and PI3K signaling pathway inhibitors across cell lines from breast (BRCA) (*n* = 36), lung (*n* = 95), CRC (colorectal) (*n* = 50), melanoma (*n* = 40), pancreatic (*n* = 36), and all indications (pan-cancer) (*n* = 506). **c** E-Net model-based prediction of cobimetinib sensitivity (1-MV Predicted) for 40 independent NSCLC cell lines vs. the actual mean viabilities (1-MV Data) for the same cell lines treated with 0–10 μM cobimetinib for 72 h. MPAS vs. actual cobimetinib sensitivity (1-MV Data) for the same cell lines treated with 0–10 μM cobimetinib for 72 h. **d** 40 NSCLC cell lines were categorized as either “sensitive” IC50 < 1 μM or “resistant” IC50 > 1 μM to cobimetinib. True positive (TPR, TPR = TP/(TP + FN)), false positive rate (FPR = FP/(FP + TN)), and accuracy (ACC = (TP + TN)/N) were computed using median-based classification of the E-Net predictions and MPAS and compared to CTRL score (house-keeping gene expression), *KRAS* mutation status, and mean viability. **e** The cumulative distribution of Spearman correlation coefficients for 45 team’s predictions of MEK1/2 inhibitor (PD184352) sensitivity from the DREAM consortium Drug Sensitivity Challenge, compared to the performance of the elastic net model, (*ρ* = 0.55) mapping to the 36th percentile, and the MAPK score (*ρ* = 0.65), to the 28th percentile

To determine whether a score derived from the expression of these 10 genes could track MAPK-inhibitor-mediated growth suppression (% cell viability), MPAS was calculated at each dose of cobimetinib in the three aforementioned cell lines treated with increasing doses of cobimetinib. As shown in Fig. 1a, cobimetinib treatment resulted in dose-dependent inhibition of MPAS in all three cell lines. The IC_50_ values of cell growth inhibition and MPAS inhibition of three cell lines were similar (Fig. 1a, see table). Note that while the absolute magnitude and range of MPAS values varied between the three cell lines, the relative changes mediated by cobimetinib treatment correlated well with the growth suppression (Fig. 1a).

Given the difference in baseline MPAS values between these three cell lines, we next queried whether such differences in baseline values are predictive of MAPK inhibitor sensitivity. To do so, we examined sensitivity to various signaling pathway inhibitors as measured by mean viability (MV) across a large cancer cell line collection (Fig. 1b; Supplementary Table 2).^26^ Across all cell lines combined (Pan-cancer, *n* = 506), as well as in breast, lung, colorectal, and melanoma subgroups, cell line sensitivity correlated with MPAS for both MEK and BRAF inhibitors but not inhibitors targeting phosphoinositide-3 kinase (PI3K) (GDC-0941), AKT (GDC-0068), or other pathways (data not shown). Interestingly, there was a modest positive correlation between resistance to AKT inhibition (GDC-0068) and MPAS in breast cancer, supporting reports that, in certain subsets of this disease, the MAPK signaling pathway may mediate resistance to AKT inhibition.^27^ Of note, hierarchical clustering separated the cancer indications into two classes: those in which MPAS predicted MEK1/2 inhibitor sensitivity (breast, lung, and colorectal), and those in which it did not (pancreatic and melanoma).

As the pancreatic and melanoma cell lines were broadly sensitive to MAPK pathway inhibition they showed minimal variance in both MPAS and in sensitivity to MEK pathway inhibitors (i.e., MV), leading to the absence of significant correlations (Supplementary Table 3). Conversely, due to the selectivity of vemurafenib for only *BRAF*^V600^-positive melanoma, there was greater variance in sensitivity to this drug across the melanoma cell lines leading to a significant correlation of sensitivity with MPAS.

### Performance of MPAS is comparable to other genome-based predictive models of MEK1/2 inhibitor sensitivity

As MPAS is derived from the expression of 10 pre-specified genes, we wanted to compare our methodology to that of comprehensive multi-gene predictors. While there is no established gold-standard method for predicting drug sensitivity from genomic data,^28^ elastic-net regression has been widely applied to such problems and generally performs well in comparison to other statistical methods.^26,29^ These models identify the most predictive features from the data and combine these features into multivariate linear models to predict drug sensitivity measurements in cell lines or patient samples. We therefore trained an elastic-net regression (hereafter referred to as E-Net) model to predict the sensitivity of a large panel of cell lines to the clinically approved MEK1/2 inhibitors cobimetinib and trametinib.^26^ Cross-validation of the E-Net model for cobimetinib and trametinib gave *R* values of 0.65 and 0.7, respectively (Supplementary Figure 2), comparable to results of other such models.^29^ Predictive features that were identified by the model included a cluster represented by high PHLDA1 (Supplementary Table 4), along with SPRY2 and 4, DUSP6, CCND1, and EPHA2, which are included in MPAS (Supplementary Table 5).

To validate model predictions and compare accuracy of the E-Net model to MPAS, 40 NSCLC cell lines that had not been used for model training were tested for cobimetinib sensitivity (Supplementary Table 6). We computed rank correlations between the measured sensitivities (MV) vs. predictions for the E-Net model and MPAS, as shown in Fig. 1c. Predicted values from the E-Net model correlated with the actual sensitivity (Spearman’s correlation coefficient, *ρ* = 0.55, *P* = 4.5 × 10^−4^). However, the correlation between MPAS and measured MV was more significant (*ρ* = 0.65, *P* = 1.6 × 10^−5^).

To further assess the accuracy of the different approaches in classifying MEK1/2 inhibitor sensitive vs. resistant cell lines, we categorized cell lines with IC_50_ values <1 μM as “sensitive” and >1 μM as “resistant” based on the clinical serum concentration of cobimetinib (0.1–1 µM).^30^ Predicted sensitivities using either MPAS or E-Net gene predictors were likewise categorized as “sensitive” or “resistant” using a median cut-off. True positive and false positive rates were then calculated for MPAS, the E-Net model, and *KRAS* mutation status. A negative control score (CTRL score) derived from four housekeeping genes (*MLH1*, *SMARCA4*, *U2AF*, *CLTC*) was established as a reference (Fig. 1d). The accuracy of using *KRAS* mutation status (55%) was only slightly better than using a CTRL score, which was equivalent to random chance (50%). In the 40 cell lines that were tested, all the *KRAS*-mutant cell lines were sensitive to cobimetinib (i.e., the false positive rate, FPR = 0). However, there was a high false negative rate given that many *KRAS* wild-type cell lines were equally sensitive to cobimetinib (see Materials and methods for calculations). MPAS and the E-Net model had identical accuracies (72%), therefore predictions derived from either applying advanced statistical models to whole transcriptome expression data (E-Net model) or from compiling known biological knowledge into a simple score (MPAS) converge on a surprisingly similar outcome.

To further benchmark the predictive accuracy of MPAS against other gene expression-based predictors of drug sensitivity, we used data generated in the DREAM Drug Sensitivity Prediction Challenge, which engaged 45 bioinformatics teams using gene expression data from 32 breast cancer cell lines to predict sensitivity of 18 blinded cell lines to drugs (including the MEK1/2 inhibitor PD184352).^28^ While the set of cell lines and MEK1/2 inhibitor used were different, the results from DREAM still serve as a reasonable benchmark to compare MPAS against a variety of world-class methodologies. The cumulative distribution of Spearman correlation coefficients for all 45 teams’ predictions of MEK1/2 sensitivity are shown as a solid line in Fig. 1e. Onto this, we overlaid results from our predictions, with the E-Net model (*ρ* = 0.55) mapping to the 36th percentile and MPAS (*ρ* = 0.65) to the 28th percentile. Accordingly, MPAS appeared to perform better than two-thirds of the predictions submitted to this competition. Overall, these results show that MPAS, despite being derived from the expression of only 10 genes, and without the use of regression coefficients or more advanced statistical techniques, predicted sensitivity to MEK inhibition with better or comparable accuracy to using *KRAS* mutational status or to other genome-based, multivariate predictive models, respectively.

### MPAS is heightened in melanoma compared to other tumor types and does not correlate with RAS/RAF mutation status

**Fig. 2:**
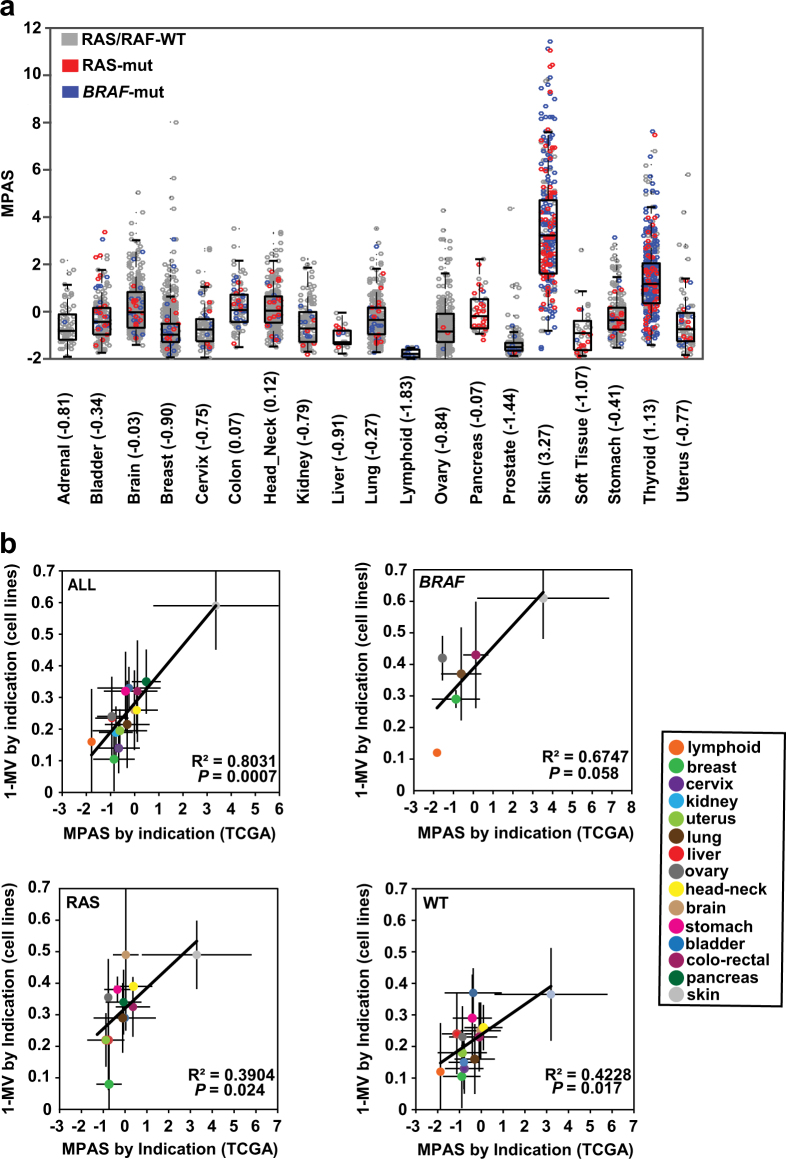
MPAS derived from tumors are the highest in tissues and cell lines sensitive to MAPK inhibitors. **a** MPAS across a panel of different tumor types available in the TCGA database (*n* = 7366), normalized such that 0 represents the average value across all tumors. Tumors are highlighted as follows: RAS/RAF WT (gray), *BRAF*-mutant (blue), and RAS-mutant (*HRAS*, *KRAS*, *NRAS*) (red). Error bars represent standard deviation from the mean, and boxes represent the median, 25th, and 75th percentiles. Median MPAS values are shown in parenthesis by each tissue type. **b** Average MPAS across multiple tumors from TCGA (*n* = 7366) classified by tissue type vs. average mean viability (1-MV) for cell lines of the same tissue type. This was done for all samples, *BRAF*-mutant, RAS-mutant (*HRAS*, *KRAS*, *NRAS*), and wild-type (no RAS/RAF mutations) samples individually. Error bars show standard deviation

To assess the relevance of MPAS in patient tumor specimens, we computed MPAS utilizing gene expression data from a panel of 7366 primary tumors representing 19 different tumor types in The Cancer Genome Atlas (TCGA; Fig. 2a; Materials and methods). Skin cancer (melanoma) and thyroid cancers exhibited the two highest levels and widest distributions of MPAS (medians 3.27 and 1.13, respectively), whereas head and neck, colon, brain, pancreatic, and lung cancers represented the next tier of MPAS (medians of 0.12, 0.07, −0.03, −0.07, and −0.27, respectively). There were no clear associations between MPAS and *BRAF* (blue) or RAS (*KRAS*, *NRAS*, and *HRAS*) (red) mutation status for any indication analyzed (Fig. 2a). This highlights the disconnection between mutational status and MAPK pathway activity and is similar to other published observations that ERK activation (phospho-ERK) does not correlate with either RAS or *BRAF* mutational status in CRC or melanoma tumor tissues.^31–34^

### Primary tumor MPAS correlates with corresponding cell line sensitivity to MAPK inhibition

To investigate how MPAS derived from clinical tumor specimens related to MEK1/2 inhibitor sensitivity across tissue types, we examined the correlation between the median MPAS of tumor specimens from each indication (Fig. 2a) with the median cobimetinib sensitivity (i.e., MV) of cancer cell lines derived from corresponding tissue types (Fig. 2b, Materials and methods, *y* axis denotes 1-MV). Median MPAS of each tissue type was indeed strongly correlated with the median cobimetinib sensitivity of cell lines from the same tissue type (Fig. 2b, Spearman *ρ* = 0.77, Pearson *R*^*2*^ = 0.80). Skin cancer (melanoma) had the highest median MPAS of all the clinical samples, and this was associated with the greatest sensitivity to cobimetinib in cell line studies (Fig. 2b), supporting the clinical observation that melanoma is the cancer type most uniformly susceptible to single-agent MAPK pathway inhibition.^35,36^

When each indication was classified further according to *BRAF*, RAS mutation status, or wild-type (no RAF or RAS mutations), the strength of the correlations appeared to diminish, though still significant, with skin cancer remaining the most sensitive indication to cobimetinib treatment (Fig. 2b).

### MPAS correlates with PFS of *BRAF*^V600^-positive melanoma patients treated with vemurafenib

Approximately 50% of *BRAF*-mutant melanomas initially respond to vemurafenib alone as first-line therapy.^4^ MPAS in skin cancer (melanoma) tissues that were analyzed from TCGA varied widely (−1.5 to 2) and independently of mutational status (Fig. 2a), suggesting that dependence on MAPK signaling in these tumors is highly variable even within the *BRAF*-mutant population. Tumors from melanoma patients in the vemurafenib monotherapy arm of the coBRIM clinical trial have been shown to separate into two clusters based on gene expression profiles. Patients who had tumors with a “cell cycle” gene signature, indicative of high proliferation, did significantly worse (median PFS: 3.48 (95% confidence interval: 2.83–3.94)) than those patients with tumors who had an “immune” signature, characterized by high expression of immune-regulatory genes and CD8+ T-cell infiltration (median PFS: 9.05 (6.21–15.01)).^37^

**Fig. 3:**
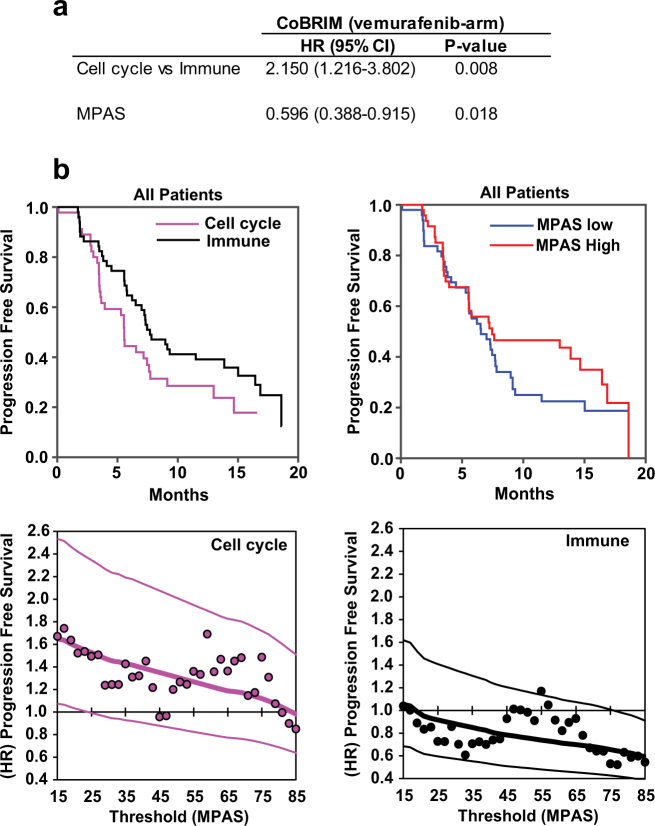
MPAS correlates with response to vemurafenib in *BRAF*-mutant melanoma. **a** Multivariate Cox proportional hazard regression model parameterized using both “cell cycle” and “immune” tumor subtypes and MPAS for tumors from melanoma patients treated with vemurafenib in the control arm of the CoBRIM clinical trial. Hazard ratios (HRs), 95% confidence intervals, and associated *P*-values associated with each feature are shown. **b** Kaplan–Meier curves for progression-free survival (PFS) were plotted according to cell cycle vs. immune or MAPK-high vs. -low median classification for patients enrolled in the vemurafenib arm of coBRIM (median MPAS = 0.08). KM scans of the relative HR as a function of MPAS, using a 15-percentile sliding window for both cell cycle and immune are also shown. Lines represent model-predicted HRs and 95% confidence intervals (compared to all patients), and dots are the HRs computed from the data at individual points

In order to explore whether applying MPAS could further improve the prediction of survival outcome of *BRAF*-mutant patients, we used the gene expression data to develop a multivariate Cox proportional hazard model with MPAS and the published cell cycle vs. immune categorization as two features and PFS as the measure of outcome. The “cell cycle” vs. “immune” tumor subtype was indeed a significant determinant of responsiveness to vemurafenib as published (hazard ratio (HR) = 2.15; *P* = 0.0085; Fig. 3a).^37^ However, *BRAF*-mutant tumors with higher MPAS (and therefore higher MAPK pathway signaling) did significantly better than those with low MPAS across all patients on vemurafenib (HR = 0.596; *P* = 0.018; Fig. 3a, b). Furthermore, we separated the patient subgroups by “cell cycle” vs. “immune” classification and performed a Kaplan–Meier scan to assess the relationship between MPAS and PFS. That is, using a window covering 15% of the population, we scanned across the range of MPAS values and computed the HRs between successive sub-population and remainder. A similarly negative relationship was observed in both subgroups, which illustrates the correlation between MPAS and vemurafenib clinical efficacy, regardless of the tumor microenvironment (Fig. 3b). However, further validation of MPAS as a clinically predictive biomarker was hampered by the lack of a control arm in this trial or other randomized, controlled clinical datasets from trials assessing MAPK pathway inhibitors. Additional clinical studies designed to prospectively validate the utility of MPAS as a predictive biomarker in the future will thus be needed. As such, we subsequently explored the potential of MPAS as a prognostic biomarker across multiple available clinical datasets.

### MPAS is prognostic of poor survival in both resectable and metastatic CRCs

**Fig. 4:**
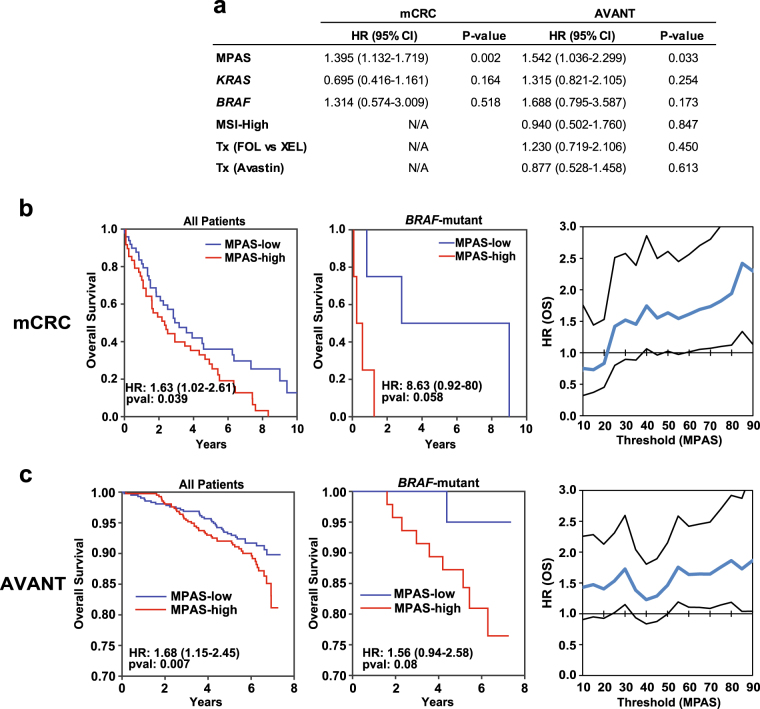
MPAS is a negative prognostic for survival in colorectal cancer. **a** Multivariate Cox proportional hazard regression models for both metastatic CRC samples (mCRC) and for samples from AVANT adjuvant phIII trial parameterized using MPAS, *KRAS*, and *BRAF* mutation status (mCRC) in addition to MSI status and treatment condition; FOLFOX vs. XELOX (Tx FOL vs. XEL) and Avastin for the AVANT samples. Hazard ratios, 95% confidence intervals, and associated *P*-values associated with each feature are shown. Kaplan–Meier curves of overall survival (OS) were plotted using the median value of MPAS to classify **b** mCRC patients or **c** AVANT patients as either MAPK-high vs. -low for all patients or for patients with *BRAF* mutations. Cox proportional hazard regression models were used to fit each treatment arm separately, using MPAS as a univariate predictor of overall survival. Hazard ratios, 95% confidence intervals, and associated *P*-values for MPAS as a univariate predictor are shown within each graph. Kaplan–Meier scans showing hazard ratios of overall survival (OS) over a range of thresholds for classifying MAPK-high vs. -low patients from **b** mCRC patients or **c** AVANT patients are also shown. Blue line represents the hazard computed at each threshold, and black lines show 95% confidence intervals

MAPK signaling is known to play an important role in the development of CRC and in responsiveness to EGFR inhibitor treatments.^38^ Furthermore, CRC patients with *BRAF* and/or RAS family mutations may have an increased risk of disease recurrence and overall poorer survival outcomes.^39^ To evaluate the association between MPAS and survival outcomes in CRC, we analyzed tumor samples from patients with metastatic CRC (mCRC) and resected tumor specimens from CRC patients enrolled in the AVANT phase III adjuvant trial (bevacizumab as a single agent or in combination with either FOLFOX4 or XELOX standard adjuvant therapies^40^). Of note, the AVANT trial demonstrated no significant difference in overall survival between bevacizumab as a single agent or in combination with adjuvant therapies. As such, data were combined for subsequent analysis. For both mCRC and AVANT datasets and across all patients, high MPAS was associated with significantly worse overall survival, whereas *KRAS*/*BRAF* mutational status, microsatellite instability status, and treatment regimens were not (Fig. 4a). Focusing specifically on *BRAF-*mutant patients, who are known to show some responsiveness to MAPK inhibitor therapy,^10^ patients with high MPAS trended toward poorer survival, albeit not reaching statistical significance due to the low frequency of *BRAF* mutations (mCRC: HR = 8.63, *P* = 0.059, *n* = 8; AVANT: HR = 1.56, *P* = 0.08, *n* = 68, Fig. 4b, c). MPAS-high patients within this genetic sub-population were also significantly more likely to die in comparison to the MPAS-low patients (binomial test, *P* = 2.6 × 10^−4^); however, this prognostic effect was not significant in the *KRAS*-mutant or *KRAS/BRAF*-wild-type patients (Supplementary Figure 3b, c). Notably, HRs based on individual MAPK genes rather than the composite MPAS were lower, supporting the use of the aggregate signature as a more robust clinical biomarker (Supplementary Figure 3d) than any single constituent gene.

To assess the relationship between biomarker threshold and survival hazard in mCRC and adjuvant CRC (AVANT) patient populations, we performed a Kaplan–Meier scan by computing HRs for MPAS-high vs. -low patients and using a threshold varying from the 10 to 90 percentile. In mCRC patients, the relationship evidently increased monotonically, whereby patients with increasingly higher MPAS did progressively worse (i.e., HR = 2.4, *P* = 0.003 at an 85th-percentile cutoff) (Fig. 4b). Such a relationship was modest in the AVANT patient population, in that MPAS-high patients fared worse regardless of the cut-off chosen (Fig. 4c).

### MPAS is also prognostic of poor survival in adrenal cancer and HER2+ breast cancer

Although adrenal cancers have a low prevalence of RAS/RAF family mutations,^41^ poor overall survival in adrenocortical carcinoma patients has been correlated with MAPK pathway activity based on a recently developed 100-gene MAPK transcriptional signature.^24^ Likewise, despite the low prevalence of RAS/RAF family mutations in breast cancer, MAPK signaling is thought to be active in some breast cancer subtypes, e.g., triple-negative breast cancer (TNBC), due to *KRAS* or *BRAF* amplification.^42^ In addition, a recent study showed that a significant fraction of HER2+ cell lines are dependent on MAPK signaling for proliferation and survival.^43^ We therefore assessed whether MPAS is prognostic in these indications in which the activation state of the MAPK pathway seems to be independent of RAS/RAF mutational status.

**Fig. 5:**
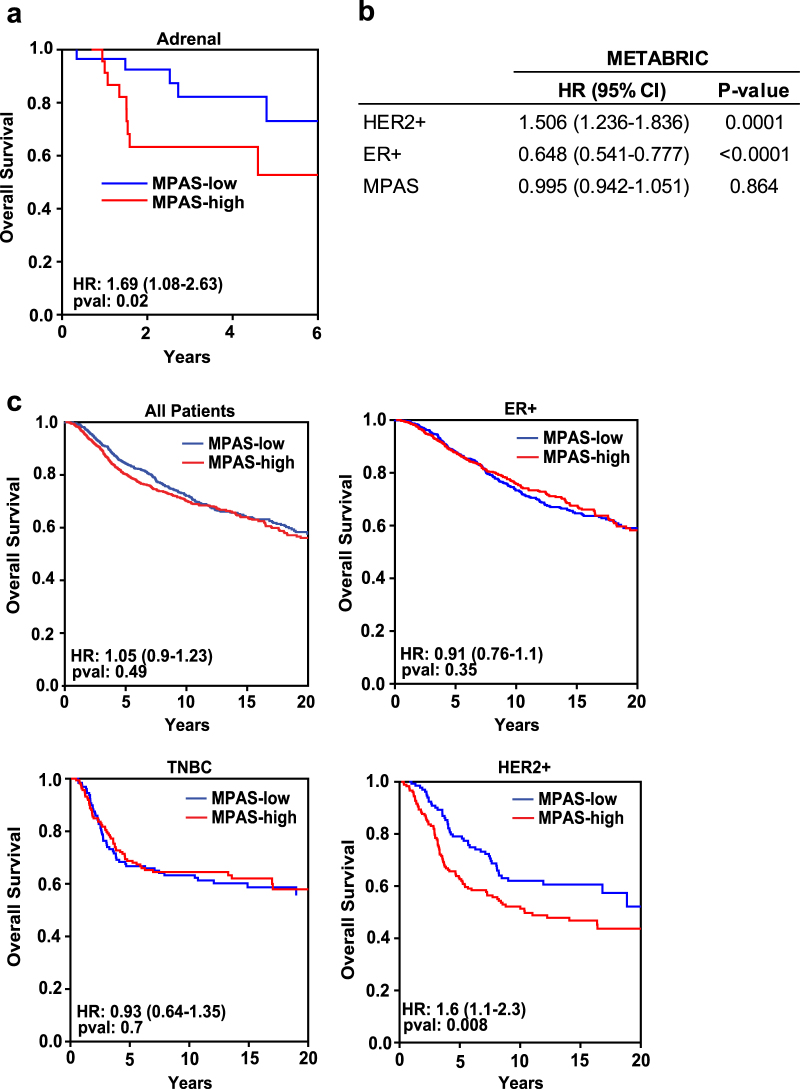
MPAS is a negative prognostic of survival in adrenal carcinoma and HER2+ breast cancer. **a** Kaplan–Meier curves of overall survival were plotted using the median value of MPAS to classify adrenal cancer patient samples from TCGA. **b** Multivariate Cox proportional hazard regression model parameterized using HER2 and ER status (as defined by IHC) and MPAS. Hazard ratios, 95% confidence intervals, and associated *P*-values associated with each feature are shown. **c** Kaplan–Meier curves of overall survival were plotted using the median value of MPAS to classify all patients, ER+ patients, triple-negative (TNBC) patients, and HER2+ patients. Hazard ratios, 95% confidence intervals, and associated *P*-values associated with each subtype are shown

To this end, adrenal tumors from TCGA were classified by MPAS, and in agreement with the published data, those patients with high MPAS had significantly poorer prognosis than those with low MPAS (HR = 1.69, *P* = 0.02) (Fig. 5a). Next, we analyzed survival and expression data from the METABRIC study that contains sequencing data for >2000 primary breast tumors coupled with patient outcome data and classification into ER+, HER2+ or triple-negative (TNBC) subtypes.^44^ In agreement with previous reports, HER2+ status conferred poorer survival outcomes whereas ER+ status conferred a better prognosis (Fig. 5b).^45^ While among all patients, ER+ patients and TNBC patients, we observed that MPAS had no significant prognostic value, in HER2+ breast cancer patients, high MPAS conferred significantly worse overall survival (HR = 1.6 (1.1–2.3); *P* = 0.008), suggesting that variable MAPK signaling dependence in this subgroup has an impact on prognosis (Fig. 5c).

A previous report demonstrated that cellular dependency on PI3K or MAPK signaling in HER2+ breast cancer could be predicted by RNA and protein expression levels of EGFR, ERBB3/HER3, and CDKN1B/p27.^43^ We therefore assessed associations between MPAS and EGFR, ERBB3, or CDKN1B transcript levels (Supplementary Figure 4). High EGFR expression in the HER2+ breast cancers was significantly associated with high MPAS (*ρ* = 0.42, *P* = 7 × 10^−14^), while HER3 and p27 expression were negatively associated with MPAS (*ρ* = −0.21, *P* = 6 × 10^−4^ and *ρ* = −0.11, *P* = 0.076, respectively) in this subgroup, implying that higher relative expression of EGFR to ERBB3 may be responsible for the higher MAPK signaling and poorer survival outcomes.

## Discussion

The MAPK pathway may be activated by mutations in RTK and RAS/RAF oncogene family across many human cancer types. While these mutations are often used as predictive biomarkers for MAPK targeted therapies, other genetic alterations (e.g., SHP2 mutations) and epigenetic mechanisms may also contribute to hyperactivation of this oncogenic pathway.^46^ In addition, the functional activity of RAS/RAF mutations can be modified by the cellular context, including compensatory feedback through the PI3K pathway, which could alter cellular dependence on MAPK signaling. Therefore, a downstream readout of pathway signal flux based on direct transcriptional targets might better represent the extent of pathway activity. As such, we assessed whether the expression of a core set of MAPK genes would be sufficient to capture the extent of MAPK pathway activation and therefore be of use in the clinic. The 10 genes that constituted MPAS have already been identified in other published gene expression signatures that correlate with MAPK inhibitor sensitivity.^17,22–24^ Some were also identified by the E-Net prediction model of cobimetinib sensitivity described in this report.

Our analysis showed that MPAS varied across tumor tissue types independently of RAS or *BRAF* mutational status, supporting the idea of a potential disconnection between mutation status and the activity of the MAPK pathway (Fig. 2a) and highlighting the inadequacy of using solely RAS/RAF mutational status as predictive biomarkers in clinical trials of MEK1/2 inhibitors.

In support of our hypothesis, MPASs derived from cell lines correlated with sensitivity to MAPK pathway inhibitors across multiple indications and those derived from patient specimens were highest in tumor types that are known to be sensitive to MAPK pathway inhibition in the clinic (Figs. 1b and 2a). Furthermore, the predictive accuracy of MPAS was similar to, or better than, that of more complex comprehensive models and algorithms that would be significantly more difficult to translate clinically (Fig. 1c–e).

Melanoma had the highest levels of MAPK activity relative to other tissue types (measured by median MPAS) in our study, consistent with the robust clinical activity of BRAF and MEK1/2 inhibitors observed in this indication (Fig. 2a). However, even within the *BRAF*-mutant skin cancer (melanoma) population, there existed a wide range of, and perhaps dependency on, MAPK signaling that may partially explain some instances of unresponsiveness to vemurafenib in melanoma.

In tumors from both mCRC and from curatively resected Stage III CRC in an adjuvant setting (AVANT),^40^ high MPAS was significantly correlated with poorer outcomes, with a trend for wider separation in patients within the *BRAF*-mutant subgroup, albeit less statistically significant due to smaller patient number (Fig. 4). It was also noted that *BRAF-*mutant CRC had lower MPAS compared to that of melanoma and thyroid cancer (Fig. 2), which may provide some underlying mechanisms for the poorer outcomes observed in *BRAF*-mutant CRC patients treated with BRAF or MEK1/2 inhibitor as single agent or in combination.^10,12^

In adrenal and breast cancers, RAS and RAF mutations are rare. However, high MPAS in adrenal tumors (Fig. 5a) and in a subset of HER2+ breast tumors (Fig. 5c) correlated with poorer overall survival, again indicating that direct measurement of MAPK signaling output through gene transcription may have prognostic value and, in the case of breast cancer, provide additional insight to the biological context of an already well-defined subtype.

Potential challenges facing the use of MPAS in the clinic may include the low or variable tumor content of patient biopsies, lack of uniform standard operating procedures to measure gene expression, and the accurate translation of MPAS derived from preclinical models into clinically relevant thresholds, although this may still be considerably easier than defining thresholds for multiple individual genes.^17,47^ Note that the absolute value of the score depends on not only the cell biology but also the assay characteristics and normalization. Thus it is likely not possible currently to directly translate absolute MPAS thresholds from preclinical experiments or between different clinical studies. This is exemplified by examining the variation in the median and distribution of MPAS scores from the five clinical studies reported (Supplementary Table 7). Absolute thresholds in the clinic will thus need to be determined based on future prospective studies and will likely be both indication- and assay-specific and may differ between alternate MAPK targets (i.e., RAF, MEK, or ERK inhibitors) or molecules (i.e., cobimetinib vs. trametinib). However, as MPAS is derived from the aggregated expression of only 10 genes, there is potential for its development as a clinically actionable biomarker. MAPK signaling is of course only one potential component of disease outcome. We envision MPAS may ultimately be realized as one component of a multivariate predictor, perhaps comprising also of mutations and other pathway and immunological signatures to construct a comprehensive precision medicine platform. Future randomized clinical trials with paired genomic profiles are required to validate the predictive or prognostic utility of MPAS in additional indications or disease subgroups where MAPK signaling plays an important role.

In conclusion, by aggregating gene expression measurements from 10 highly conserved MAPK transcription targets, we have developed MPAS that predicts the sensitivity to MAPK pathway inhibitors across cell lines and is associated with the sensitivity of tumor samples to vemurafenib in the CoBRIM trial. Retrospective analysis of tumor specimens from CRC, HER2+ breast, and adrenal cancers also revealed the potential prognostic utility of MPAS. These findings support the integration of this methodology in clinical trials prospectively for further assessment.

## Materials and methods

### Chemicals

Cobimetinib, vemurafenib, and compounds with the GDC-prefix were manufactured at Genentech (South San Francisco, CA, USA). The PD0325901 MEK inhibitor was purchased from Selleck Chemicals (Houston, TX, USA).

### Cell lines

Cell lines were obtained from the Genentech cell line repository (gCSI)^26^ and cultured according to the supplier’s specifications. The NCI-H2347 cell line (CRL-5942, ATCC, Manassus, VA, USA) and the MEL-JUSO cell line (ACC 74, DSMZ, Braunschweig, Germany) were routinely cultured in a growth medium of RPMI-1640 with 10% fetal bovine serum (FBS). The HUP-T4 cell line ACC 223 (DSMZ, Braunschweig, Germany) was routinely cultured in a growth medium of Minimum Essential Medium with 10% FBS.

### Cell viability assays

Cells were seeded in 96-well plates and treated in triplicate for 72 h with 0–10 μM concentrations of the relevant drug diluted in the appropriate growth medium (please see above). Cell viability was measured using CellTiter-Glo® reagent per the manufacturer’s instructions (Promega, Madison, WI, USA) and ATP measurements were read using an Envision reader (PerkinElmer, San Jose, CA, USA). Cell viability measurements were corrected to the day 0 viability (day of treatment) as measured by CellTiter-Glo® and plotted relative to the dimethyl sulfoxide control (100% viability).^48^ With this method, cell growth after treatment can be compared to that before treatment (i.e., zero percent growth represents cell stasis and negative percent growth represents cell death). Three replicate wells per treatment were used, such that, at 5% standard deviation, viability differences of >9% should be detectable with a power of 80%. Data shown represent three independent experiments.

### Baseline MAPK gene expression across lung, colorectal, and melanoma patient samples

Formalin-fixed paraffin-embedded (FFPE) tissue blocks were procured from melanoma (*n* = 50), CRC (*n* = 50), and lung patients (*n* = 100) (for vendors, see Supplementary Table 8). In all, 250 ng RNA from these samples was run on a 10-gene, MAPK-specific Nanostring panel (Nanostring Technologies, Seattle, WA, USA), (Supplementary Table 9). Data were analyzed using the Nanostring nSolver software. Raw counts <10 were considered below background (geometric mean of the negative controls).

### MAPK Pathway Activity Score

MPAS for each cell line, tumor, or patient sample was derived from expression data for 10 MAPK-specific genes (PHLDA1, SPRY2, SPRY4, DUSP4, DUSP6, CCND1, EPHA2, EPHA4, ETV4, and ETV5). Gene expression was measured using either RNA-Seq (Illumina, Hayward, CA, USA) sequencing (reads per counts per million)^49^ or the MAPK-specific Nanostring panel (Supplementary Table 9). MPAS was computed as MAPK activity = $\frac{{\mathop {\sum }\nolimits^ z_i}}{{\sqrt n }},$ where *z*_*i*_ is the *z*-score of each gene’s expression level and *n* is the number of genes comprising the set (i.e., *n* *=* 10). For the Nanostring data, gene expression levels were normalized by sample, using a set of housekeeping genes, and across samples for the RNA-Seq data. The score is thus a relative metric of MAPK gene expression, the absolute values of which will depend on the assay characteristics and choice of normalization, in addition to the underlying biology. As such, MPAS values should be interpreted as relative to other samples within an experimental or clinical context.

### Elastic-net model

An E-Net regression model was created to predict cobimetinib and trametinib mean cell viability using RNA-seq data from >26,255 genes from 189 lung, pancreatic, and colon cell lines included in gCSI.^26,49^ This model was trained on gene expression features with alpha = 0.5 and optimal lambda chosen by 5-fold cross validation. After applying a variance stabilizing transformation, gene expression was normalized to a normal distribution (mean = 0, StDev = 1) to remove absolute expression bias.^49^ To cross-validate the model, predicted MVs of the cell lines used to train the model were correlated with experimentally derived MVs to both trametinib and cobimetinib (Supplementary Table 10).

### Accuracy comparison of each of the predictors

To assess the overall accuracy of each predictor (E-Net, MPAS, and *KRAS* status), cell lines in the validation set (40 NSCLC cell lines) were categorized as either cobimetinib sensitive (IC_50_ < 1 µM) or resistant (IC_50_ > 1 µM) based on the average clinical serum concentration of cobimetinib (0.1–1 µM).^30^ Based on numbers of True Positive (TP) True Negative (TN), False Positive (FP), and False Negative (FN) calls, the true positive rate (TPR = TP/(TP + FN)), false positive rate (FPR = FP/(FP + TN)), and accuracy (ACC = (TP + TN)/N) were then computed using a median-based classifier of the E-Net predictions and MPAS.

### Melanoma patient samples

RNA from 99 pre-treated FFPE tumor samples from patients with *BRAF*^V600^-mutated metastatic melanoma enrolled in the vemurafenib arm of the coBRIM phase III clinical trial^9^ with prior Institutional Review Board (IRB) approval and informed consent were analyzed using a custom melanoma-specific Nanostring expression panel consisting of 800 genes including the 10 MAPK-specific genes constituting MPAS (Supplementary Table 11). Cell cycle-high vs. immune-high subgroups were defined using previously identified signatures^37^ and MPAS-high vs. -low cell lines were categorized using a median cut-off.

### mCRC and AVANT patient samples

FFPE tumor tissue blocks were procured from 74 colorectal patients with metastatic disease (mCRC) and from 1262 curatively resected stage III CRC patients enrolled in the AVANT phIII adjuvant trial^40^ with prior IRB approval and informed consent. Total RNA was run on a CRC-specific custom Nanostring panel that included 5 or 6 of the 10 MAPK-specific genes constituting MPAS as this dataset was intended for other analyses (mCRC set: EPHA4, ETV5, DUSP6, CCND1, and SPRY4; AVANT set: same as mCRC except SPRY2 was used instead of SPRY4 and ETV4) (Supplementary Tables 12 and 13).

### TCGA gene expression and survival data for breast, adrenal (ACC), and melanoma (SKCM) tumors

mRNA expression (RPKM) data for the 10 MAPK genes, as well as patient survival and subtype classifications (for Breast Cancer) were obtained from the BROAD Firehose TCGA portal. Breast cancer data were from the Molecular Taxonomy of Breast Cancer International Consortium (METABRIC) study.^44^

### Statistical methods

Rank-sum tests were used to compute all correlation coefficients. Cox proportional hazard regression models were developed using MPAS as a continuous univariate predictor or as part of a set of multivariate predictors, i.e., mutational status or treatment. Log-rank tests were performed for all Cox hazard regression models. For the CRC data, a Kaplan–Meier scan was performed using MPAS as a discrete predictor, varying the threshold for calling positive/negative over a range from the 10th to 90th percentiles, and HRs were computed along this range. For the melanoma data, a sliding 30th-percentile window was used, and HRs were computed in comparison to the unselected population (i.e., starting with the 0–30th-percentile group (median 15%) vs. the entire population, and increasing the window by 2 points successively across the range to the 70–100th percentile (median 85%)). The E-Net regression model was developed in R, and all computational analyses were performed in the MATLAB v2015b software (Mathworks, Natick, MA).

### Data availability

Expression data for Figs. 1b, 2, and 5a were obtained from the TCGA database. All breast cancer expression data were obtained from the METABRIC database. All other expression data used are shown in supplementary tables as noted in the text.

## Electronic supplementary material

Supplementary figures and tables

## Electronic supplementary material

**Supplementary Information** accompanies the paper on the *npj Precision Oncology* website (10.1038/s41698-018-0051-4).

## References

[1] McCubrey JA (2007). Roles of the Raf/MEK/ERK pathway in cell growth, malignant transformation and drug resistance. Biochim. Biophys. Acta.

[2] Neuzillet C (2014). MEK in cancer and cancer therapy. Pharmacol. Ther..

[3] Holderfield M, Deuker MM, McCormick F, McMahon M (2014). Targeting RAF kinases for cancer therapy: BRAF-mutated melanoma and beyond. Nat. Rev. Cancer.

[4] McArthur GA (2014). Safety and efficacy of vemurafenib in BRAF(V600E) and BRAF(V600K) mutation-positive melanoma (BRIM-3): extended follow-up of a phase 3, randomised, open-label study. Lancet Oncol..

[5] Morris EJ (2013). Discovery of a novel ERK inhibitor with activity in models of acquired resistance to BRAF and MEK inhibitors. Cancer Discov..

[6] Zhao Y, Adjei AA (2014). The clinical development of MEK inhibitors. Nat. Rev. Clin. Oncol..

[7] Qu, X.et al Integrated genomic analysis of colorectal cancer progression reveals activation of EGFR through demethylation of the EREG promoter. *Oncogene* **35**, (2016), https://doi.org.10.1038/onc.2016.170. 10.1038/onc.2016.170 PMC5161754 27270421

[8] Hauschild A (2012). Dabrafenib in BRAF-mutated metastatic melanoma: a multicentre, open-label, phase 3 randomised controlled trial. Lancet.

[9] Ascierto PA (2016). Cobimetinib combined with vemurafenib in advanced BRAF(V600)-mutant melanoma (coBRIM): updated efficacy results from a randomised, double-blind, phase 3 trial. Lancet Oncol..

[10] Corcoran RB (2015). Combined BRAF and MEK inhibition with dabrafenib and trametinib in BRAF V600-mutant colorectal cancer. J. Clin. Oncol..

[11] Long GV (2016). Overall survival and durable responses in patients with BRAF V600-mutant metastatic melanoma receiving dabrafenib combined with trametinib. J. Clin. Oncol..

[12] Kopetz S (2015). Phase II pilot study of vemurafenib in patients with metastatic braf-mutated colorectal cancer. J. Clin. Oncol..

[13] Planchard D (2016). Dabrafenib in patients with BRAF(V600E)-positive advanced non-small-cell lung cancer: a single-arm, multicentre, open-label, phase 2 trial. Lancet Oncol..

[14] Carter CA (2016). Selumetinib with and without erlotinib in KRAS mutant and KRAS wild-type advanced nonsmall-cell lung cancer. Ann. Oncol..

[15] Gandara DR (2013). Oral MEK1/MEK2 inhibitor trametinib (GSK1120212) in combination with docetaxel in KRAS-mutant and wild-type (WT) advanced non-small cell lung cancer (NSCLC): a phase I/Ib trial. J. Clin. Oncol..

[16] Riess, H., et al. Phase II study of the MEK inhibitor refametinib (BAY 86-9766) in combination with gemcitabine in patients with unresectable, locally advanced, or metastatic pancreatic cancer. *J. Clin. Oncol.* **32**, 4129 (2014).

[17] Dry JR (2010). Transcriptional pathway signatures predict MEK addiction and response to selumetinib (AZD6244). Cancer Res..

[18] Wang QT (2004). A genome-wide study of gene activity reveals developmental signaling pathways in the preimplantation mouse embryo. Dev. Cell.

[19] Fearon ER, Spence JR (2012). Cancer biology: a new RING to Wnt signaling. Curr. Biol..

[20] Huang SM (2009). Tankyrase inhibition stabilizes axin and antagonizes Wnt signalling. Nature.

[21] LoRusso PM (2011). Phase I trial of hedgehog pathway inhibitor vismodegib (GDC-0449) in patients with refractory, locally advanced or metastatic solid tumors. Clin. Cancer Res..

[22] Joseph EW (2010). The RAF inhibitor PLX4032 inhibits ERK signaling and tumor cell proliferation in a V600E BRAF-selective manner. Proc. Natl. Acad. Sci. USA.

[23] Pratilas CA (2009). (V600E)BRAF is associated with disabled feedback inhibition of RAF-MEK signaling and elevated transcriptional output of the pathway. Proc. Natl. Acad. Sci. USA.

[24] Schubert, M. K. B., Klünemann, M., Garnett, M. J., Blüthgen, N. & Saez-Rodriguez, J. Perturbation-response genes reveal signaling footprints in cancer gene expression. *Nature Communications* **9**, 20 (2018). 10.1038/s41467-017-02391-6 PMC5750219 29295995

[25] Merchant M (2017). Combined MEK and ERK inhibition overcomes therapy-mediated pathway reactivation in RAS mutant tumors. PLoS ONE.

[26] Haverty PM (2016). Reproducible pharmacogenomic profiling of cancer cell line panels. Nature.

[27] Paplomata E, O’Regan R (2014). The PI3K/AKT/mTOR pathway in breast cancer: targets, trials and biomarkers. Ther. Adv. Med. Oncol..

[28] Costello JC (2014). A community effort to assess and improve drug sensitivity prediction algorithms. Nat. Biotechnol..

[29] Barretina J (2012). The Cancer Cell Line Encyclopedia enables predictive modelling of anticancer drug sensitivity. Nature.

[30] Han K (2015). Population pharmacokinetics and dosing implications for cobimetinib in patients with solid tumors. Cancer Chemother. Pharmacol..

[31] Holck S (2016). Localization of active, dually phosphorylated extracellular signal-regulated kinase 1 and 2 in colorectal cancer with or without activating BRAF and KRAS mutations. Hum. Pathol..

[32] Houben R (2008). Phospho-ERK staining is a poor indicator of the mutational status of BRAF and NRAS in human melanoma. J. Invest. Dermatol..

[33] Levidou G (2012). ERK/pERK expression and B-raf mutations in colon adenocarcinomas: correlation with clinicopathological characteristics. World J. Surg. Oncol..

[34] Yazdi AS, Ghoreschi K, Sander CA, Rocken M (2010). Activation of the mitogen-activated protein kinase pathway in malignant melanoma can occur independently of the BRAF T1799A mutation. Eur. J. Dermatol..

[35] Infante JR (2012). Safety, pharmacokinetic, pharmacodynamic, and efficacy data for the oral MEK inhibitor trametinib: a phase 1 dose-escalation trial. Lancet Oncol..

[36] Rosen LS (2016). A first-in-human phase I study to evaluate the MEK1/2 inhibitor, cobimetinib, administered daily in patients with advanced solid tumors. Invest. New Drugs.

[37] Wongchenko MJ (2017). Gene expression profiling in BRAF-mutated melanoma reveals patient subgroups with poor outcomes to vemurafenib that may be overcome by cobimetinib plus vemurafenib. Clin. Cancer Res..

[38] Shaib W, Mahajan R, El-Rayes B (2013). Markers of resistance to anti-EGFR therapy in colorectal cancer. J. Gastrointest. Oncol..

[39] Richman SD (2009). KRAS and BRAF 606 mutations in advanced colorectal cancer are associated with poor prognosis but do not preclude benefit from oxaliplatin or irinotecan: results from the MRC FOCUS trial. J. Clin. Oncol..

[40] de Gramont A (2012). Bevacizumab plus oxaliplatin-based chemotherapy as adjuvant treatment for colon cancer (AVANT): a phase 3 randomised controlled trial. Lancet Oncol..

[41] Rubin B (2015). Mitogen-activated protein kinase pathway: genetic analysis of 95 adrenocortical tumors. Cancer Invest..

[42] Giltnane JM, Balko JM (2014). Rationale for targeting the Ras/MAPK pathway in triple-negative breast cancer. Discov. Med..

[43] Kirouac DC (2016). HER2+ cancer cell dependence on PI3K vs. MAPK signaling axes is determined by expression of EGFR, ERBB3 and CDKN1B. PLoS Comput. Biol..

[44] Curtis C (2012). The genomic and transcriptomic architecture of 2,000 breast tumours reveals novel subgroups. Nature.

[45] Cheang MC (2009). Ki67 index, HER2 status, and prognosis of patients with luminal B breast cancer. J. Natl. Cancer Inst..

[46] Tajan M, de Rocca Serra A, Valet P, Edouard T, Yart A (2015). SHP2 sails from physiology to pathology. Eur. J. Med. Genet..

[47] de Gramont A (2015). Pragmatic issues in biomarker evaluation for targeted therapies in cancer. Nat. Rev. Clin. Oncol..

[48] Wagle M (2016). A role for FOXO1 in BCR-ABL1-independent tyrosine kinase inhibitor resistance in chronic myeloid leukemia. Leukemia.

[49] Klijn C (2015). A comprehensive transcriptional portrait of human cancer cell lines. Nat. Biotechnol..

