# Supplementary material for: A transcriptional MAPK Pathway Activity Score (MPAS) is a clinically relevant biomarker in multiple cancer types
Source: NPJ Precis Oncol. 2018 Mar 7;2:7. doi: 10.1038/s41698-018-0051-4 (PMC5871852; doi:10.1038/s41698-018-0051-4)
Supplement: Supplementary file 1 — Supplementary figures and tables [file 41698_2018_51_MOESM1_ESM.pdf]

A transcriptional MAPK Pathway Activity Score (MPAS) is a clinically relevant biomarker in multiple cancer types

Marie-Claire Wagle[1,2*], Daniel Kirouac[1,3], Christiaan Klijn[4], Bonnie Liu[2], Shilpi Mahajan[2], Melissa Junttila[5], John Moffat[6], Mark Merchant[5], Ling Huw[2], Matthew Wongchenko[2], Kwame Okrah[7], Shrividhya Srinavasan[2], Zineb Mounir[2], Teiko Sumiyoshi[2], Peter M. Haverty[4], Robert L. Yauch[5], Yibing Yan[2], Omar Kabbarah[2], Garret Hampton[2], Lukas Amler[2], Saroja Ramanujan[3], Mark R. Lackner[2] and Shih-Min A. Huang[2*].

[1]These authors contributed equally to this work, Departments of [2]Oncology Biomarker Development, [3]Pre-Clinical and Translational PKPD, [4]Bioinformatics, [5]Translational Oncology, [6]Biochemical and Cellular pharmacology, [7]Biostatistics, Genentech, 1 DNA Way, South San Francisco, CA 94080, USA.

*CORRESPONDENCE: Marie-Claire Wagle: Genentech, 1 DNA Way, South San Francisco, CA 94080, USA, ph: 650-467-3256, marieclw@gene.com. Shih-Min A. Huang *(current affiliation)*: Bristol-Myers Squibb, 3551 Lawrenceville Princeton, Lawrence Township, NJ 08648, ph: 650-452-2026, alex.huang1@bms.com.

Supplementary Materials

Supplementary Figures

Supplementary Figure 1: Expression and correlation of the 10 MAPK genes that constitute MPAS in colorectal, lung and pancreatic patient samples.

Supplementary Figure 2: Cross-validation of the E-Net model

Supplementary Figure 3: Association of BRAF-mutational status or MPAS with overall survival in patients with mCRC or resectable CRC

Supplementary Figure 4: Spearman's Rank Correlation coefficients between EGFR, CDKN1B, and ERBB3 gene expression, and MPAS for HER2+ breast cancer patients.

Supplementary Tables

Supplementary Table 1: MAPK gene expression in melanoma (MEL-JUSO), lung (NCI-H2347) and pancreatic (HUPT4), % inhibition in response to 0.01-1uM cobimetinib treatment for 72 h.

Supplementary Table 2: MAPK gene expression, MPAS and cell viability in response to multiple MAPK inhibitors across cell lines from multiple indications.

Supplementary Table 3: Standard deviations of either MPAS or actual mean viability across cell lines treated with either MAPK or PI3K/AKT inhibitors.

Supplementary Table 4: Short-list of gene clusters identified by the E-Net model as being predictive of MAPK inhibitor (cobimetinib and trametinib) sensitivity.

Supplementary Table 5: Genes with expression that correlates with the PHLDA1 gene cluster identified by the E-Net model.

Supplementary Table 6: Gene expression, KRAS mutation status and predicted/actual mean viabilities to both trametinib and cobimetinib for cell lines in NSCLC validation set.

Supplementary Table 7: Median, maximum and minimum MPAS values for each clinical study.

Supplementary Table 8: List of vendors from which melanoma, crc and lung patient samples were procured.

Supplementary Table 9: Genes included in the custom MAPK-specific nanostring panel.

Supplementary Table 10: Predicted and actual mean viabilities in response to either trametinib or cobimetinib for cell lines included in the E-Net training set.

Supplementary Table 11: MPAS and PFS of patients included in the vemurafenib arm of the CoBrim trial.

Supplementary Table 12: Individual MAPK gene expression, MPAS and overall survival of patients with metastatic CRC.

Supplementary Table 13: Individual MAPK gene expression, MPAS and overall survival of patients with CRC in the adjuvant setting (AVANT).

Supplementary Figure 1

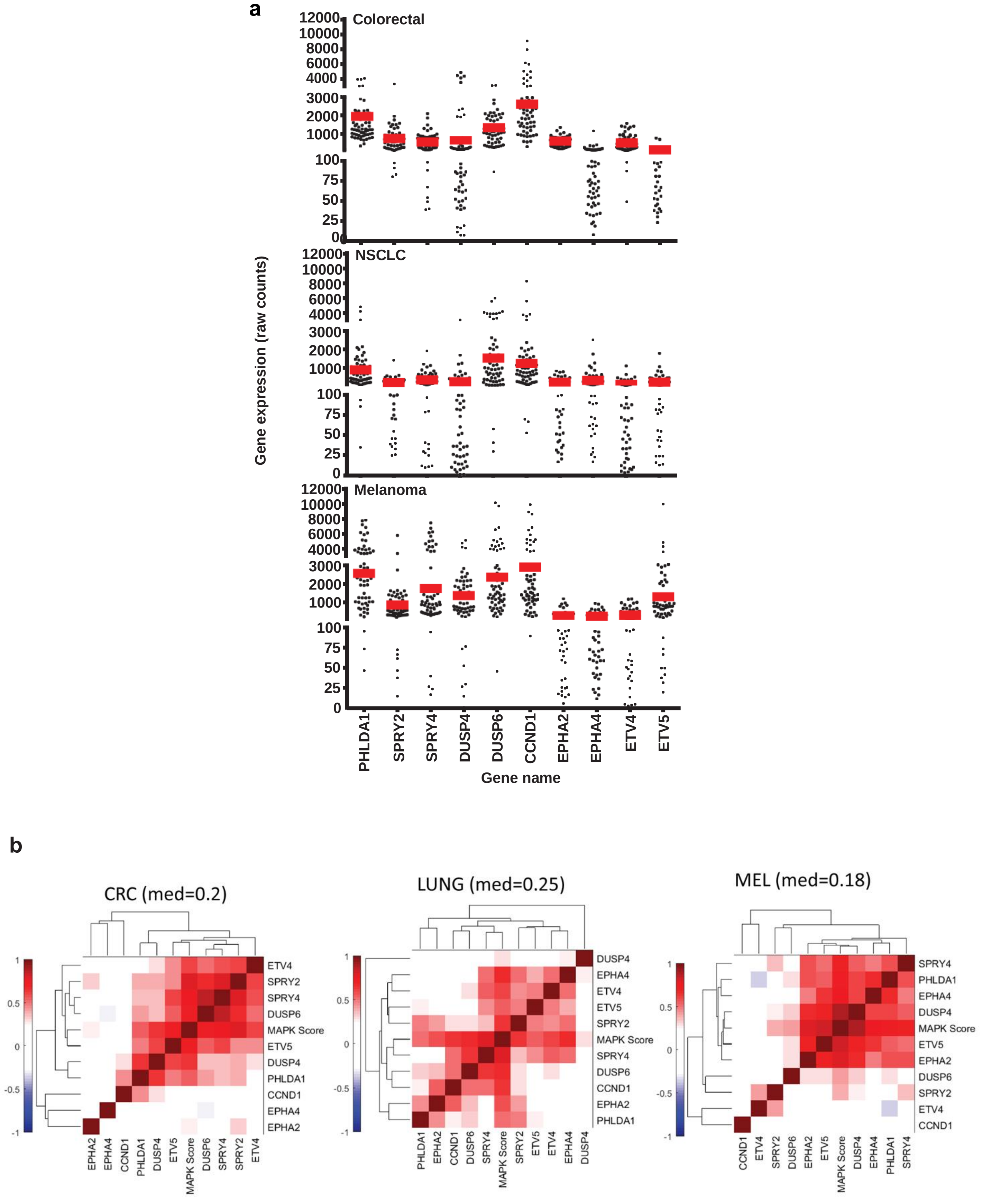

Supplementary Figure 1: Expression and correlation of the 10 MAPK genes that constitute MPAS in colorectal, lung and pancreatic patient samples. (A) Raw gene expresssion values of each gene are shown and red bars represent median values. Raw counts below 10 were considered below background (geometric mean of the negative controls). **(B)** Correlation matrices of the genes constituting MPAS across each tissue type.

Supplementary Figure 2

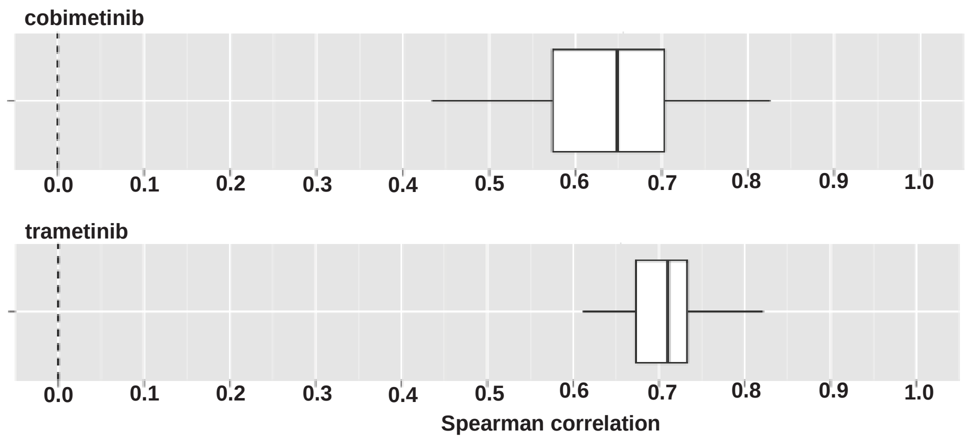

Supplementary Figure 2: Cross-validation of the E-Net model
Predicted mean viabilities of the cell lines used to train the model were correlated with experimentally derived mean viabilities to both trametinib and cobimetinib treatment.

Supplementary Figure 3

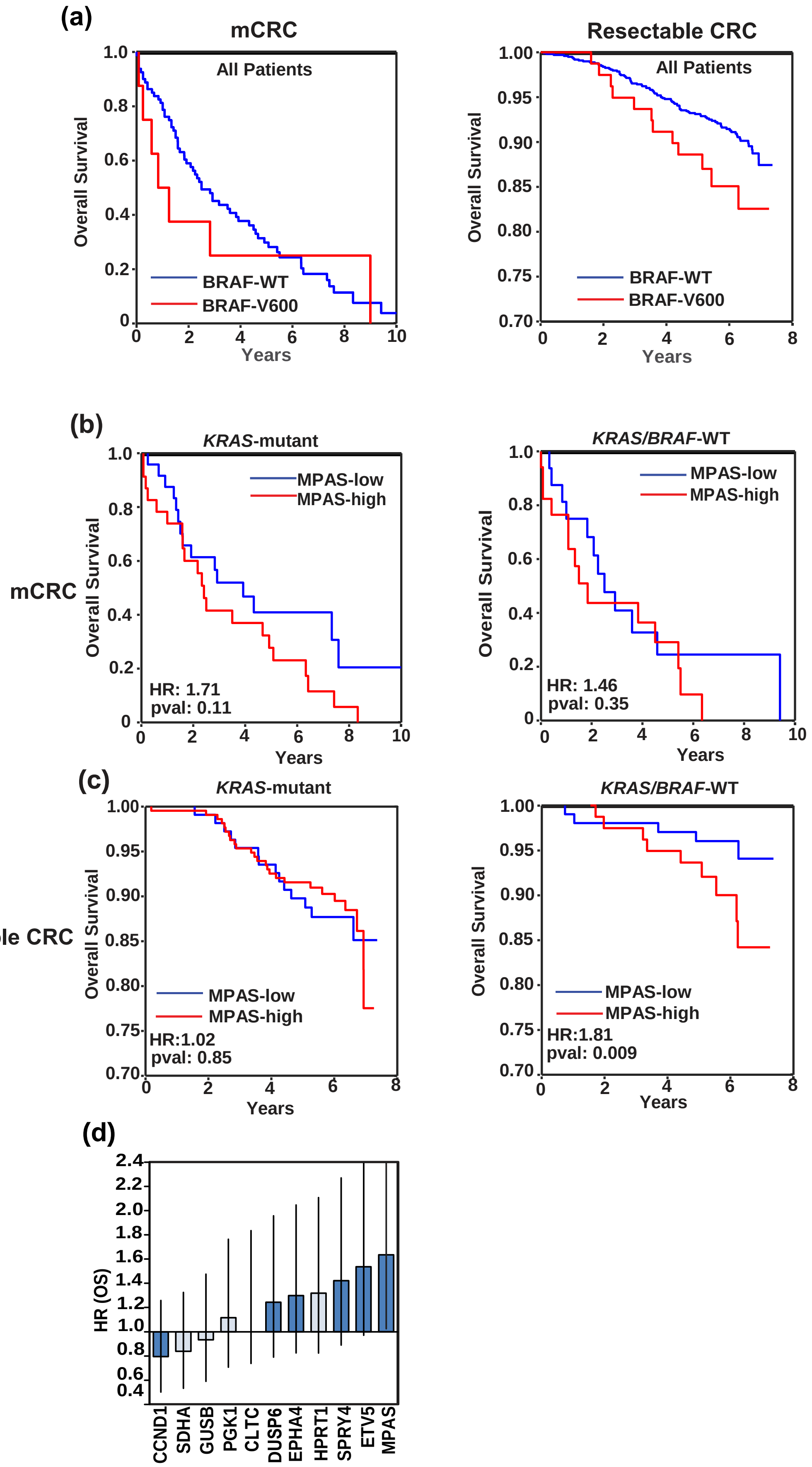

Supplementary Figure 3: Association of BRAF-mutational status or MPAS with overall survival in patients with mCRC or resectable CRC Kaplan-Meier curves for overall survival were plotted according to (a) *BRAF*-mutational status in mCRC and resectable CRC; (b) MPAS high vs low in mCRC in *KRAS*-mutant patients and in *KRAS/BRAF*-wild-type patients; (c) MPAS high vs low in resectable CRC in *KRAS*-mutant patients and in *KRAS/BRAF*-wild-type patients.(d) Hazard ratios based on individual MAPK gene expression.

Supplementary Figure 4

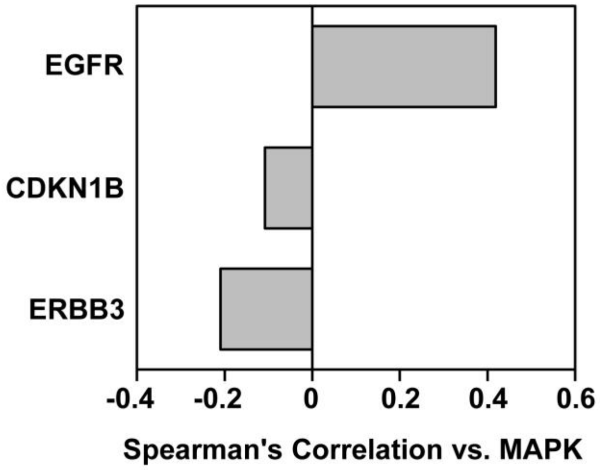

Supplementary Figure 4: Spearman's Rank Correlation coefficients between EGFR, CDKN1B, and ERBB3 gene expression, and MPAS for HER2+ breast cancer patients.

Supplementary Table 1

NCI-H2347	cobimetinib mM			
**MAPK genes included in MPAS**				
**Gene name**	**0.01**	**0.1**	**0.3**	**1**
**DUSP4**	84.009818	35.643594	27.190967	19.840943
**DUSP6**	47.248463	3.6312725	2.0102699	1.6448766
**SPRY2**	97.973703	69.697768	57.526811	56.651999
**SPRY4**	53.397032	11.117733	8.6011996	8.1301907
**ETV4**	75.287801	63.019759	60.80756	52.484966
**ETV5**	91.32767	50.136426	39.706065	38.61466
**EPHA2**	63.371252	22.808825	16.801277	15.679199
**EPHA4**	56.282802	26.257878	24.385499	27.378468
**CCND1**	79.840366	55.029664	49.55298	48.757693
**PHLDA1**	59.920227	16.641753	11.959699	10.954393
**MAPK target genes not included in MPAS**				
**INPP5F**	95.098361	91.2922	93.438092	92.598326
**MAP2K3**	94.577957	79.448325	83.714139	74.641885
**TRIB2**	63.343938	50.281607	36.361494	47.071992
**ETV1**	86.091355	53.854333	55.643203	53.681912

HUP-T4	cobimetinib mM			
**MAPK genes included in MPAS**				
**Gene name**	**0.01**	**0.1**	**0.3**	**1**
**DUSP4**	76.336449	17.050127	11.564996	6.7969414
**DUSP6**	49.301246	4.6825306	2.1822457	2.4345015
**SPRY2**	69.159009	22.820941	18.803954	13.227513
**SPRY4**	49.284206	16.32246	6.9701948	6.4538841
**ETV4**	56.787565	25.751295	11.19171	5.1813472
**ETV5**	59.962687	25.932836	20.149254	7.4626866
**EPHA2**	73.96657	40.20447	33.604622	32.776262
**EPHA4**	67.624009	41.396946	35.555819	35.27643
**CCND1**	85.876056	51.258585	46.229946	46.347097
**PHLDA1**	41.983977	6.9209501	4.8211525	4.7895848
**MAPK target genes not included in MPAS**				
**INPP5F**	98.385829	95.278025	83.795399	86.468214
**MAP2K3**	58.541731	60.330432	27.371702	27.879488
**TRIB2**	104.56906	100.09922	97.721914	96.892113
**ETV1**	77.323287	28.507356	32.956584	28.7047

MEL-JUSO	cobimetinib mM			
**MAPK genes included in MPAS**				
**Gene name**	**0.01**	**0.1**	**0.3**	**1**
**DUSP4**	93.430208	66.927549	40.285665	28.729698
**DUSP6**	57.927467	22.318912	7.1517999	2.7265561
**SPRY2**	100.74749	59.635738	27.617305	10.650026
**SPRY4**	58.016714	12.803481	4.1164814	2.3020591
**ETV4**	89.913793	55.901254	43.510972	65.23511
**ETV5**	139.99269	77.657592	45.770144	40.931847
**EPHA2**	64.568901	41.565031	26.457866	24.064136
**EPHA4**	75.691057	64.444444	55.98916	55.691057
**CCND1**	83.156671	47.593063	27.755948	22.979558
**PHLDA1**	75.101762	59.373121	48.82742	40.521648
**MAPK target genes not included in MPAS**				
**INPP5F**	83.926748	58.14958	43.547277	39.593179
**MAP2K3**	60.836602	56.536133	31.639033	32.63122
**TRIB2**	120.78999	103.25927	68.948157	53.587973
**ETV1**	110.8749	87.382614	65.637116	63.080469

Supplementary Table 1: MAPK gene expression in melanoma (MEL-JUSO), lung (NCI-H2347) and pancreatic (HUPT4), % inhibition in response to 0.01-1uM cobimetinib treatment for 72 h.

Supplementary Table 2

Cell line	Tissue	Individual MAPK gene expression (RPKM)										MPAS	Mean viability to different drugs					
		DUSP4	DUSP6	SPRY2	SPRY4	CCND1	ETV4	ETV5	EPHA2	EPHA4	PHLDA1		GDC0068	GDC0941	Vemu-rafenib	Cobi-metinib	GDC0623	PD032-5901
A172	Brain	3.45	2.01	1.43	2.10	8.49	3.74	3.61	6.56	2.25	4.31	-0.40	0.99	0.9	0.87	0.8	0.86	0.75
CHP-212	Brain	0.06	1.36	6.89	3.51	8.54	6.61	4.29	4.63	0.67	5.42	-0.45	0.87	0.78	0.99	0.29	0.6	0.09
G111	Brain	5.24	4.79	4.18	3.61	6.68	4.39	4.44	4.92	-0.57	6.83	-0.43	0.84	0.83	0.92	0.71	0.77	0.68
G112	Brain	0.26	1.15	2.82	1.00	1.08	3.23	3.89	4.03	1.09	3.29	-0.98	0.8	0.79	0.92	0.91	1.01	0.94
G118	Brain	3.69	3.58	2.82	2.41	5.84	2.93	3.87	6.75	2.53	5.05	-0.45	0.8	0.79	0.99	0.74	0.83	0.71
G120	Brain	4.52	3.32	4.26	3.32	7.14	2.81	4.51	6.52	2.44	5.84	-0.29	0.86	0.68	0.97	0.73	0.78	0.6
G121	Brain	2.68	3.22	3.44	3.23	7.62	3.13	3.66	7.38	0.48	5.42	-0.59	0.88	0.88	0.98	0.64	0.68	0.69
G122	Brain	-0.41	0.70	2.36	0.80	3.22	2.85	4.48	4.76	0.47	2.99	-1.00	0.82	0.86	0.98	0.88	0.93	0.9
G124	Brain	0.71	2.95	5.78	2.86	6.76	2.21	3.34	5.56	2.51	4.76	-0.72	0.87	0.77	0.96	0.82	0.85	0.68
G130	Brain	2.22	2.42	3.72	2.70	3.36	1.56	3.29	5.50	2.02	5.20	-0.81	0.88	0.9	0.99	0.92	0.96	0.9
G140	Brain	1.79	2.49	2.19	0.88	7.35	3.94	3.35	4.17	1.52	5.01	-0.48	0.78	0.87	0.94	0.7	0.81	0.72
G141	Brain	1.69	3.19	2.35	1.30	5.42	2.41	3.92	6.66	1.41	4.61	-0.54	0.82	0.82	0.97	0.71	0.74	0.64
G142	Brain	3.32	2.25	2.58	1.13	4.11	2.78	2.49	5.99	0.54	3.91	-0.58	0.85	0.81	0.94	0.72	0.82	0.72
G22	Brain	5.11	4.03	3.92	1.93	6.56	3.72	4.29	3.82	-2.82	6.60	-0.52	0.81	0.96	0.95	0.82	0.87	0.82
G28T	Brain	2.83	2.87	3.92	3.24	7.02	3.09	3.99	7.25	1.53	6.02	-0.32	0.87	0.97	1.02	0.94	0.93	0.87
G44	Brain	3.81	2.84	3.94	1.34	8.37	1.79	4.53	4.29	-1.78	7.06	-0.50	0.81	0.72	0.9	0.76	0.84	0.62
G59	Brain	-0.24	-0.01	2.98	0.65	6.76	1.82	3.22	4.78	1.04	6.70	-0.68	0.78	0.86	0.96	0.8	0.9	0.79
G61	Brain	2.25	4.33	2.70	1.35	7.14	3.27	3.15	4.78	-1.33	4.36	-0.54	0.88	0.82	0.89	0.78	0.88	0.71
G84	Brain	-0.39	1.53	2.29	-1.25	7.37	3.53	1.90	7.08	-3.22	3.51	-0.92	0.7	0.9	0.97	0.68	0.75	0.62
G96	Brain	3.21	2.98	4.39	0.69	8.22	1.53	4.62	2.03	-1.40	7.48	-0.48	0.86	0.76	0.92	0.71	0.86	0.69
Hs 683	Brain	3.67	3.98	4.47	4.39	8.39	3.35	4.83	7.20	1.67	6.86	-0.26	0.83	0.83	0.95	0.81	0.88	0.81
IMR-32	Brain	1.75	0.03	0.51	-0.06	7.86	-0.52	1.06	0.14	-3.66	2.87	-1.03	0.98	0.94	0.98	0.83	0.87	0.87
KELLY	Brain	1.72	1.65	3.83	-1.08	6.68	-0.71	3.40	1.74	-2.32	1.57	-0.91	0.93	0.85	0.89	0.68	0.75	0.66
LN-18	Brain	4.44	3.35	2.72	2.96	6.58	3.32	3.73	6.87	-1.01	6.17	-0.58	1.04	0.94	0.98	0.85	0.9	0.88
LN-229	Brain	4.85	5.00	5.56	3.78	7.97	3.72	4.37	6.25	2.43	6.02	-0.31	0.97	0.92	0.88	0.69	0.78	0.69
SF268	Brain	4.22	2.61	1.72	1.87	7.82	3.92	3.51	6.70	1.96	3.13	-0.55	0.88	0.87	0.98	0.82	1.03	0.9
SF539	Brain	-1.31	-0.39	2.14	-0.38	5.39	1.43	3.42	6.34	-0.46	3.07	-0.77	0.75	0.83	1.05	0.68	0.75	0.6
SK-N-AS	Brain	3.55	5.91	6.80	5.44	7.21	6.05	4.02	6.08	-0.17	6.83	-0.13	0.9	0.85	1.03	0.51	0.67	0.32
SK-N-DZ	Brain	-2.05	-3.01	3.02	-5.02	7.65	-1.95	-2.84	-4.82	-8.17	1.85	-1.58	0.94	0.82	1.02	0.56	NaN	0.45
SK-N-FI	Brain	7.60	5.90	5.47	5.05	7.90	4.97	5.53	-1.31	-5.12	4.84	-0.27	0.89	0.84	0.98	0.74	0.8	0.64
SK-N-SH	Brain	4.02	4.27	4.40	4.15	7.81	4.76	4.19	4.67	-5.03	4.01	-0.49	0.88	0.78	0.92	0.77	0.8	0.76
T98G	Brain	3.08	0.38	1.37	-0.14	7.21	1.75	3.14	6.85	4.03	6.12	-0.54	0.82	0.7	0.91	0.74	0.81	0.73
BT-20	Breast	3.67	-0.79	2.65	0.26	6.09	3.03	4.48	3.91	-0.89	4.06	-0.80	0.68	0.89	0.98	0.88	0.88	0.75
BT-474	Breast	6.39	3.89	0.49	1.63	6.48	1.51	0.92	1.71	0.53	-0.18	-0.62	0.78	0.81	1.02	0.98	1	0.96
BT-549	Breast	4.27	4.30	4.15	2.39	3.90	2.60	4.48	6.28	3.19	6.11	-0.43	0.76	0.91	0.98	0.9	0.95	0.9
CAL-120	Breast	0.51	1.76	5.27	1.33	7.38	2.43	3.61	4.65	2.74	5.23	-0.64	0.87	0.78	0.96	0.82	0.82	0.72
CAL-148	Breast	2.02	-0.34	2.51	-0.58	-3.17	-1.11	-7.83	-2.13	-4.46	-0.82	-1.61	0.42	0.94	1.09	0.94	0.99	0.92
CAL51	Breast	1.36	5.75	5.85	4.46	8.08	5.16	4.40	4.91	-7.23	5.04	-0.50	0.67	0.79	0.97	0.82	0.77	0.66
CAMA-1	Breast	-2.51	-0.75	-4.02	3.20	8.01	-2.32	-3.91	2.45	-10.25	-2.28	-1.82	0.68	0.88	0.99	0.96	0.97	0.91
EFM-19	Breast	0.23	1.33	-3.13	0.37	7.82	-1.89	-5.83	3.56	-10.25	1.18	-1.70	0.68	0.66	1	1.01	1.02	0.95
EVSA-T	Breast	2.98	-0.04	-1.77	-0.09	3.69	-0.92	-7.83	-0.24	-3.76	-3.88	-1.60	0.59	0.73	0.94	0.92	0.96	0.95
HCC1143	Breast	0.74	0.43	2.02	0.42	9.07	2.20	0.58	5.19	1.43	4.18	-0.79	0.83	0.89	0.96	0.8	0.8	0.6
HCC1187	Breast	-0.61	-2.48	0.89	-0.69	6.45	0.38	-0.41	0.99	-0.71	1.53	-1.18	0.75	0.89	1.01	0.92	0.94	0.89
HCC1419	Breast	7.61	4.52	1.19	3.38	7.43	1.23	2.80	0.90	-3.46	-0.92	-0.67	0.94	0.93	1	1.01	1	0.99

HCC1806	Breast	3.16	5.29	2.21	2.31	6.60	3.54	2.68	6.03	-1.27	6.38	-0.46	0.85	0.96	0.99	0.68	0.79	0.51
HCC1937	Breast	2.91	4.01	4.17	2.26	7.97	3.77	4.97	6.42	1.41	5.94	-0.33	0.84	0.83	0.99	0.9	0.9	0.8
HCC1954	Breast	4.41	5.11	4.26	2.62	9.75	4.37	4.38	5.60	1.11	4.05	-0.39	0.68	0.9	1.04	0.77	0.75	0.63
HCC38	Breast	2.89	1.79	3.56	1.97	7.69	3.04	4.79	6.23	0.59	5.33	-0.68	0.88	0.93	0.99	0.96	0.97	0.92
Hs 578T	Breast	-1.57	1.85	2.58	1.45	6.49	1.79	4.36	4.63	2.00	4.13	-0.46	0.78	0.89	0.95	0.92	0.96	0.89
JIMT-1	Breast	2.43	2.06	0.36	1.08	7.28	5.17	3.90	4.29	3.22	4.62	-0.38	0.83	0.81	0.94	0.85	0.91	0.76
KPL-1	Breast	6.05	1.92	0.40	2.23	7.32	2.86	3.20	3.31	1.56	2.26	-0.70	0.8	0.69	1.01	1.01	1.1	1.04
MCF 10A	Breast	5.16	4.05	3.89	3.15	5.77	3.61	3.45	6.03	-1.86	3.99	-0.40	0.88	0.83	1.01	0.7	0.71	0.51
MCF-7	Breast	1.35	-2.46	-1.81	-6.38	6.53	-1.19	-3.98	2.97	4.90	2.24	-1.52	0.73	0.63	0.98	0.92	0.93	0.85
MDA-MB-157	Breast	1.72	0.74	1.87	0.83	5.82	0.90	2.31	5.90	1.62	3.63	-0.94	0.92	0.88	0.92	0.84	0.91	0.8
MDA-MB-175	Breast	7.57	6.63	1.38	6.05	8.11	5.59	2.96	2.81	1.17	5.35	-0.29	0.79	0.95	1.01	0.75	0.75	0.67
MDA-MB-231	Breast	6.07	6.44	5.49	5.44	7.85	4.71	4.07	6.74	0.82	7.04	-0.21	0.97	0.92	0.93	0.69	0.74	0.56
MDA-MB-436	Breast	4.44	0.37	4.26	4.18	6.20	4.48	4.04	5.48	1.07	6.89	-0.70	0.85	0.88	1	0.89	0.94	0.81
MDA-MB-453	Breast	3.92	5.69	0.86	3.52	7.06	0.11	1.10	2.15	1.96	-1.98	-0.91	0.73	0.83	0.98	0.95	0.99	0.9
MDA-MB-468	Breast	3.04	1.99	3.81	1.73	6.31	3.10	2.45	3.64	1.66	3.31	-0.64	0.74	0.87	0.93	0.74	0.8	0.68
MFM-223	Breast	4.03	5.20	1.57	2.14	7.79	4.41	3.81	2.37	-1.41	0.97	-0.45	0.68	0.95	1.01	0.86	0.96	0.73
MT-3	Breast	2.23	2.39	3.93	1.68	6.72	5.17	3.35	4.82	-0.84	6.29	-0.75	0.74	0.79	1.02	0.71	0.73	0.5
MX-1	Breast	1.65	2.20	3.01	-2.31	1.65	3.81	2.94	2.86	1.04	-0.80	-0.78	0.85	0.87	0.99	0.94	0.94	0.81
SK-BR-3	Breast	4.45	0.18	1.28	1.99	6.80	2.03	-5.37	3.68	-0.04	0.98	-1.43	0.76	0.77	1.02	0.97	1.04	0.96
SUM 149PT	Breast	3.94	3.41	3.14	2.08	5.62	5.32	3.86	6.08	-3.75	4.49	-0.61	0.73	0.83	0.98	0.74	0.7	0.57
SW 527	Breast	4.25	6.56	5.06	6.14	7.68	6.59	5.20	5.91	3.19	5.67	-0.24	0.85	0.82	0.91	0.77	0.74	0.61
SW 982	Breast	3.61	5.14	3.83	3.63	7.73	4.22	4.47	6.35	1.77	6.45	-0.20	0.92	0.84	0.87	0.73	0.88	0.75
T-47D	Breast	3.49	1.71	1.15	-1.02	6.43	0.83	-1.28	0.24	1.34	3.63	-1.07	0.77	0.74	0.97	0.94	0.95	0.87
YMB-1-E	Breast	5.22	-1.66	-0.78	-1.83	7.45	-1.28	-5.12	1.36	-2.73	-3.92	-1.19	0.61	0.89	1.02	0.95	0.99	0.9
C-33 A	Cervix	-3.35	2.77	-1.05	2.96	5.60	4.49	4.05	2.91	-5.03	0.27	-1.09	0.65	0.92	0.98	0.7	0.76	0.57
Ca Ski	Cervix	4.20	1.80	1.50	-1.88	7.46	2.23	0.73	6.06	2.51	4.48	-0.73	0.77	0.86	0.98	0.8	0.85	0.73
HeLa	Cervix	1.99	-1.15	1.22	-3.78	5.85	4.35	0.32	5.69	-1.28	1.08	-1.02	0.95	0.86	0.99	0.9	0.91	0.77
HEp-2	Cervix	-0.73	-0.86	1.58	-4.11	5.50	0.63	1.08	5.31	-7.27	1.08	-1.26	0.91	0.87	1	0.86	0.89	0.82
ME-180	Cervix	3.35	5.26	0.28	0.17	7.17	3.48	0.83	6.36	0.84	5.75	-0.67	0.47	0.69	0.98	0.72	0.76	0.58
MS751	Cervix	3.55	-1.67	0.45	-4.11	5.49	-0.02	-1.58	5.90	-8.03	3.27	-1.49	0.95	0.9	0.95	0.87	0.92	0.81
SiHa	Cervix	3.51	0.99	2.99	1.52	4.62	3.44	4.24	6.96	0.81	4.36	-0.63	0.87	0.9	0.96	0.87	0.89	0.83
ATRFLOX	Colo-Rectal	4.83	6.02	1.90	4.94	7.35	4.95	5.29	6.01	-2.04	7.12	-0.24	0.84	0.83	0.93	0.51	0.53	0.27
C170	Colo-Rectal	2.12	5.40	3.57	2.05	7.36	4.71	3.47	6.37	-10.25	6.36	-0.75	0.89	0.77	0.97	0.62	0.67	0.41
C2BBe1	Colo-Rectal	0.25	3.60	4.04	2.62	7.06	4.88	1.97	5.51	3.28	4.11	-0.55	0.91	0.91	0.97	0.87	0.87	0.76
Caco-2	Colo-Rectal	0.62	4.63	4.68	3.25	6.92	5.74	1.31	5.15	2.33	4.15	-0.71	0.91	0.88	0.98	0.77	0.82	0.7
CaR-1	Colo-Rectal	3.78	3.17	3.94	0.79	8.38	4.22	1.89	6.41	-1.16	5.68	-0.62	0.83	0.77	0.96	0.74	0.8	0.57
CL-11	Colo-Rectal	0.86	6.23	5.74	4.03	7.18	4.39	4.93	6.10	0.30	7.82	-0.46	0.93	0.82	1.04	0.53	0.68	0.34
CL-34	Colo-Rectal	1.31	5.71	4.71	2.09	5.38	4.19	2.71	4.04	-1.78	5.80	-0.67	0.94	0.98	0.92	0.62	0.73	0.45
CoCM-1	Colo-Rectal	-0.14	5.42	4.42	2.48	5.76	4.92	0.40	5.21	-1.54	4.31	-1.12	0.98	0.81	1	0.7	0.77	0.62
COLO 201	Colo-Rectal	4.76	5.78	4.03	3.68	5.36	4.51	3.67	4.43	3.14	7.31	-0.36	0.92	0.94	0.67	0.28	0.54	0.15
COLO 205	Colo-Rectal	3.84	5.76	4.88	4.01	4.03	4.34	3.45	2.40	1.99	6.23	-0.58	0.79	0.87	0.69	0.36	0.66	0.24
COLO 206F	Colo-Rectal	4.31	6.10	4.27	3.99	4.61	4.40	3.27	4.12	1.49	6.73	-0.47	0.84	0.86	0.69	0.29	0.57	0.16
COLO 320DM	Colo-Rectal	-7.54	-0.39	-2.05	-5.76	3.11	2.10	3.85	3.57	2.64	0.29	-1.05	0.95	0.92	0.98	0.93	1.05	0.98

COLO 320HSR	Colo-Rectal	-6.55	1.59	3.50	-3.37	5.17	3.33	3.49	3.16	1.61	-0.41	-1.09	0.96	0.84	0.99	0.96	1.04	0.98
COLO 678	Colo-Rectal	2.59	5.46	2.49	1.62	8.24	5.77	3.66	7.38	-2.63	5.53	-0.66	0.91	0.93	1.01	0.73	0.76	0.59
COLO 741	Colo-Rectal	6.24	4.98	6.10	3.16	6.33	5.05	5.97	3.35	2.14	7.24	-0.43	0.94	0.91	0.86	0.67	0.74	0.52
CW-2	Colo-Rectal	-3.28	4.23	4.70	2.85	5.58	4.70	5.00	4.08	-5.05	3.56	-1.04	0.99	0.87	1.01	0.84	0.85	0.66
CX-1	Colo-Rectal	2.17	6.41	3.99	4.23	5.18	5.98	4.28	6.83	-4.39	5.93	-0.73	0.91	0.82	0.85	0.51	0.61	0.45
DLD-1	Colo-Rectal	0.03	6.29	3.45	2.10	7.73	4.29	3.12	5.20	-0.46	5.47	-0.67	0.95	0.89	0.94	0.75	0.78	0.65
GP2d	Colo-Rectal	1.69	4.42	3.42	4.76	6.24	5.83	4.34	3.97	-8.25	2.97	-0.74	0.7	0.69	1.02	0.73	0.82	0.62
GP5d	Colo-Rectal	2.36	3.91	3.04	3.52	6.41	5.61	4.19	5.33	-10.25	4.22	-0.83	0.79	0.74	1.06	0.79	0.93	0.72
HCA-7	Colo-Rectal	2.26	5.79	2.68	2.05	8.01	4.87	3.70	6.16	-1.26	4.89	-0.62	0.87	0.8	1.01	0.69	0.65	0.47
HCC1263	Colo-Rectal	0.94	3.58	3.76	2.74	5.74	5.09	-1.68	4.54	-3.60	3.78	-0.87	0.86	1.03	0.98	0.75	0.78	0.63
HCT 116	Colo-Rectal	5.10	7.16	2.10	4.48	7.26	4.57	5.57	5.77	-0.04	7.10	-0.21	0.92	0.89	0.96	0.89	0.77	0.7
HCT-15	Colo-Rectal	-1.29	3.67	3.00	1.96	6.86	4.25	4.58	5.57	-5.89	6.13	-0.95	0.91	0.86	0.96	0.8	0.81	0.64
HCT-8	Colo-Rectal	0.09	5.88	4.27	2.58	7.15	4.04	4.14	5.02	-5.07	3.58	-0.81	0.87	0.81	0.91	0.64	0.71	0.5
HM7	Colo-Rectal	3.70	6.39	4.52	2.57	6.86	6.98	5.64	7.06	1.52	6.39	-0.27	0.7	0.81	1.02	0.59	0.73	0.38
HT-115	Colo-Rectal	3.33	5.99	3.80	3.09	6.79	5.73	2.81	6.19	-5.47	6.03	-0.63	0.89	0.81	1.05	0.68	0.71	0.48
HT-29	Colo-Rectal	1.69	5.04	4.89	3.40	5.45	5.69	4.36	5.39	-3.21	3.99	-0.58	0.86	0.79	0.75	0.37	0.66	0.31
HT-55	Colo-Rectal	-1.39	4.77	4.64	4.01	7.01	6.19	-0.11	5.36	1.62	5.88	-0.54	0.83	0.75	0.95	0.64	0.77	0.49
KM-12	Colo-Rectal	5.76	6.11	5.37	3.24	5.83	5.02	4.66	5.41	1.00	6.24	-0.20	0.93	0.99	0.97	0.86	0.88	0.79
LoVo	Colo-Rectal	4.07	4.45	4.57	2.64	6.66	4.77	3.38	6.23	-0.23	5.30	-0.53	0.77	0.79	0.98	0.61	0.72	0.44
LS1034	Colo-Rectal	-1.20	4.18	4.70	3.80	6.43	6.30	-0.28	4.69	-1.49	6.50	-0.61	0.94	0.95	1.02	0.67	0.7	0.39
MDST8	Colo-Rectal	4.49	4.47	5.73	2.19	8.73	4.13	4.70	5.00	2.62	7.94	-0.47	0.79	0.9	0.91	0.72	0.7	0.43
NCI-H716	Colo-Rectal	3.84	5.53	4.84	5.06	7.05	5.42	5.26	2.50	4.69	2.01	-0.50	0.88	0.84	1	0.7	0.67	0.46
OUMS-23	Colo-Rectal	2.46	5.42	4.37	5.09	7.77	5.77	3.35	5.41	2.47	4.00	-0.05	0.83	0.92	0.92	0.83	0.86	0.87
RCM-1	Colo-Rectal	2.37	6.55	3.92	4.27	6.64	6.15	4.52	5.61	-7.31	6.30	-0.45	0.99	0.91	1.03	0.73	0.75	0.49
RKO	Colo-Rectal	-2.75	5.52	3.42	6.66	5.51	4.89	4.51	6.12	-7.08	6.43	-0.65	0.79	0.89	0.85	0.48	0.66	0.36
RKO-E6	Colo-Rectal	-0.39	4.47	3.39	6.42	5.84	4.99	4.53	6.66	-8.04	6.42	-0.70	0.75	0.87	0.86	0.57	0.73	0.41
RPMI 2650	Colo-Rectal	0.55	-1.68	-3.68	0.51	7.95	3.88	2.07	5.36	-4.32	0.86	-0.91	0.81	0.77	1.01	0.99	1.06	0.92
SK-CO-1	Colo-Rectal	6.29	5.46	4.65	3.25	6.95	4.98	2.31	7.42	-10.25	6.52	-0.78	0.92	0.81	0.95	0.49	0.61	0.26
SNU-C1	Colo-Rectal	0.36	4.62	4.62	2.31	5.58	4.91	0.05	4.82	-2.08	3.74	-0.78	0.75	0.92	1.02	0.73	0.81	0.5
SW 1463	Colo-Rectal	-0.04	6.22	5.79	3.81	4.11	6.76	3.34	5.26	-2.96	4.60	-0.39	1.03	1	1.03	0.63	0.69	0.59
SW 403	Colo-Rectal	-2.54	4.32	4.43	3.36	5.09	4.98	1.77	3.84	-10.25	3.63	-1.32	0.82	0.86	1.06	0.68	0.73	0.42
SW 48	Colo-Rectal	5.37	6.13	4.68	4.31	7.02	5.19	4.10	6.05	0.81	6.65	-0.46	0.79	0.72	1.05	0.62	0.66	0.31
SW 480	Colo-Rectal	1.74	7.38	3.08	4.12	9.58	6.29	5.85	5.30	3.08	4.44	-0.33	0.92	0.91	1.08	0.63	0.67	0.41
SW 620	Colo-Rectal	3.85	5.95	5.60	2.58	6.43	5.55	5.82	5.70	2.56	7.07	-0.40	0.96	0.9	1.09	0.63	0.68	0.36
SW 837	Colo-Rectal	0.92	3.52	1.04	3.62	7.23	5.36	3.13	6.97	-1.86	4.52	-0.63	0.91	0.87	1.03	0.7	0.72	0.43
SW 948	Colo-Rectal	2.63	5.76	4.83	2.39	5.35	4.60	3.33	4.63	-4.93	6.69	-0.53	0.79	0.81	0.98	0.65	0.73	0.5
T84	Colo-Rectal	0.00	7.17	3.51	3.69	5.33	6.35	3.17	5.22	-6.76	6.60	-0.68	0.95	0.99	0.98	0.75	0.7	0.59
WiDr	Colo-Rectal	3.54	4.76	3.61	4.14	5.47	5.50	4.07	5.94	-6.37	4.98	-0.55	0.79	0.72	0.75	0.38	0.58	0.31
BICR 22	Head-Neck	0.89	4.74	1.39	2.23	7.60	3.72	1.24	6.11	-3.57	5.88	-0.55	0.77	0.86	0.97	0.81	0.82	0.63
CAL 27	Head-Neck	1.83	3.28	0.80	-2.07	6.57	2.51	0.08	6.14	-3.69	4.33	-0.70	0.81	0.91	0.96	0.62	0.66	0.34
COLO 680N	Head-Neck	3.40	5.25	1.36	2.32	8.12	3.87	2.09	6.12	0.43	6.02	-0.42	0.92	0.89	0.99	0.79	0.97	0.83
KYSE-140	Head-Neck	1.07	5.10	4.34	2.43	7.04	5.41	4.31	4.85	1.61	5.34	-0.76	0.65	0.84	1.01	0.74	0.77	0.71
KYSE-150	Head-Neck	3.16	2.19	1.80	0.62	7.25	3.91	1.95	5.05	-6.24	4.97	-0.68	0.83	0.87	1	0.69	0.75	0.63
KYSE-180	Head-Neck	-1.20	2.44	2.30	0.62	7.58	3.51	1.22	4.80	1.71	2.92	-0.78	0.67	0.84	1	0.84	0.92	0.93
KYSE-270	Head-Neck	3.83	2.07	2.45	0.28	6.88	4.61	3.49	5.54	1.31	2.86	-0.55	0.86	0.78	1.01	0.76	0.84	0.68
KYSE-30	Head-Neck	4.92	2.68	4.07	3.07	7.33	4.14	4.09	6.63	1.49	5.50	-0.38	0.8	0.92	0.94	0.61	0.6	0.45
KYSE-450	Head-Neck	-1.49	1.32	1.40	-1.16	5.80	3.25	1.89	1.96	-1.23	4.80	-0.86	0.75	0.67	1.03	0.66	0.7	0.52

KYSE-510	Head-Neck	2.33	0.42	0.21	-2.05	9.03	2.89	-0.17	4.86	2.16	3.22	-0.90	0.87	0.82	0.98	0.75	0.89	0.79
KYSE-520	Head-Neck	3.97	3.39	3.68	1.91	7.77	3.27	3.04	6.03	1.25	5.28	-0.41	0.82	0.81	0.97	0.67	0.72	0.57
KYSE-70	Head-Neck	0.74	1.80	2.23	1.61	5.89	5.44	5.96	5.70	-1.03	6.40	-0.53	0.72	0.76	1	0.77	0.88	0.74
PE/CA-PJ15	Head-Neck	4.33	2.64	2.57	0.27	8.25	3.44	2.69	6.01	2.13	4.73	-0.46	0.88	0.84	0.95	0.66	0.69	0.49
769-P	Kidney	-0.42	2.81	2.65	1.01	7.36	2.62	3.82	5.33	1.53	5.00	-0.62	0.81	0.81	0.95	0.72	0.8	0.59
786-O	Kidney	-3.06	0.00	1.67	-3.46	6.92	0.01	2.30	6.14	1.61	3.48	-1.23	0.83	0.87	0.9	0.89	0.91	0.82
A-498	Kidney	-1.48	-1.02	0.02	-0.50	4.82	0.83	2.43	5.12	0.02	3.69	-0.97	0.7	0.85	0.98	0.78	0.84	0.72
ACHN	Kidney	-3.93	1.78	2.18	-1.15	6.50	2.66	3.21	6.03	0.74	4.76	-0.98	0.88	0.77	0.94	0.81	0.83	0.69
BFTC-909	Kidney	0.44	-0.99	2.12	1.51	4.95	2.98	3.74	5.90	-3.49	3.74	-0.69	0.7	0.7	0.96	0.8	0.87	0.77
Caki-1	Kidney	0.84	4.16	2.97	1.31	5.78	1.93	4.49	5.46	1.20	5.79	-0.41	0.82	0.77	0.97	0.89	0.8	0.7
Caki-2	Kidney	4.22	2.08	1.87	1.08	6.63	4.94	4.67	5.24	1.77	5.49	-0.42	0.86	0.88	0.98	0.92	0.72	0.59
CAL 54	Kidney	-1.82	3.18	3.79	2.09	7.64	3.48	3.88	6.52	-0.37	6.43	-0.69	0.83	0.74	0.91	0.91	0.85	0.71
G-402	Kidney	-2.93	-4.41	1.48	3.46	6.46	1.69	2.46	5.61	-8.16	6.19	-0.84	0.72	0.63	0.96	0.72	0.84	0.69
HCC12	Kidney	6.14	6.95	3.30	5.04	-0.75	5.48	5.38	2.71	0.41	4.54	-0.42	NaN	NaN	NaN	NaN	NaN	NaN
HCC89	Kidney	1.32	0.26	1.37	-2.46	8.40	-2.75	2.27	5.32	1.43	6.43	-0.63	0.9	0.91	0.96	0.96	0.95	0.91
TK-10	Kidney	-2.90	0.75	2.76	1.39	6.59	2.85	3.16	5.25	-2.51	4.82	-0.91	0.83	0.84	0.9	0.8	0.75	0.65
C3A	Liver	-1.49	5.26	2.17	4.29	6.57	7.10	4.65	6.27	-10.25	5.28	-0.58	0.94	0.91	0.93	0.79	0.76	0.59
Hep G2	Liver	-0.40	5.18	2.74	4.67	7.72	6.89	5.01	5.56	-6.45	5.88	-0.48	0.91	0.82	0.94	0.51	0.65	0.36
huH-1	Liver	0.81	3.06	1.89	0.68	5.65	1.49	2.10	3.95	-2.20	5.02	-0.69	0.87	0.75	0.91	0.82	0.82	0.7
HUH-7	Liver	3.28	5.04	4.60	4.17	6.48	5.43	4.50	5.73	-10.25	4.24	-0.41	0.82	0.85	0.89	0.78	0.72	0.68
JHH-1	Liver	-3.42	1.29	1.59	2.04	7.62	2.81	0.64	6.29	-5.87	2.78	-1.35	0.9	0.73	0.95	0.58	0.6	0.4
JHH-2	Liver	-4.73	0.86	3.66	1.71	6.32	2.32	2.65	4.62	1.41	3.35	-0.82	0.87	0.9	1.01	0.74	0.81	0.74
JHH-4	Liver	4.11	4.20	4.06	2.22	7.35	4.40	3.93	6.00	0.64	7.19	-0.44	0.89	0.79	0.93	0.73	0.68	0.59
JHH-5	Liver	3.25	5.49	4.41	4.71	6.99	5.83	3.86	4.82	-10.25	3.70	-0.59	0.71	0.71	1.04	0.5	0.55	0.26
JHH-7	Liver	1.52	5.06	4.46	3.45	7.16	7.13	4.42	5.23	-7.09	2.68	-0.59	0.94	0.95	0.96	0.76	0.84	0.72
PLC/PRF/5	Liver	5.07	4.31	4.29	4.52	6.88	6.16	3.72	5.22	-4.40	4.27	-0.42	0.96	0.92	0.94	0.94	0.92	0.85
SNU-182	Liver	1.53	2.30	4.73	3.10	7.04	3.90	3.17	5.74	1.24	1.98	-0.51	0.86	0.83	1	0.77	0.77	0.62
SNU-387	Liver	1.29	1.89	5.28	1.85	8.57	2.63	4.09	5.85	0.54	3.77	-0.45	0.99	1.04	0.96	0.78	0.76	0.71
SNU-398	Liver	0.11	7.18	6.45	4.27	6.12	4.81	4.60	4.42	-3.89	4.51	-0.79	0.95	1.01	0.95	0.72	0.76	0.63
SNU-423	Liver	2.01	1.65	3.51	0.79	6.24	3.51	2.63	6.23	2.08	5.25	-0.54	0.8	0.79	0.9	0.89	0.88	0.8
SNU-449	Liver	3.31	2.37	4.42	2.77	7.52	5.00	3.55	6.85	-4.42	5.35	-0.38	0.96	0.9	NaN	0.68	0.84	0.78
SNU-475	Liver	1.98	-2.05	0.34	0.68	6.47	0.69	2.26	5.44	-3.38	1.38	-1.06	0.83	0.83	0.94	0.94	0.91	0.89
A549	Lung	5.24	2.10	2.55	1.38	5.83	4.89	3.73	4.77	-2.46	4.25	-0.53	0.81	0.8	0.95	0.54	0.64	0.36
ABC-1	Lung	2.83	0.40	3.02	-0.91	6.33	3.12	1.90	5.58	1.63	5.61	-0.81	0.86	0.87	0.99	0.84	0.86	0.74
BEN	Lung	5.94	5.79	2.36	2.70	2.87	5.62	3.99	1.13	0.34	3.03	-0.57	0.99	1	0.99	0.92	0.94	0.87
CAL-12T	Lung	5.61	4.93	2.97	2.59	7.53	5.55	2.00	6.38	-4.60	5.63	-0.42	0.79	0.85	0.85	0.63	0.77	0.55
Calu-3	Lung	4.85	5.29	3.56	2.61	7.43	4.56	4.55	6.03	-1.50	6.19	-0.27	0.79	0.79	0.91	0.81	0.82	0.75
ChaGo-K-1	Lung	2.46	4.48	1.57	1.33	5.29	0.73	2.98	6.43	-1.05	3.51	-0.63	0.97	0.97	0.96	0.92	1	0.92
COR-L26	Lung	2.29	-0.70	2.98	-2.23	0.56	-0.03	0.29	-3.81	2.96	0.23	-1.19	0.85	0.88	1.02	0.75	0.93	0.7
COR-L279	Lung	2.86	1.28	-0.36	-2.86	-0.48	0.61	-1.74	-0.99	3.28	4.65	-2.17	0.81	0.81	0.99	0.98	1.03	0.97
COR-L47	Lung	0.53	-0.03	1.22	-3.51	-1.41	-1.57	-3.60	-1.90	0.72	1.02	-1.60	0.91	0.92	1.01	1	1.02	0.95
COR-L88	Lung	2.27	1.49	3.09	-2.74	-1.98	-1.47	-2.93	-3.32	2.85	-0.06	-1.25	0.97	1.02	1.01	0.92	0.99	0.92
DMS 273	Lung	-1.72	1.42	2.15	1.23	4.91	1.74	3.53	2.71	1.80	2.63	-1.31	0.66	0.94	0.95	0.9	0.98	0.99
DV-90	Lung	6.22	2.78	3.30	2.96	7.04	5.04	4.09	7.21	0.36	2.09	-0.46	0.8	0.92	0.98	0.62	0.71	0.42
EPLC-272H	Lung	4.30	4.42	3.15	1.90	9.33	4.34	2.34	5.67	0.94	5.63	-0.40	0.78	0.78	0.94	0.78	0.89	0.73
H322T	Lung	3.73	3.28	2.53	2.09	7.11	2.79	4.41	4.93	-1.30	5.02	-0.84	0.9	0.86	0.97	0.72	0.73	0.56
HCC-15	Lung	5.57	4.57	3.61	4.20	4.48	4.86	4.24	6.34	-1.37	6.73	-0.55	0.89	0.93	0.96	0.6	0.72	0.46
HCC1171	Lung	3.18	7.37	5.02	5.73	7.40	5.30	4.12	6.51	1.62	6.93	-0.21	0.95	0.95	0.94	0.68	0.74	0.55

HCC2279	Lung	1.17	3.60	3.11	1.43	5.62	0.97	3.89	6.04	1.11	4.40	-0.73	0.87	0.88	0.96	0.84	0.9	0.85
HCC2302	Lung	5.61	2.40	2.63	2.57	6.90	4.13	2.73	6.52	-1.86	4.37	-0.67	0.93	0.93	1	0.8	0.87	0.72
HCC2885	Lung	2.50	5.11	2.09	2.84	8.25	3.70	3.89	6.46	1.22	7.27	-0.29	0.95	0.94	1	0.83	0.85	0.72
HCC364	Lung	5.85	5.95	3.68	2.51	5.56	1.61	3.87	6.46	1.40	6.89	-0.24	0.92	0.93	0.81	0.38	0.6	0.15
HCC4006	Lung	4.03	4.38	3.06	2.22	7.95	3.37	3.41	6.15	2.32	4.83	-0.40	0.89	0.87	0.93	0.78	0.71	0.71
HCC4011	Lung	2.23	5.61	2.29	2.12	7.11	4.88	4.41	6.16	0.17	5.86	-0.52	0.9	0.92	0.97	0.84	0.81	0.75
HCC4017	Lung	5.10	5.32	4.26	4.23	6.68	4.89	3.78	7.10	0.78	7.09	-0.40	0.91	0.89	0.95	0.71	0.78	0.58
HCC44	Lung	3.96	4.05	4.99	2.40	6.89	3.60	4.11	6.48	-4.66	6.30	-0.67	0.74	0.76	0.97	0.61	0.64	0.39
HCC461	Lung	5.19	3.82	3.98	3.14	7.49	4.15	3.95	6.60	1.65	6.34	-0.24	0.83	0.83	0.96	0.74	0.83	0.63
HCC515	Lung	4.44	3.62	3.51	2.88	8.87	3.16	4.25	6.84	-5.60	6.91	-0.49	0.93	0.93	0.94	0.7	0.74	0.59
HCC78	Lung	-0.49	2.30	2.60	0.38	7.33	2.83	3.66	5.64	-0.90	4.12	-0.69	0.82	0.77	0.96	0.79	0.85	0.76
HCC827	Lung	2.03	3.51	3.21	0.57	7.08	3.41	3.35	5.27	-0.68	5.13	-0.45	0.75	0.81	0.96	0.71	0.69	0.58
HOP-62	Lung	2.72	4.20	3.81	3.19	7.94	4.59	4.37	7.42	1.28	6.00	-0.39	0.98	0.91	0.87	0.76	0.72	0.7
HOP-92	Lung	1.59	4.94	4.49	3.38	7.15	4.15	4.25	5.66	-0.43	5.29	-0.68	0.92	0.86	0.99	0.63	0.62	0.52
HT-1080	Lung	4.93	-2.12	4.40	3.28	7.37	4.55	4.68	6.89	0.85	7.22	-0.39	0.89	0.94	0.92	0.73	0.82	0.57
KNS-62	Lung	-1.01	7.07	2.93	2.70	5.88	4.91	4.76	5.01	-0.73	6.56	-0.67	0.68	0.87	0.98	0.64	0.73	0.48
LCLC-103H	Lung	1.25	0.26	1.13	-1.48	4.98	2.63	2.23	5.49	-1.91	4.19	-0.86	0.9	0.88	0.94	0.83	0.94	0.89
LCLC-97TM1	Lung	2.14	6.00	3.16	2.52	7.17	4.34	3.94	5.88	-1.01	6.71	-0.35	0.86	0.88	0.99	0.7	0.79	0.48
LXF-289	Lung	4.40	-0.26	2.77	-1.12	6.51	3.91	3.97	5.35	-0.10	4.19	-0.62	0.82	0.83	0.96	0.69	0.76	0.59
LXFL529	Lung	1.28	7.76	5.86	5.91	5.44	6.04	4.79	4.31	-6.61	4.86	-0.39	0.7	0.7	1.09	0.86	0.82	0.7
MOR	Lung	5.62	5.20	3.30	4.20	6.97	7.51	4.84	4.64	0.55	5.70	-0.30	0.94	0.94	0.96	0.88	0.88	0.83
NCI-H1155	Lung	5.27	5.31	2.64	4.77	-0.82	4.68	4.33	1.61	1.26	4.59	-0.74	0.85	1.01	0.98	1.01	1.04	1.11
NCI-H1299	Lung	-6.02	4.22	4.45	4.67	6.90	3.90	4.53	6.19	-2.87	6.11	-0.68	0.88	0.86	0.98	0.65	0.69	0.53
NCI-H1355	Lung	5.54	1.51	4.10	0.67	5.22	4.24	3.62	5.73	-3.65	4.77	-0.76	0.93	0.89	0.92	0.74	0.76	0.66
NCI-H1437	Lung	5.94	4.04	1.59	2.44	6.20	4.79	3.58	2.82	-2.86	4.52	-0.70	0.86	0.83	0.94	0.53	0.74	0.4
NCI-H146	Lung	4.39	3.32	1.72	-0.19	-2.11	3.27	2.30	0.08	0.61	1.54	-0.99	0.96	0.99	0.98	0.91	0.97	0.94
NCI-H1581	Lung	-4.26	8.32	6.02	2.31	6.14	6.69	4.99	4.23	-6.07	2.37	-0.84	0.98	0.99	1	0.74	0.87	0.71
NCI-H1651	Lung	-0.89	4.15	3.27	0.80	5.35	4.08	3.76	5.52	0.05	3.52	-0.66	0.99	0.9	0.96	0.89	0.92	0.79
NCI-H1666	Lung	6.22	5.16	2.11	1.31	5.67	3.71	1.85	6.62	-0.88	7.59	-0.72	0.83	0.77	0.99	0.64	0.75	0.54
NCI-H1703	Lung	-3.96	1.00	0.45	4.21	4.57	2.74	3.66	5.70	-2.62	4.61	-1.09	0.79	0.69	0.92	0.87	0.95	0.92
NCI-H1838	Lung	2.84	6.31	4.72	2.36	7.39	4.42	4.08	6.17	2.72	6.64	-0.47	0.86	0.86	0.89	0.86	0.89	0.94
NCI-H1869	Lung	1.91	3.91	4.58	2.27	7.69	0.94	3.93	6.22	2.64	5.37	-0.59	0.95	0.89	0.95	0.75	0.83	0.72
NCI-H187	Lung	-0.12	0.17	-6.65	-4.08	-1.63	-1.41	-0.31	-2.79	-1.14	0.82	-2.30	0.74	0.86	1.02	1.03	0.99	0.93
NCI-H1930	Lung	2.42	2.90	3.45	0.75	-2.99	3.13	3.20	-0.29	3.17	1.90	-1.31	1.08	0.83	1.03	1.08	1.01	0.99
NCI-H1944	Lung	4.27	2.88	1.87	1.30	5.08	3.56	3.92	3.85	-7.64	2.02	-0.75	0.85	0.85	0.93	0.72	0.78	0.6
NCI-H196	Lung	-0.77	3.05	3.32	2.66	-1.72	3.68	4.33	5.08	-0.31	3.51	-1.10	0.89	0.9	1.03	0.92	0.94	0.94
NCI-H2009	Lung	3.01	3.90	2.41	0.98	5.60	2.41	3.18	6.19	0.65	6.34	-0.51	0.89	0.87	0.87	0.58	0.68	0.44
NCI-H2023	Lung	4.33	1.42	0.89	2.54	5.65	4.14	4.10	6.63	-3.82	4.42	-0.46	0.77	0.79	0.97	0.74	0.86	0.76
NCI-H2106	Lung	-1.64	-1.04	-2.08	-0.84	-4.02	0.34	4.20	0.92	-1.00	-0.83	-1.58	0.83	0.84	1	0.99	1.02	0.91
NCI-H2110	Lung	6.44	4.90	4.72	1.56	7.16	5.15	3.60	6.36	-5.65	4.32	-0.61	0.93	0.97	0.98	0.89	0.91	0.81
NCI-H2122	Lung	6.50	4.26	2.92	3.04	7.34	3.38	1.78	6.61	0.04	6.07	-0.37	0.93	0.94	0.96	0.64	0.6	0.34
NCI-H2135	Lung	-6.99	5.32	4.74	6.35	6.46	5.22	3.91	6.33	-3.34	8.43	-0.63	0.97	0.97	0.99	0.59	0.69	0.32
NCI-H2170	Lung	5.17	1.77	3.10	1.20	6.48	4.28	2.33	4.75	-0.66	0.88	-0.81	0.94	0.91	0.96	1.05	1.02	1.04
NCI-H2171	Lung	1.08	-3.78	-2.69	-3.22	3.14	-0.91	-1.43	-1.58	3.69	5.42	-1.70	0.86	0.81	1.01	0.95	1.05	0.96
NCI-H2228	Lung	4.66	4.03	3.60	1.89	6.35	2.97	5.23	5.64	0.08	6.24	-0.34	0.81	0.81	0.95	0.84	0.77	0.76
NCI-H226	Lung	0.21	-1.42	1.04	1.52	6.56	0.95	2.29	6.28	3.49	2.84	-0.58	0.96	0.88	1	0.76	0.81	0.65
NCI-H23	Lung	2.40	4.92	3.44	4.81	7.11	4.98	5.38	5.14	-6.37	4.93	-0.64	0.91	0.9	0.95	0.56	0.61	0.47
NCI-H2347	Lung	5.23	6.95	4.34	3.61	8.40	5.68	3.80	6.72	1.98	6.60	-0.17	0.91	0.86	0.94	0.63	0.72	0.52

NCI-H2369	Lung	1.83	2.66	2.76	2.49	6.85	2.23	3.50	6.18	2.80	4.61	-0.47	0.92	0.94	0.88	0.86	0.91	0.83
NCI-H2373	Lung	-3.54	1.20	2.64	1.82	7.37	0.62	3.14	5.63	3.01	4.81	-0.62	0.77	0.85	0.97	0.76	0.84	0.68
NCI-H2452	Lung	-0.70	1.11	1.35	-0.39	6.61	0.20	2.59	5.63	-0.32	4.45	-0.86	0.87	0.84	1.05	0.83	0.86	0.74
NCI-H2461	Lung	1.34	1.53	1.82	1.95	6.09	2.21	3.21	5.49	3.61	4.24	-0.53	0.77	0.79	0.96	0.78	0.84	0.68
NCI-H2595	Lung	0.56	1.44	2.34	2.97	6.22	3.96	4.43	7.44	1.12	3.56	-0.55	0.87	0.89	0.94	0.8	0.85	0.73
NCI-H2722	Lung	1.61	2.67	1.92	2.28	7.88	3.49	3.80	5.79	1.87	4.62	-0.37	0.93	0.96	0.94	0.84	0.85	0.78
NCI-H2803	Lung	1.41	0.62	2.20	-0.17	8.11	0.64	2.65	7.31	0.94	2.23	-0.71	0.97	1	0.97	0.78	0.84	0.71
NCI-H2804	Lung	2.46	3.02	1.41	2.51	6.53	3.94	4.66	6.50	1.67	4.76	-0.39	0.92	0.92	0.94	0.87	0.9	0.88
NCI-H2810	Lung	0.12	4.00	2.99	3.57	6.95	4.32	4.73	5.49	2.04	5.41	-0.49	0.85	0.76	1.02	0.63	0.71	0.58
NCI-H292	Lung	3.41	1.30	2.43	-2.27	5.93	0.34	0.11	4.39	2.08	5.01	-0.75	0.89	0.78	0.98	0.5	0.64	0.36
NCI-H358	Lung	4.80	5.19	3.73	2.46	7.85	4.43	3.83	7.08	1.58	7.01	-0.30	0.87	0.89	0.91	0.66	0.65	0.48
NCI-H441	Lung	-0.44	5.74	3.17	1.54	6.72	3.97	4.05	5.94	0.98	5.48	-0.42	0.92	0.85	0.99	0.79	0.84	0.74
NCI-H446	Lung	1.94	2.44	1.10	0.54	3.30	1.53	3.38	1.39	3.92	4.98	-1.02	0.68	0.76	0.94	0.87	0.87	0.87
NCI-H460	Lung	6.00	5.47	3.06	3.51	5.26	6.79	4.22	4.61	3.71	4.70	-0.34	0.82	0.87	0.92	0.72	0.8	0.6
NCI-H520	Lung	1.23	5.40	4.06	5.11	6.27	5.09	6.22	2.45	-0.18	6.22	-0.32	0.72	0.89	0.9	0.86	0.85	0.82
NCI-H522	Lung	-3.11	5.76	-3.16	2.72	4.37	5.78	3.90	5.64	-2.38	-2.31	-1.06	0.94	0.89	0.96	0.82	0.86	0.81
NCI-H650	Lung	1.61	5.83	5.33	6.28	7.34	5.84	3.44	5.54	0.60	6.80	-0.31	0.91	0.97	0.95	0.74	0.73	0.57
NCI-H727	Lung	6.18	7.43	4.58	4.42	8.15	5.57	3.09	5.86	3.73	4.99	-0.11	0.83	0.94	1.02	0.48	0.63	0.26
NCI-H82	Lung	0.77	-3.36	-2.09	-6.75	-3.01	-3.59	-1.80	1.08	1.67	0.69	-1.42	0.95	0.9	1	0.94	1.02	0.89
NCI-H889	Lung	3.29	2.84	0.91	1.61	0.04	0.36	-0.43	-1.91	0.12	1.89	-1.07	0.97	0.96	0.97	0.93	1.01	0.95
NCI-N417	Lung	-4.16	-1.97	1.43	-0.52	4.18	-1.24	2.05	3.56	0.06	4.32	-0.88	0.71	0.72	0.96	1.03	1.01	0.95
RERF-LC-KJ	Lung	5.24	3.21	2.93	1.89	7.37	1.50	-0.60	5.32	-3.81	4.66	-0.63	0.86	0.84	1.03	0.77	0.8	0.74
RERF-LC-MS	Lung	4.47	3.97	3.41	4.45	5.80	4.90	5.01	5.07	1.80	7.12	-0.33	0.89	0.84	0.97	0.66	0.69	0.53
RERF-LC-OK	Lung	-2.47	0.73	1.44	0.35	6.32	1.78	2.59	4.88	0.01	4.35	-1.07	0.76	0.7	0.95	0.83	0.85	0.75
SCLC-21H	Lung	-2.01	-0.71	-0.30	-2.27	2.27	-3.66	-0.11	-2.02	2.78	1.10	-2.13	0.91	0.89	0.96	0.91	1.02	1.04
SCLC-22H	Lung	-1.77	-1.50	-2.67	-2.01	3.66	-3.19	0.42	-2.75	1.30	-1.01	-2.56	0.91	0.89	1	0.93	0.98	0.94
SK-MES-1	Lung	3.69	2.54	4.18	2.26	7.88	3.59	4.48	7.12	1.22	5.95	-0.37	0.93	0.88	0.92	0.68	0.69	0.56
SW 1573	Lung	-0.75	5.52	5.04	5.39	6.01	5.15	4.42	6.21	3.98	7.49	-0.18	0.81	0.79	0.94	0.81	0.82	0.76
UMC-11	Lung	5.45	3.77	1.83	1.83	0.02	4.94	4.81	-3.44	2.71	-1.34	-1.22	1.01	1.03	0.96	0.88	0.95	0.85
VMRC-LCD	Lung	1.21	-1.53	-0.20	-3.98	3.81	-1.43	-0.73	-0.99	2.66	0.64	-1.44	0.99	1	0.98	0.89	0.97	0.94
AMO-1	Lymphoid	1.65	4.24	2.25	2.58	2.16	4.16	4.16	-5.18	-1.44	-2.26	-0.69	0.94	0.96	0.99	0.85	0.9	0.72
BJAB	Lymphoid	-4.16	2.20	-5.32	-0.68	3.07	3.59	2.99	-3.69	-2.03	-2.37	-1.30	0.68	0.83	1.01	0.88	0.98	0.95
CA46	Lymphoid	-4.59	-3.19	-9.55	-10.79	-1.22	-4.43	-3.32	-4.69	-4.97	-2.06	-2.04	0.85	0.96	1.06	0.89	1.06	1
CI-1	Lymphoid	1.93	4.28	-6.99	-2.29	0.23	-2.89	3.65	-5.26	1.48	-0.92	-1.46	0.85	0.72	1.08	0.91	1.04	0.97
Daudi	Lymphoid	-5.28	-4.54	-9.55	-7.01	-0.82	-3.03	1.74	-2.53	-2.82	-3.45	-2.53	0.63	0.41	1.03	1.02	1.11	0.99
DB	Lymphoid	-5.84	-3.89	-9.55	-5.37	-1.93	-3.08	-5.74	-4.43	-4.72	-4.64	-2.36	0.93	1.02	1.09	0.96	1.07	1.08
DOHH-2	Lymphoid	-7.54	-3.34	-9.55	-6.34	-3.00	-4.56	-4.01	-5.92	-10.25	-2.50	-3.32	0.52	0.94	0.94	0.81	0.98	0.99
EB-3	Lymphoid	-9.54	-2.11	-5.34	-10.79	-1.43	-5.23	-0.59	-4.68	2.62	-3.02	-1.46	0.94	0.87	1.06	0.95	1.05	1.02
EB1	Lymphoid	-2.98	-4.15	-3.83	-2.98	-2.53	-1.48	1.70	-9.25	-1.78	-0.98	-1.35	0.72	0.72	0.95	0.94	1.01	1.03
EB2	Lymphoid	-2.11	-3.04	-2.54	-3.17	-1.85	-1.14	2.63	-4.53	-0.77	-0.43	-1.34	0.78	0.72	0.99	0.99	1.08	1.06
EJM	Lymphoid	-0.23	-2.14	-6.67	-5.28	-3.40	-1.65	-2.55	-5.37	0.14	-3.77	-2.47	0.87	0.9	0.99	0.93	0.96	0.9
GA-10	Lymphoid	-2.26	-0.93	-5.26	-10.79	-1.17	1.98	-5.06	-9.25	-1.63	-3.14	-1.70	NaN	0.37	0.98	0.93	0.98	1.02
GRANTA-519	Lymphoid	-0.55	-2.64	3.88	-0.62	9.39	3.31	2.45	0.42	-2.54	1.85	-0.86	0.96	1.06	1.05	0.97	1.05	1.02
HEL	Lymphoid	0.17	5.62	3.29	-6.45	3.26	4.78	5.73	-4.55	-6.74	2.03	-1.34	0.99	0.91	0.98	0.84	0.91	0.87
HNT-34	Lymphoid	6.45	6.73	4.56	3.01	-1.01	4.98	4.59	-3.76	-10.25	2.15	-0.96	0.86	0.92	0.94	0.62	0.7	0.46
HS-Sultan	Lymphoid	-7.23	1.66	-9.55	-3.04	-2.81	-3.68	1.84	-7.25	-0.44	0.03	-1.80	0.69	0.96	1	0.95	0.98	0.95
HT	Lymphoid	-9.54	-1.93	-9.55	-1.07	-2.55	4.31	-0.43	-4.95	-0.80	-5.19	-1.78	0.66	0.76	1.06	0.91	0.97	0.95

JEKO-1	Lymphoid	-7.31	-2.84	0.78	-7.00	9.47	-3.40	-3.12	-1.39	-2.95	-2.28	-1.43	0.84	0.46	0.95	0.92	1.13	0.98
Jiyoye	Lymphoid	-3.61	-2.85	-1.07	-4.40	-0.17	-1.52	0.96	-2.42	-0.15	0.14	-1.24	0.8	0.84	1.14	0.88	NaN	0.8
JJN-3	Lymphoid	1.55	2.94	2.12	-1.27	-3.04	1.39	-3.25	-0.85	0.95	0.50	-1.46	0.89	0.93	0.97	0.87	0.96	0.83
K-562	Lymphoid	-3.03	-5.01	1.75	1.38	-0.32	4.64	2.18	-4.28	-6.32	-0.90	-1.83	0.92	0.91	0.94	0.7	0.71	0.5
KARPAS-1106	Lymphoid	3.47	-2.27	-4.31	-3.41	1.53	-0.56	1.45	-9.25	-0.19	-3.16	-1.32	0.86	0.6	1.09	0.77	0.94	0.83
KARPAS-422	Lymphoid	3.79	4.96	-9.55	-2.05	-3.07	4.05	4.96	-5.38	-10.25	2.86	-1.35	0.58	0.79	1.05	0.83	0.97	0.86
KARPAS-620	Lymphoid	3.93	4.72	1.81	2.85	8.46	1.92	3.27	0.55	-2.69	1.23	-1.04	0.63	0.88	1.04	0.71	0.78	0.59
KG-1	Lymphoid	1.15	5.23	2.58	-2.20	-3.35	2.00	2.22	-2.95	-4.15	-0.50	-1.59	1.01	0.95	1.08	0.78	0.85	0.64
KHM-1B	Lymphoid	-5.80	2.32	-9.55	-10.79	-3.89	-0.93	1.83	-6.40	-10.25	-3.94	-4.81	0.69	0.82	1.06	0.85	0.92	0.76
KMM-1	Lymphoid	4.40	1.54	0.65	0.69	-2.06	3.32	1.88	-2.23	-10.25	2.11	-1.80	0.82	0.78	1.04	0.51	0.74	0.27
KMS-11	Lymphoid	1.95	4.30	-0.38	-0.27	-2.74	3.76	3.11	-1.92	-4.85	-2.66	-1.88	0.91	0.79	0.97	0.92	0.99	0.81
KMS-12-BM	Lymphoid	-4.57	-5.20	-6.82	-6.64	7.82	1.18	-5.18	-4.02	-10.25	-1.51	-3.49	0.83	0.84	0.97	0.96	1.02	1.01
KMS-12-PE	Lymphoid	-5.58	-5.69	-9.55	-6.28	7.90	2.95	-5.09	-2.13	-6.57	2.03	-3.74	0.89	0.89	1	1.02	1.04	1.03
KMS-21BM	Lymphoid	2.95	3.76	-0.20	-0.13	7.93	3.87	2.95	-4.80	-10.25	2.96	-0.89	0.87	0.85	0.96	0.62	0.58	0.28
KMS-26	Lymphoid	2.99	1.32	-0.13	-3.63	-3.03	-1.60	-0.60	-5.96	-0.98	2.92	-1.14	0.68	0.71	1	0.89	0.97	0.81
KMS-27	Lymphoid	3.68	0.60	-4.46	0.49	8.01	4.98	3.65	-5.07	0.54	-3.26	-1.39	0.91	0.93	1.03	0.75	0.88	0.55
KMS-28PE	Lymphoid	-1.14	5.47	2.35	-1.02	-2.53	3.33	4.73	-5.13	-6.37	2.99	-1.36	0.74	0.83	1.04	0.84	0.92	0.79
KMS-34	Lymphoid	0.19	0.48	-1.15	-6.14	0.20	-0.30	-0.08	-6.39	-3.94	0.09	-2.12	0.71	0.68	0.96	0.96	1.05	0.94
KU812	Lymphoid	-4.31	2.04	3.09	-0.53	2.75	2.43	2.51	-2.11	-1.25	-0.49	-1.28	0.96	0.96	0.97	0.57	0.7	0.35
L-363	Lymphoid	4.07	2.93	-6.65	-1.19	-1.46	4.48	4.57	-4.89	-6.73	2.06	-2.63	0.68	0.76	0.99	0.91	1.01	0.93
LP-1	Lymphoid	0.48	-3.08	-9.55	-10.79	-0.43	-1.64	-2.41	-3.83	0.00	-2.54	-2.08	0.86	0.82	0.92	0.91	0.99	0.94
MC116	Lymphoid	-4.94	-4.46	-5.13	-3.11	-3.03	-0.09	2.34	-5.95	-5.73	-1.84	-2.04	0.72	NaN	0.94	0.89	1.07	1.06
MHH-PREB-1	Lymphoid	-7.20	2.15	-9.55	0.24	-2.30	-4.67	-4.89	-6.41	-10.25	-2.20	-2.84	0.85	0.92	1.02	0.88	1.02	1.08
ML-2	Lymphoid	1.27	4.68	3.68	-4.62	0.72	3.83	4.44	-3.00	-10.25	1.60	-1.49	0.9	0.79	0.94	0.55	0.66	0.26
MM.1S	Lymphoid	4.50	3.98	-3.26	-3.80	0.27	-1.04	0.97	-9.25	-5.47	3.32	-2.13	0.61	0.81	1.1	0.83	0.89	0.72
MOLM-16	Lymphoid	-9.54	1.56	2.94	-10.79	-0.67	1.49	2.87	-1.66	-2.60	-2.88	-2.56	0.9	0.72	0.99	0.66	0.77	0.5
MOLP-8	Lymphoid	4.05	4.88	1.64	1.65	8.56	2.98	3.99	-4.45	-0.26	1.50	-0.59	0.75	0.76	1.03	0.79	0.9	0.68
MV-4-11	Lymphoid	0.50	5.21	2.17	-2.78	1.88	2.33	3.84	-4.15	-10.25	1.52	-1.88	0.93	0.91	0.99	0.6	0.72	0.46
NALM-6	Lymphoid	-2.06	4.03	3.49	-2.31	-0.40	-1.69	2.58	-3.95	-2.72	0.48	-1.95	0.77	0.7	1.01	0.74	0.84	0.69
NAMALWA	Lymphoid	-9.54	-4.52	-9.55	-6.99	-3.74	-3.18	-3.06	-9.25	2.09	-2.48	-2.04	0.86	NaN	0.98	0.98	1.11	1.04
NCI-H929	Lymphoid	3.17	6.33	-0.98	1.59	4.28	3.98	4.02	-4.80	-0.79	3.25	-0.78	0.64	0.63	1.04	0.77	0.7	0.57
OCI-AML2	Lymphoid	0.12	5.23	4.22	0.28	1.72	3.39	4.58	-4.33	-10.25	-1.64	-1.67	0.92	0.85	1.25	0.15	0.51	0.03
OCI-AML3	Lymphoid	0.79	1.49	5.41	3.13	3.95	2.47	2.97	-2.33	-10.25	1.33	-1.32	0.78	0.89	1.13	0.41	0.58	0.19
OCI-AML5	Lymphoid	-9.54	6.05	3.96	-3.28	0.67	1.49	4.89	-3.92	-6.14	-0.46	-1.47	0.91	0.79	1.04	0.66	0.72	0.54
OCI-LY-10	Lymphoid	-9.54	-2.85	-9.55	-6.25	-3.76	2.45	1.59	-5.84	2.49	5.00	-1.97	0.72	0.42	0.94	0.8	0.93	0.9
OCI-LY-19	Lymphoid	-4.33	5.80	-9.55	2.77	-2.84	4.29	4.10	-9.25	-10.25	-2.67	-1.74	0.85	0.85	1.04	0.65	0.76	0.5
OCI-M1	Lymphoid	-2.65	3.74	3.82	-2.50	5.38	4.83	3.06	-4.77	-3.63	1.44	-1.92	0.99	1	0.98	0.68	0.7	0.56
OCI-M2	Lymphoid	-1.62	4.58	4.59	3.22	3.77	3.86	4.26	-0.06	1.90	1.85	-0.71	0.84	0.8	1.14	0.26	0.5	0.03
OPM-2	Lymphoid	5.39	2.92	0.82	-3.64	-1.48	0.28	1.65	-4.49	-1.45	0.33	-1.29	0.7	0.95	1.02	0.73	0.75	0.73
PL-21	Lymphoid	1.18	4.23	4.40	-4.61	-2.68	0.82	4.05	-5.41	-7.54	0.60	-2.99	0.76	0.74	0.96	0.56	0.71	0.33
RAJI	Lymphoid	-9.54	-4.28	-9.55	-10.79	-2.40	-3.39	2.13	-4.84	-7.87	-1.48	-2.28	0.93	0.95	1.04	0.95	1.04	1.06
REC-1	Lymphoid	-4.36	-2.32	-0.12	-3.53	9.45	1.08	0.34	-3.19	-2.72	2.74	-1.42	0.98	0.82	0.93	0.92	1.01	1.04
RI-1	Lymphoid	-3.63	-2.33	1.91	-6.99	-1.93	0.68	1.03	-3.16	2.44	-0.61	-1.79	0.75	0.57	1.01	0.92	1.03	0.97
RL	Lymphoid	-5.24	-0.66	-4.21	-4.25	-2.63	0.81	-2.84	-4.68	-4.08	-2.67	-1.97	0.61	0.89	1	0.91	1	1.03
RPMI 8226	Lymphoid	3.89	2.97	-9.55	-1.17	2.87	4.18	3.84	-0.47	-8.12	-1.80	-1.48	0.94	0.93	0.97	0.79	0.85	0.59
SC-1	Lymphoid	-4.80	-1.58	-5.55	-4.86	-2.11	-2.98	-3.53	-2.83	-10.25	0.39	-3.64	0.95	1.04	0.99	0.95	0.97	1.01

SCC-3	Lymphoid	4.47	3.24	4.61	1.68	3.41	3.28	3.72	-5.53	-0.02	2.66	-0.74	0.99	1.02	1.04	0.91	0.98	0.91
SET-2	Lymphoid	-3.78	1.02	2.37	-4.61	2.07	3.67	2.53	-2.00	-0.27	-1.71	-2.34	0.99	0.92	1.05	0.83	0.84	0.78
SKM-1	Lymphoid	-0.01	2.14	4.65	3.29	0.75	4.86	4.44	-1.43	-2.15	-1.62	-1.34	0.95	0.81	1	0.47	0.66	0.18
SR	Lymphoid	3.25	3.51	-4.31	2.22	-2.88	2.40	3.11	-4.92	-5.87	0.51	-1.37	0.89	0.91	0.93	0.85	0.9	0.84
SU-DHL-1	Lymphoid	-1.73	1.51	-4.20	2.65	-3.17	5.35	4.69	-3.54	-6.32	-0.01	-1.60	0.87	0.93	1.02	0.78	0.79	0.69
SU-DHL-16	Lymphoid	-4.18	4.34	-4.38	-2.49	-1.47	1.98	3.01	-2.51	-3.41	-1.96	-0.92	0.56	0.68	1.01	0.74	1.06	0.89
SU-DHL-4	Lymphoid	-9.54	-0.66	-4.26	-10.79	-1.18	-2.87	-0.39	-2.73	-1.56	-3.36	-1.91	0.55	0.42	1.09	0.93	1.07	0.97
SU-DHL-5	Lymphoid	-9.54	1.42	-4.33	-0.20	-1.73	1.26	3.57	-4.24	-10.25	-0.86	-1.39	0.51	NaN	0.99	0.83	0.88	0.86
SU-DHL-6	Lymphoid	-4.80	-1.86	-4.99	-3.48	-1.32	0.79	1.84	-3.36	-10.25	-1.23	-1.92	0.56	0.41	1	0.82	0.94	0.86
SU-DHL-8	Lymphoid	-2.36	5.60	-3.60	-0.38	-2.54	3.95	5.70	-5.22	-7.05	-0.02	-3.35	0.96	0.96	0.97	0.88	1.04	0.79
SUP-B15	Lymphoid	0.01	6.81	5.72	1.02	0.20	0.53	5.35	1.04	-2.02	-1.46	-1.30	0.93	0.98	0.98	0.7	0.72	0.58
TMD8	Lymphoid	-1.94	-2.92	-9.55	-3.78	-0.65	0.30	1.03	-4.22	5.04	4.56	-1.24	0.88	0.5	1.05	0.79	0.86	0.75
U-698-M	Lymphoid	-5.40	-2.96	-9.55	-6.28	-1.82	-3.48	-5.36	-3.86	-10.25	-3.04	-0.57	0.83	0.99	0.96	0.89	1.03	1.07
U-937	Lymphoid	2.35	3.96	3.73	0.30	-0.39	3.64	4.14	-1.99	-10.25	-0.95	-1.53	0.73	1.02	1.02	0.81	0.91	0.88
UKE-1	Lymphoid	1.56	6.13	3.84	-1.40	3.93	2.36	2.49	1.23	-6.68	-3.04	-0.84	0.83	0.6	0.99	0.5	0.62	0.28
WSU-DLCL2	Lymphoid	-9.54	2.04	-5.24	-5.51	-1.51	-0.35	-3.60	-3.61	-5.27	-2.35	-1.46	0.3	0.99	0.98	0.84	1.09	1.04
WSU-FSCCL	Lymphoid	-1.00	4.86	1.24	-1.15	-2.35	3.72	4.79	-3.47	-10.25	-1.61	-1.05	0.86	0.74	0.98	0.67	1.18	0.87
DU 145	Other	0.55	1.86	2.74	0.66	6.93	3.44	4.04	6.04	-1.38	5.70	-0.75	0.91	0.73	0.89	0.69	0.81	0.53
NCI-H2052	Other	0.32	3.93	3.36	3.27	8.38	0.79	3.31	6.12	2.64	5.67	-0.40	0.93	0.93	0.94	0.86	1.04	0.93
NCI-H28	Other	2.02	1.46	3.09	0.99	7.23	0.40	2.14	5.50	1.37	4.89	-0.55	0.9	0.89	0.98	0.92	0.95	0.91
NTERA-2	Other	1.29	5.67	5.05	4.67	7.20	6.63	5.26	5.25	1.87	5.08	-0.07	0.78	0.69	0.89	0.93	0.81	0.75
PC-3	Other	6.04	4.83	3.87	5.15	8.28	5.95	4.26	7.31	-0.23	6.32	-0.29	0.82	0.75	0.94	0.93	0.95	0.92
SU-DHL-10	Other	-1.82	0.84	-6.38	-4.86	-2.53	-1.10	-2.82	-4.41	-0.14	-2.96	-1.74	0.62	0.89	1.21	0.93	0.9	0.75
59M	Ovary	2.08	2.00	2.09	1.72	6.20	2.93	4.05	5.10	-0.78	4.24	-0.40	0.89	0.84	0.95	0.87	1.01	0.94
A2780	Ovary	-5.62	5.70	6.83	4.30	5.91	4.47	4.62	3.55	-4.89	-1.14	-0.65	0.81	0.86	0.92	0.71	0.8	0.56
COV318	Ovary	1.38	2.55	3.06	2.72	7.85	2.61	2.95	7.83	-1.16	1.32	-0.69	0.96	0.89	1	0.95	0.97	0.94
COV362	Ovary	4.33	3.73	3.71	3.78	8.81	4.65	4.75	7.52	2.19	5.04	-0.29	0.97	0.9	0.95	0.8	0.89	0.77
COV413A	Ovary	3.47	5.56	5.32	4.65	8.08	6.19	4.23	5.97	0.51	3.52	-0.25	0.81	0.75	0.94	0.65	0.76	0.52
COV413B	Ovary	2.35	3.75	4.04	2.65	6.73	4.26	3.63	5.72	-3.33	5.20	-0.61	0.93	0.81	1	0.84	0.84	0.72
COV434	Ovary	-4.04	4.56	1.57	2.62	3.13	5.55	2.89	2.12	-3.35	-0.66	-0.87	0.91	0.9	0.97	0.77	0.91	0.8
COV504	Ovary	4.24	3.94	4.33	3.72	6.97	5.19	5.52	7.03	-0.97	5.79	-0.36	0.93	0.88	1.02	0.86	0.89	0.79
EFO-27	Ovary	4.33	1.36	3.48	0.53	7.38	3.05	3.42	6.64	2.67	5.55	-0.30	0.57	0.92	0.99	0.74	0.81	0.67
ES-2	Ovary	2.78	5.77	6.86	3.67	8.19	4.01	5.05	5.84	2.92	5.74	-0.25	0.96	1	0.86	0.53	0.74	0.45
HEY	Ovary	0.74	4.48	5.12	4.42	7.80	4.73	4.79	5.73	0.84	5.57	-0.35	0.89	0.9	0.9	0.63	0.68	0.47
Hs 38.T	Ovary	2.76	4.25	4.57	3.45	8.49	2.81	4.09	5.14	1.58	6.57	-0.18	0.86	0.85	0.86	0.77	0.82	0.75
IGROV-1	Ovary	2.76	6.65	6.60	4.30	7.17	4.83	5.06	4.03	1.90	3.59	-0.32	0.65	0.57	0.95	0.54	0.62	0.37
KURAMOCHI	Ovary	-1.49	3.27	4.76	3.88	5.87	4.13	5.23	6.70	-0.20	1.07	-0.62	0.91	0.81	0.99	0.75	0.88	0.74
MCAS	Ovary	4.73	6.18	4.81	3.40	7.58	3.78	4.18	6.57	1.60	6.25	-0.26	0.85	0.89	0.87	0.47	0.59	0.31
NIH:OVCAR-3	Ovary	1.69	3.04	3.48	2.91	6.68	4.36	3.24	6.66	1.51	3.29	-0.34	0.82	0.68	0.99	0.81	0.87	0.76
OAW42	Ovary	-2.50	5.75	4.31	3.77	6.23	4.21	4.74	4.70	-3.47	4.54	-0.84	0.78	0.69	0.93	0.76	0.74	0.62
ONCO-DG-1	Ovary	1.26	2.52	2.86	1.71	7.16	3.86	2.47	6.78	1.50	-1.20	-0.57	0.75	0.64	0.91	0.79	0.81	0.74
OV-90	Ovary	4.60	6.03	6.33	4.51	4.87	5.63	4.55	4.87	3.73	4.74	-0.27	0.85	0.94	0.93	0.5	0.7	0.38
OV56	Ovary	2.81	3.84	3.62	2.04	6.41	3.63	4.16	5.00	2.70	6.63	-0.34	0.86	0.89	0.91	0.76	0.78	0.67
OV7	Ovary	4.38	4.79	3.53	4.04	7.35	3.45	4.35	6.27	2.19	6.01	-0.37	0.95	0.86	0.99	0.84	0.87	0.74
OVCA 420	Ovary	4.51	5.28	-0.31	0.96	6.32	3.50	2.95	5.64	-6.80	4.05	-0.73	0.81	0.86	0.93	0.68	0.8	0.63
OvCA 429	Ovary	3.94	2.12	3.27	0.79	7.53	2.59	2.72	6.09	-0.05	4.27	-0.54	0.89	0.84	0.89	0.54	0.76	0.51
OvCA 432	Ovary	-1.49	4.97	3.88	4.69	8.02	6.32	4.07	5.50	-0.55	1.29	-0.50	0.86	0.84	0.93	0.97	0.96	0.96

OVCAR-8	Ovary	-0.50	1.74	3.90	2.99	7.87	3.91	4.55	5.49	0.09	3.65	-0.68	0.93	0.89	0.97	0.77	0.84	0.68
OVCAR433	Ovary	4.05	3.35	3.70	2.60	8.88	3.53	3.73	7.27	-0.31	5.19	-0.47	0.87	0.84	0.95	0.63	0.75	0.5
OVISE	Ovary	2.61	0.58	4.00	3.08	7.39	4.74	3.48	5.92	3.59	2.78	-0.30	0.81	0.81	0.93	0.76	0.82	0.69
OVSAHO	Ovary	-6.23	4.36	4.63	2.09	4.87	4.86	4.73	5.04	-2.17	1.20	-0.96	0.93	0.86	0.98	0.81	0.91	0.79
OVTOKO	Ovary	1.64	4.52	3.27	1.71	6.42	3.42	3.27	5.76	0.92	5.67	-0.46	0.85	0.71	0.94	0.87	0.9	0.87
PA-1	Ovary	-2.61	5.46	5.20	4.79	7.86	5.31	3.84	3.99	1.67	5.15	-0.35	0.8	0.79	0.91	0.75	0.84	0.67
PE01	Ovary	5.18	3.59	1.95	1.78	8.11	6.00	3.92	5.35	-0.31	4.26	-0.48	0.91	0.94	0.95	0.76	0.78	0.63
RKN	Ovary	2.97	4.64	4.46	4.54	6.24	5.05	4.62	6.11	-0.53	5.69	-0.27	0.88	0.82	0.87	0.59	0.71	0.43
RMG-I	Ovary	2.67	3.47	5.06	2.03	5.88	4.81	3.42	5.89	-3.00	1.79	-0.59	0.87	0.83	0.92	0.68	0.83	0.58
RMUG-S	Ovary	4.77	3.07	2.30	0.17	5.91	1.35	2.71	4.65	1.21	4.69	-0.55	0.87	0.82	0.98	0.67	0.75	0.59
SK-OV-3	Ovary	2.14	3.68	3.11	2.54	6.18	3.02	3.37	5.91	1.07	6.25	-0.42	0.82	0.78	0.92	0.9	0.84	0.82
TOV-112D	Ovary	-3.34	7.15	1.82	1.62	6.11	5.75	4.09	3.15	-1.90	1.50	-0.80	0.91	0.94	0.96	0.79	0.88	0.78
TYK-nu	Ovary	-1.01	7.76	6.86	6.03	6.29	4.97	4.56	5.95	-3.49	6.82	-0.40	0.88	1	0.98	0.66	0.71	0.48
TYK-nu.CP-r	Ovary	-0.63	7.11	5.76	5.68	7.02	4.65	4.73	5.64	-4.14	5.60	-0.50	0.84	0.94	0.97	0.55	0.67	0.47
AsPC-1	Pancreas	5.35	6.75	5.65	4.88	6.29	5.07	4.37	6.30	-1.75	6.58	-0.35	0.78	0.84	1.02	0.61	0.69	0.5
BxPC-3	Pancreas	5.37	5.95	3.97	1.89	6.25	3.66	2.20	6.85	0.54	6.30	-0.41	0.87	0.86	0.96	0.74	0.79	0.71
Capan-1	Pancreas	4.33	5.21	3.16	3.25	7.09	5.95	4.81	6.79	2.03	6.36	-0.20	1.02	0.94	1.06	0.79	0.91	0.79
Capan-2	Pancreas	4.03	3.24	3.71	2.99	8.50	3.66	3.80	7.02	-1.19	5.81	-0.64	0.97	0.83	0.97	0.61	0.65	0.42
CFPAC-1	Pancreas	4.47	5.04	3.93	3.94	6.99	4.56	4.01	6.96	1.50	5.15	-0.39	0.91	0.83	0.94	0.71	0.75	0.6
DAN-G	Pancreas	5.05	6.20	4.53	3.88	7.51	5.49	2.68	6.41	1.69	6.01	-0.26	0.84	0.81	0.95	0.82	0.82	0.68
HPAC	Pancreas	4.55	6.62	4.45	3.48	8.59	4.13	3.28	7.66	3.92	6.87	-0.17	0.92	0.85	0.92	0.54	0.62	0.4
HPAF-II	Pancreas	4.17	8.00	2.88	5.52	7.70	4.47	2.54	7.09	0.73	7.51	-0.30	0.88	0.86	0.98	0.55	0.63	0.42
Hs 766T	Pancreas	7.15	6.37	5.89	4.35	6.37	4.35	4.96	7.99	1.24	4.95	-0.22	0.8	0.83	0.96	0.51	0.64	0.33
HUP-T3	Pancreas	3.32	2.31	4.14	3.62	7.60	4.59	3.58	7.11	-1.86	5.27	-0.44	0.93	0.9	0.89	0.67	0.76	0.52
HUP-T4	Pancreas	3.68	3.97	3.20	1.36	8.11	3.74	0.97	7.63	1.38	4.75	-0.46	0.88	0.79	0.95	0.6	0.72	0.45
KCI-MOH1	Pancreas	4.84	5.55	4.10	2.70	8.76	3.72	3.25	7.16	3.13	6.25	-0.29	NaN	0.79	0.94	0.56	0.73	0.49
KLM-1	Pancreas	3.62	4.28	2.36	2.95	6.29	4.92	4.08	5.99	0.56	5.07	-0.52	0.86	0.89	0.96	0.78	0.84	0.61
KP-3	Pancreas	4.36	3.53	5.09	2.28	6.46	2.35	4.44	7.06	2.12	6.44	-0.29	0.97	0.86	0.92	0.81	0.85	0.72
KP-3L	Pancreas	6.32	5.57	4.42	4.04	7.29	6.00	4.96	7.45	1.48	6.45	-0.03	0.87	0.83	0.92	0.62	0.7	0.53
KP4	Pancreas	0.03	5.36	4.58	5.02	6.64	5.17	4.94	5.59	-1.20	6.64	-0.40	0.81	0.96	0.95	0.7	0.73	0.62
MIA Paca-2	Pancreas	-4.09	1.60	6.45	5.98	5.60	4.59	5.16	5.92	4.96	6.11	-0.61	0.89	0.89	0.97	0.58	0.72	0.41
PA-TU-8902	Pancreas	6.05	4.16	4.35	4.18	7.04	6.06	4.66	6.73	-7.41	6.02	-0.31	0.81	0.86	1	0.64	0.7	0.44
PA-TU-8988S	Pancreas	2.15	5.22	4.64	3.34	6.13	5.46	4.68	5.29	2.58	3.98	-0.49	0.96	0.87	0.99	0.67	0.7	0.55
PA-TU-8988T	Pancreas	3.76	4.77	3.73	2.55	6.90	4.20	5.30	6.61	-2.48	4.34	-0.42	0.91	0.82	0.93	0.71	0.81	0.63
Panc 02.03	Pancreas	3.93	4.61	2.24	2.00	7.36	4.25	4.37	7.16	-1.35	6.77	-0.33	0.87	0.74	0.98	0.45	0.57	0.22
Panc 03.27	Pancreas	4.70	5.19	4.97	3.99	7.65	4.83	4.62	6.59	1.10	5.41	-0.26	0.84	0.83	0.91	0.66	0.68	0.51
Panc 04.03	Pancreas	4.78	4.97	2.40	1.88	8.02	4.72	3.87	7.05	0.16	6.68	-0.48	0.88	0.78	0.95	0.6	0.72	0.47
Panc 05.04	Pancreas	5.87	5.93	4.88	3.42	7.99	4.13	1.50	6.44	2.98	5.42	-0.25	0.87	0.74	0.97	0.56	0.71	0.4
PANC-1	Pancreas	-5.28	2.69	3.49	3.53	6.43	2.57	4.53	7.10	-1.87	6.28	-0.71	0.96	0.92	1	0.79	0.85	0.69
PK-45H	Pancreas	4.54	3.16	4.92	2.37	7.35	4.06	4.39	5.64	2.05	4.80	-0.54	0.85	0.78	1	0.51	0.64	0.36
PK-45P	Pancreas	5.31	4.64	5.20	2.31	6.74	4.83	4.89	5.94	-4.47	5.99	-0.56	0.84	0.83	1	0.63	0.75	0.47
PK-59	Pancreas	6.39	5.17	3.13	4.55	6.73	5.63	2.88	7.01	2.40	6.17	-0.27	1	0.82	0.93	0.68	0.74	0.56
PK-8	Pancreas	2.91	4.89	2.81	2.82	7.03	3.80	2.39	5.99	1.69	6.49	-0.60	0.9	0.84	0.94	0.59	0.72	0.4
PSN1	Pancreas	4.97	7.14	4.39	5.09	6.79	4.99	5.43	4.14	2.92	6.78	-0.29	0.88	0.85	0.96	0.42	0.61	0.22
QGP-1	Pancreas	5.47	6.34	4.53	5.68	5.58	5.87	4.17	0.74	3.08	4.76	-0.53	0.99	0.94	0.93	0.76	0.83	0.69
SU.86.86	Pancreas	5.79	6.73	4.07	3.23	7.09	5.51	5.19	7.18	0.29	6.51	-0.02	0.97	0.95	0.88	0.72	0.79	0.68

SUIT-2	Pancreas	2.77	4.38	3.18	3.56	6.12	4.23	4.39	6.69	1.46	4.83	-0.44	0.96	0.87	1.02	0.58	0.65	0.35
SW 1990	Pancreas	6.27	4.86	5.91	4.10	7.05	4.90	4.56	6.37	-0.14	5.62	-0.40	0.89	0.93	0.92	0.71	0.75	0.55
TCC-PAN2	Pancreas	4.72	5.30	5.46	5.08	7.72	3.72	4.18	7.48	1.68	6.81	-0.25	0.9	0.81	1.08	0.67	0.8	0.53
YAPC	Pancreas	5.62	6.38	5.43	4.02	8.09	6.30	4.12	6.58	2.66	6.71	-0.18	0.92	0.84	0.92	0.78	0.79	0.69
105KC	Sarcomatoid	1.42	0.39	4.11	2.33	5.08	3.66	4.19	5.34	0.18	6.57	-0.63	0.94	0.89	0.92	0.87	0.95	0.86
143B	Sarcomatoid	2.12	5.45	2.85	4.25	7.90	5.16	4.73	5.96	0.79	4.37	-0.48	0.93	0.95	0.95	0.66	0.69	0.49
MG-63	Sarcomatoid	-5.16	3.92	2.92	3.73	6.26	1.79	5.15	5.26	1.09	3.76	-0.52	NaN	NaN	0.93	0.84	0.9	0.84
MHH-ES-1	Sarcomatoid	-4.03	5.68	0.79	0.12	9.01	-2.44	-0.44	4.21	2.51	-0.66	-0.89	0.92	0.82	0.94	0.94	0.99	0.95
SJCRH30	Sarcomatoid	-0.82	4.62	5.71	3.71	5.17	4.98	4.44	5.48	0.22	2.46	-0.42	0.74	0.76	0.97	0.91	0.93	0.86
TC-71	Sarcomatoid	-4.31	3.11	2.03	-1.22	8.22	-3.22	4.07	2.81	2.20	-0.54	-0.86	0.87	0.63	0.94	0.83	0.92	0.77
U-2 OS	Sarcomatoid	-5.20	-2.27	1.72	-0.82	6.49	5.56	3.97	6.08	-0.75	1.98	-0.78	0.87	0.75	1.02	0.7	0.75	0.57
501A	Skin	4.07	5.69	3.57	3.95	7.08	6.17	6.10	2.71	2.69	4.65	-0.32	0.9	0.83	0.76	0.46	0.71	0.33
537 MEL	Skin	6.27	5.07	5.84	5.47	8.41	4.14	5.53	2.49	-7.17	6.42	-0.44	0.68	0.83	0.92	0.43	0.7	0.31
624 mel	Skin	4.94	5.39	4.49	3.68	8.85	6.01	7.14	1.53	2.09	5.02	-0.29	0.86	0.89	0.74	0.45	0.66	0.39
928 mel	Skin	5.95	6.07	4.77	3.90	8.13	5.77	6.44	1.80	5.01	5.93	-0.17	0.92	0.86	0.76	0.4	0.66	0.3
A-375	Skin	5.32	6.12	6.52	4.80	7.40	6.08	4.81	6.69	1.74	6.33	-0.10	0.91	0.93	0.63	0.29	0.59	0.15
A-431	Skin	2.01	4.05	-1.03	1.25	8.72	3.58	0.56	6.37	-0.08	5.52	-0.62	0.85	0.93	0.97	0.72	0.79	0.57
A2058	Skin	5.50	5.65	5.82	3.32	6.54	5.87	5.09	4.72	3.20	5.64	-0.25	0.86	0.9	0.86	0.52	0.67	0.4
C32	Skin	4.64	6.42	6.70	6.65	8.20	6.28	5.77	2.49	-7.45	6.58	-0.23	0.88	0.88	0.7	0.34	0.59	0.13
C32TG	Skin	5.07	5.81	5.77	5.48	8.12	5.37	5.22	5.84	-0.53	6.68	-0.09	0.8	0.87	0.71	0.39	0.69	0.29
CHL-1	Skin	0.91	4.79	5.33	1.90	7.17	4.56	5.56	5.04	-7.87	1.64	-0.74	0.91	0.98	0.91	0.74	0.79	0.71
COLO 679	Skin	7.37	7.83	5.99	6.43	7.51	5.84	6.47	2.24	2.71	7.66	-0.12	0.96	0.86	0.71	0.37	0.7	0.33
COLO 794	Skin	5.52	6.01	6.00	4.78	7.94	5.82	6.08	5.91	-1.18	6.40	-0.19	0.81	0.85	0.69	0.35	0.68	0.23
COLO 800	Skin	6.39	5.69	6.33	6.00	7.79	5.23	6.99	4.02	-3.44	6.65	-0.15	0.86	0.89	0.73	0.32	0.67	0.19
COLO 829	Skin	6.58	6.84	6.64	6.22	6.86	6.67	6.32	2.30	-1.30	7.03	-0.18	0.87	0.87	0.71	0.39	0.68	0.28
COLO 849	Skin	6.74	6.37	5.65	6.33	6.54	6.09	7.02	1.97	-1.56	6.65	-0.25	0.9	0.93	0.74	0.46	0.72	0.33
COLO 853	Skin	6.69	6.56	6.18	5.55	8.82	6.41	6.26	4.51	-0.70	8.10	-0.01	0.8	0.95	0.72	0.41	0.66	0.31
COLO 857	Skin	5.63	6.56	6.60	6.92	8.71	6.02	6.47	5.04	-2.76	7.59	-0.05	0.83	0.94	0.68	0.38	0.66	0.29
DEOC-1	Skin	4.66	5.86	6.55	4.62	7.10	5.33	6.21	4.92	-3.78	7.00	-0.08	0.92	0.91	1.08	0.61	0.76	0.37
G-361	Skin	6.54	5.06	4.50	4.23	5.52	6.18	5.10	2.31	3.28	5.71	-0.30	0.83	0.84	0.69	0.29	0.63	0.16
GR-M	Skin	5.04	6.63	3.89	5.68	6.26	5.08	4.78	6.08	2.03	7.14	-0.17	0.86	0.9	0.87	0.45	0.65	0.23
HMY-1	Skin	6.10	4.58	5.81	4.38	7.57	5.46	5.63	3.15	0.24	5.58	-0.25	0.91	0.84	0.67	0.38	0.7	0.3
Hs 294T	Skin	5.38	5.15	6.32	3.83	6.98	4.92	4.84	5.71	1.16	6.69	-0.29	0.85	0.89	0.77	0.46	0.72	0.47
Hs 852.T	Skin	4.33	6.04	6.80	2.86	10.44	3.20	4.00	4.84	1.06	6.14	-0.60	0.87	0.8	0.98	0.59	0.69	0.44
IGR-37	Skin	6.62	3.91	5.31	3.76	6.56	5.65	5.67	1.55	1.49	5.03	-0.32	0.89	0.87	0.74	0.39	0.67	0.27
IPC-298	Skin	6.36	6.21	5.91	5.83	6.52	5.81	6.09	3.00	-5.03	6.37	-0.35	0.93	0.82	0.87	0.38	0.58	0.09
LOX-IMVI	Skin	-9.54	4.79	4.37	3.14	6.69	4.58	4.73	4.88	-0.38	6.52	-0.58	1.17	NaN	0.94	0.63	0.68	0.52
MDA-MB-435	Skin	5.24	4.07	6.36	3.96	7.02	5.70	6.39	4.51	-2.78	5.98	-0.28	0.91	0.85	0.85	0.74	0.91	0.75
MEL-HO	Skin	7.01	5.52	4.61	3.98	8.61	6.25	7.10	3.42	1.94	6.62	-0.16	0.83	0.84	0.75	0.36	0.65	0.26
MeWo	Skin	6.06	4.97	4.33	2.78	6.20	4.49	5.04	1.95	1.29	6.09	-0.38	0.91	0.82	0.9	0.55	0.68	0.42
RPMI-7951	Skin	5.49	4.42	4.73	4.09	6.86	5.38	5.14	6.08	0.91	5.96	-0.22	0.88	0.94	0.86	0.62	0.77	0.52
SK-MEL-1	Skin	6.25	5.65	5.90	7.33	9.24	7.17	7.43	5.09	-10.25	8.04	-0.24	0.96	0.96	0.84	0.41	0.64	0.18
SK-MEL-2	Skin	6.26	6.54	5.48	5.80	8.42	6.76	6.43	4.92	1.65	5.56	-0.06	1.1	1.18	1.02	0.57	0.59	0.55
SK-MEL-24	Skin	5.86	6.38	6.03	6.14	7.51	4.92	6.11	6.51	1.98	7.12	-0.03	0.87	0.85	0.62	0.24	0.64	0.17
SK-MEL-28	Skin	6.38	5.33	5.72	4.79	8.21	5.19	6.83	-0.94	2.35	5.52	-0.18	0.89	0.8	0.68	0.34	0.66	0.26
SK-MEL-3	Skin	6.05	4.55	5.41	4.29	5.16	4.29	5.74	3.59	2.57	6.21	-0.29	0.92	0.82	0.75	0.59	0.73	0.49
SK-MEL-30	Skin	5.65	5.93	5.34	4.70	7.32	6.14	6.68	1.60	0.60	5.70	-0.20	0.89	0.93	0.93	0.37	0.62	0.16

SK23	Skin	6.68	4.35	5.81	4.00	7.17	5.78	5.44	3.17	-10.25	6.18	-0.49	0.87	1.02	0.83	0.31	0.65	0.24
UCSD-242l	Skin	6.15	6.08	5.96	5.16	7.52	6.29	6.00	4.08	-0.58	6.66	-0.12	0.82	0.79	0.69	0.47	0.74	0.38
WM-115	Skin	6.17	6.43	6.01	7.51	8.47	5.30	5.50	6.69	1.18	6.80	0.06	0.87	0.83	0.89	0.74	0.89	0.7
WM-266-4	Skin	6.62	5.98	6.45	6.92	8.63	6.31	5.92	2.26	-5.81	6.31	-0.25	0.8	0.79	0.69	0.22	0.61	0.13
23132/87	Stomach	2.79	4.31	3.07	1.35	6.65	5.09	0.18	5.72	-4.05	6.73	-0.76	0.81	0.83	0.98	0.84	0.84	0.76
AGS	Stomach	5.62	5.76	3.41	3.95	7.90	4.87	5.53	6.90	3.87	5.20	-0.10	0.77	0.82	0.91	0.6	0.74	0.48
FU97	Stomach	-5.48	3.58	-0.96	3.52	7.12	5.44	4.87	5.03	-6.63	2.60	-1.26	0.81	0.82	0.96	0.73	0.84	0.77
GTL-16	Stomach	2.97	4.81	2.99	2.51	6.29	6.01	5.77	5.53	-1.08	6.93	-0.68	0.83	0.8	1.02	0.57	0.64	0.34
HGC-27	Stomach	-2.99	-4.00	0.83	-4.91	6.20	-0.55	0.07	3.78	0.40	-0.55	-1.68	0.54	0.58	0.93	0.84	0.88	0.91
Hs 746T	Stomach	-2.72	5.15	5.75	4.23	7.93	3.14	5.38	6.60	-0.22	7.07	-0.47	0.83	0.8	0.97	0.71	0.72	0.61
KATO III	Stomach	6.27	5.54	3.08	2.55	6.96	4.81	4.80	6.66	0.09	4.95	-0.36	0.81	0.87	1.03	0.81	0.82	0.71
MKN-1	Stomach	2.81	3.61	2.52	2.14	7.21	4.52	3.92	6.21	3.19	4.15	-0.42	0.81	0.78	0.95	0.79	0.83	0.73
MKN-45	Stomach	4.16	5.46	5.18	3.46	6.49	5.38	5.69	5.20	-0.66	6.91	-0.56	0.82	0.84	0.94	0.64	0.69	0.44
MKN-74	Stomach	3.54	4.38	1.97	5.58	7.73	5.76	4.62	8.66	2.42	7.12	-0.02	0.93	0.9	0.94	0.66	0.74	0.56
NCI-N87	Stomach	-0.89	5.44	1.90	3.07	6.87	3.62	2.91	5.23	0.99	5.36	-0.46	0.85	0.76	1.02	0.84	0.82	0.77
NUGC-3	Stomach	5.18	6.00	2.02	3.37	8.75	4.62	3.70	7.23	-0.44	5.71	-0.48	0.86	0.87	0.91	0.65	0.8	0.5
NUGC-4	Stomach	1.95	3.86	2.44	1.54	6.14	4.58	2.61	3.91	-5.03	2.78	-0.77	0.91	0.92	1.06	0.71	0.76	0.63
OCUM-1	Stomach	6.45	6.71	6.98	4.93	7.34	3.45	2.65	6.76	-3.20	7.54	-0.25	0.84	0.89	1.04	0.33	0.48	0.15
RERF-GC-1B	Stomach	0.64	3.18	4.01	1.44	5.99	4.40	3.01	4.32	0.21	1.38	-0.98	0.9	0.87	1.01	0.64	0.74	0.52
SNU-16	Stomach	3.28	4.71	3.64	2.39	6.70	4.21	1.05	4.44	-2.26	1.94	-0.70	0.9	0.93	0.93	0.82	0.82	0.77
SNU-216	Stomach	3.38	-0.02	2.18	-2.12	7.00	1.57	0.80	6.10	1.18	4.69	-1.39	0.71	0.61	0.9	0.61	0.77	0.58
SNU-484	Stomach	-2.91	7.79	5.82	3.45	4.03	5.63	5.30	5.06	-2.68	-0.61	-1.17	0.9	0.86	0.99	0.81	0.85	0.7
SNU-601	Stomach	5.30	4.98	3.74	3.11	7.24	5.22	4.32	6.83	1.83	5.20	-0.68	0.79	0.67	0.97	0.68	0.64	0.43
SNU-620	Stomach	5.93	4.49	3.06	2.92	4.81	5.44	5.62	4.86	-7.62	4.43	-0.77	0.85	0.94	1.08	0.5	0.6	0.14
SNU-638	Stomach	6.41	6.62	4.83	4.12	7.53	4.67	4.94	4.37	1.88	7.94	-0.25	0.84	0.86	1.01	0.73	0.78	0.61
SNU-668	Stomach	5.56	6.39	3.96	3.33	7.07	4.06	3.31	5.36	2.40	4.27	-0.72	0.89	0.84	0.98	0.64	0.77	0.59
SNU-719	Stomach	3.98	7.01	4.28	3.93	6.44	6.26	3.17	6.98	-1.86	6.56	-0.51	0.98	0.94	0.99	0.59	0.56	0.36
A-673	Unknown	1.06	5.76	2.81	2.92	9.72	3.90	4.30	5.17	1.44	1.40	-0.60	0.98	0.82	0.91	0.85	0.9	0.78
BHY	Unknown	4.62	5.49	3.07	2.60	6.64	3.85	1.18	5.88	-1.75	6.33	-0.51	0.86	0.89	0.93	0.74	0.85	0.75
BICR 10	Unknown	3.78	3.05	3.37	2.54	6.82	4.02	4.33	5.94	-0.07	5.62	-0.39	0.66	0.69	0.95	0.8	0.83	0.74
Detroit 562	Unknown	2.57	2.83	2.44	0.05	8.06	3.95	1.93	6.27	-1.61	4.63	-0.63	0.69	0.8	0.9	0.63	0.77	0.55
FaDu	Unknown	0.41	2.27	0.98	-0.30	7.79	2.76	1.37	5.94	-1.47	2.26	-1.31	0.9	0.87	0.98	0.71	0.83	0.61
HN	Unknown	1.79	4.24	3.47	2.26	5.67	3.75	2.05	5.39	-2.51	5.49	-0.75	0.85	0.83	0.97	0.68	0.78	0.58
SW 780	Unknown	4.47	3.03	1.74	1.13	7.37	2.64	2.00	7.06	0.08	5.40	-0.92	0.91	0.77	0.99	0.72	0.78	0.62
U266B1	Unknown	0.23	2.61	-3.05	-4.41	8.35	2.76	3.12	-4.34	-5.19	-2.46	-1.02	0.92	0.89	1	0.8	0.86	0.69
RT-112	Bladder	2.93	5.45	2.39	0.35	6.28	4.77	2.54	6.15	2.94	6.29	-0.43	0.83	0.75	0.97	0.62	0.71	0.44
RT4	Bladder	2.71	5.36	2.59	4.28	7.24	3.36	2.47	4.75	0.83	6.07	-0.59	1.01	0.93	1.06	0.76	0.89	0.83
UM-UC-1	Bladder	2.26	5.17	2.97	1.67	7.54	4.38	2.32	5.79	2.60	6.37	-0.39	0.68	0.71	0.99	0.63	0.66	0.43
UM-UC-3	Bladder	4.47	2.68	3.46	3.88	7.85	3.99	4.64	6.40	-2.25	6.15	-0.44	0.81	0.83	1.03	0.71	0.8	0.57
AN3 CA	Uterus	0.73	7.56	7.12	6.20	-0.13	6.34	5.23	3.58	-7.64	3.98	-0.74	0.74	0.74	0.98	0.77	0.93	0.84
HCC1482	Uterus	4.34	5.15	5.64	5.55	5.78	4.32	5.84	3.68	4.21	6.81	-0.12	0.71	0.75	0.93	0.84	0.92	0.84
HEC-1-A	Uterus	-2.99	6.37	5.72	4.35	6.49	5.52	5.30	6.55	0.05	3.07	-0.38	0.83	0.8	0.95	0.72	0.76	0.65
KLE	Uterus	2.69	4.48	3.42	3.90	5.49	4.67	3.98	7.43	1.27	1.91	-0.39	0.95	0.8	1.03	0.87	0.95	0.85

Supplementary Table 2: MAPK gene expression, MPAS and cell viability in response to multiple MAPK inhibitors across cell lines from multiple indications

Supplementary Table 3

cell line indication		Mean Viability (STD)					
	MPAS (STD)	GDC0068	GDC0941	Cobimetinib	PD0325901	GDC0623	Vemurafenib
		(AKT)	(PI3K)	(MEK)	(MEK)	(MEK)	(BRAF)
**Breast**	0.454	0.110	0.082	0.101	0.153	0.108	0.041
**Lung**	0.478	0.083	0.075	0.138	0.199	0.121	0.043
**Colorectal**	0.254	0.078	0.078	0.160	0.195	0.116	0.100
**Pancreatic**	0.162	0.060	0.055	0.102	0.140	0.078	0.043
**Melanoma**	0.174	0.078	0.074	0.139	0.164	0.071	0.115

Supplementary Table 3: Standard deviations of either MPAS or actual mean viability across cell lines treated with either MAPK or PI3K/AKT inhibitors.

Supplementary Table 4

Cobimetinib	Gene name	chromosome location	shortlist coefficient
	THG1L	chr5:157158323-157166772	-0.0148
	LINC00460	chr13:107028911-107030140	-0.0114
	TIMP1	chrX:47441690-47446190	-0.00705
	FERMT1	chr20:6055492-6104191	-0.00589
	*PHLDA1	chr12:76419227-76425556	-0.00343
	S100A4	chr1:153516095-153518282	-0.00237
	PLEK2	chr14:67853700-67878828	-0.000503
**Trametinib**	**Gene name**	**chromosome location**	**shortlist coefficient**
	THG1L	chr5:157158323-157166772	-0.025143138
	*PHLDA1	chr12:76419227-76425556	-0.018512537
	PLEK2	chr14:67853700-67878828	-0.012443006
	TIMP1	chrX:47441690-47446190	-0.00995946
	LINC00460	chr13:107028911-107030140	-0.009487112
	FERMT1	chr20:6055492-6104191	-0.004975383
	S100A4	chr1:153516095-153518282	-0.003213673

Supplementary Table 4: Short-list of gene clusters identified by the E-Net model as being predictive of MAPK inhibitor (cobimetinib and trametinib) sensitivity. *PHLDA group also included SPRY2, SPRY4, DUSP6, CCND1 and EPHA2

Supplementary Table 5

Gene	Correlation coefficient
PHLDA1	1
FOSL1	0.687
EPHA2	0.651
PXN	0.633
PHLDA2	0.63
CCND1	0.62
CD97	0.617
ADORA2B	0.611
IER3	0.611
ITGA2	0.61
S100A11	0.61
LOC100216001	0.609
LOC100294307	0.605
SPATS2L	0.605
LOC100653318	0.605
LOC100652851	0.604
C1orf170	0.603
PLAUR	0.592
ANXA2	0.59
LOC100506484	0.589
SPRY4	0.586
YAP1	0.585
TIMP1	0.584
ZFP36L1	0.584
SPRED1	0.581
AHNAK	0.58
DNMBP	0.58
RHOC	0.579
PLEKHN1	0.578
DUSP6	0.577
DCBLD2	0.574
LIF	0.572
RPSAP52	0.57

TNFRSF12A	0.569
FXYD5	0.568
TGFBR2	0.567
NT5E	0.567
FHL2	0.566
ANXA1	0.565
STEAP1	0.563
HRH1	0.561
TRIP10	0.556
CMTM7	0.555
CLIC1	0.554
RIN1	0.554
ELK3	0.554
LOC100506377	0.553
SPRY2	0.553
SP100	0.552
CDCP1	0.549
RALB	0.549
MYOF	0.549
CSF2	0.548
SMAGP	0.548
TNFRSF10B	0.547
SMAD3	0.545
LOC541471	0.544
TMSB10	0.544
LOC100653218	0.544
EMP1	0.544
CNN2	0.544
LOC100287314	0.544
TMBIM1	0.541
LOC100506649	0.54
S100A16	0.54
NPAS2	0.539
LOC100129940	0.538

LMNA	0.538
S100A10	0.537
CAPN2	0.537
ADAMTSL5	0.536
AREGB	0.535
SH3TC2	0.535
ERRFI1	0.532
LINC00460	0.53
TUBA1C	0.529
ITGA3	0.528
PLAU	0.527
TM4SF1	0.526
MIR221	0.526
MIR614	0.525
CD9	0.524
MYEOV	0.522
RNF149	0.521
CIB1	0.521
LOC100652804	0.52
LOC100653288	0.52
HMGA2	0.519
CTSB	0.518
S100A6	0.517
GPRC5A	0.517
RUNX1	0.517
CAV2	0.516
GSTO1	0.516
PTPN12	0.515
CATSPER1	0.515
LOC100508196	0.514
SERTAD1	0.513
ITGA6	0.513
C10orf55	0.511
CASP8	0.511

F2RL1	0.511	
ITGB5	0.51	
PLIN3	0.51	
BAG3	0.51	
EHBP1L1	0.509	
IRAK2	0.508	
LOC389602	0.508	
SOX9	0.507	
REST	0.507	
ANXA2P2	0.506	
SFXN3	0.506	
SLC16A3	0.505	
VPS37B	0.505	
AREG	0.504	
EPHB4	0.503	
LRRC8E	0.503	
ARL14	0.503	
FERMT1	0.502	
IGF2BP2	0.502	
ANXA2P1	0.502	
RPS6KA4	0.502	

Supplementary Table 5: Genes with expression that correlates with the PHLDA1 gene cluster identified by the E-Net model.

*Genes in larger bold italic font are known MAPK target genes and are included in MPAS

Supplementary Table 6

Cell line	Individual MAPK gene expression (nanostring counts)										MPAS	predicted (E-Net)		actual mean viability		actual GI50		KRAS-mut
	DUSP4	DUSP6	SPRY2	SPRY4	CCND1	ETV4	ETV5	EPHA2	EPHA4	PHLDA1		cobi-metinib	tra-metinib	cobi-metinib	tra-metinib	cobi-metinib	tra-metinib	
**A-427**	-0.32	5.71	5.34	3.85	7.27	6.19	5.61	4.72	-4.68	7.33	-0.411	0.77	0.68	0.47	0.27	0.04	0.0002	1
**Calu-1**	2.06	3.29	3.93	3.57	8.21	4.00	4.20	6.79	1.98	5.79	-0.414	0.73	0.63	0.82	0.73	0.27	0.05	1
**Calu-6**	6.09	7.72	6.89	6.63	7.32	5.66	6.31	4.51	3.90	6.91	-0.015	0.64	0.51	0.61	0.44	0.2	0.0003	1
**COLO 699**	4.07	3.91	3.07	-0.55	6.41	0.37	2.50	3.65	2.00	4.95	-0.624	0.80	0.77	0.87	0.9	10	10	0
**EBC-1**	2.05	5.88	3.82	2.23	6.51	4.46	4.47	6.11	-6.35	7.68	-0.323	0.69	0.55	0.45	0.16	0.2	0.001	0
**HCC193**	2.77	3.19	3.00	0.39	7.35	3.51	3.90	5.59	0.01	5.71	-0.598	0.76	0.66	0.95	0.82	7	10	0
**HCC2270**	3.53	4.07	4.07	2.12	8.37	3.03	2.15	7.37	2.42	5.19	-0.602	0.77	0.68	0.82	0.83	10	10	0
**HCC2935**	4.15	6.38	4.38	3.10	8.98	4.94	4.14	5.69	3.44	4.62	-0.485	0.77	0.73	0.82	0.77	0.4	0.001	0
**HCC366**	4.52	5.14	5.17	3.95	7.72	4.99	5.11	7.38	-1.37	6.39	-0.288	0.72	0.62	0.91	0.95	10	10	0
**LOU-NH91**	2.22	0.62	1.87	0.84	5.51	1.68	2.68	6.38	3.19	4.98	-0.817	0.75	0.69	0.89	0.84	0.5	10	0
**NCI-H1373**	4.38	6.67	5.06	3.45	7.08	4.03	4.77	6.86	-1.44	7.08	-0.309	0.71	0.61	0.77	0.67	0.74	0.033	1
**NCI-H1395**	4.56	2.14	2.87	2.49	7.91	1.66	1.77	2.02	-0.40	2.94	-0.589	0.79	0.76	0.92	0.77	0.75	0.03	0
**NCI-H1435**	3.87	6.49	3.27	3.39	7.07	6.30	2.67	6.26	-5.92	4.91	-0.554	0.67	0.61	0.83	0.93	0.09	10	0
**NCI-H1568**	5.12	1.43	1.80	-2.30	4.49	0.17	0.81	2.91	-2.20	2.80	-1.457	0.80	0.77	1.02	0.85	10	10	0
**NCI-H1623**	6.07	1.52	4.95	0.92	6.88	4.60	3.14	4.73	3.10	4.69	-0.474	0.82	0.79	0.92	0.5	0.5	0.001	0
**NCI-H1648**	2.98	4.89	3.49	2.86	7.00	5.24	3.24	7.30	0.04	5.55	-0.372	0.74	0.65	0.53	0.55	0.39	0.002	0
**NCI-H1650**	1.09	-0.13	2.60	0.81	5.83	1.43	2.23	6.18	-0.02	3.94	-0.673	0.78	0.70	1.02	0.9	10	10	0
**NCI-H1693**	1.48	4.76	3.59	3.30	7.46	1.07	3.53	5.91	-1.48	4.14	-0.617	0.76	0.70	0.67	0.75	0.14	0.1	0
**NCI-H1734**	5.09	6.90	4.29	2.18	6.28	5.23	4.63	5.46	1.81	4.40	-0.489	0.79	0.75	0.66	0.71	0.13	0.1	1
**NCI-H1770**	-2.31	-4.08	-3.28	-2.11	-2.10	-1.42	-1.36	3.61	-0.98	1.33	-2.580	0.90	0.87	1.16	0.98	10	10	0
**NCI-H1781**	3.67	6.20	4.30	3.96	5.53	5.81	3.83	5.68	2.21	3.91	-0.739	0.83	0.80	1.00	0.73	0.4	0.08	0
**NCI-H1792**	3.31	4.19	4.48	2.28	6.43	5.10	3.95	4.85	1.12	5.70	-0.637	0.72	0.59	0.79	0.29	0.5	0.0001	1
**NCI-H1793**	3.84	0.97	2.77	-0.10	6.67	1.75	3.88	3.96	-1.43	2.28	-0.812	0.77	0.69	0.86	0.81	10	10	0
**NCI-H1915**	-0.23	4.90	4.37	4.11	7.49	4.58	4.50	5.80	3.08	6.83	-0.197	0.74	0.62	0.80	0.19	0.21	0.0001	0
**NCI-H1975**	3.27	2.87	2.54	2.18	7.24	3.10	3.07	5.47	1.09	5.01	-0.562	0.69	0.58	0.82	0.78	0.14	0.001	0
**NCI-H2030**	4.25	3.14	3.82	3.66	5.42	4.11	4.42	5.62	-4.26	5.13	-0.652	0.77	0.67	0.70	0.6	0.34	0.002	1
**NCI-H2073**	7.28	4.73	3.23	4.62	3.71	3.55	3.92	7.18	-3.75	6.11	-0.624	0.73	0.63	0.75	0.62	0.32	0.001	0
**NCI-H2126**	4.94	3.56	3.76	1.64	7.34	3.90	1.40	6.27	0.29	5.55	-0.555	0.71	0.62	0.72	0.73	0.8	10	0
**NCI-H2172**	3.09	-2.30	2.50	0.32	3.17	2.59	2.19	3.45	-2.35	3.05	-0.666	0.81	0.78	0.97	0.76	10	1	0
**NCI-H2596**	0.26	1.76	1.92	1.65	7.09	2.81	2.83	6.23	2.79	3.60	-0.565	0.79	0.75	0.86	0.8	2.1	8.8	0
**NCI-H2795**	3.95	1.52	4.33	2.18	6.39	4.66	3.58	5.46	4.64	4.97	-0.287	0.78	0.73	0.53	0.35	0.1	0.001	0
**NCI-H596**	2.07	2.17	1.79	1.30	5.47	0.31	3.04	5.11	2.40	3.75	-1.258	0.80	0.76	1.00	0.84	10	10	0
**NCI-H810**	0.04	0.65	2.24	-1.58	-1.18	3.48	2.42	-1.61	5.21	0.44	-0.783	0.88	0.86	0.93	0.89	10	10	0
**NCI-H820**	0.62	5.22	3.16	2.18	6.83	3.61	3.83	6.50	-0.75	5.11	-0.381	0.74	0.63	0.76	0.64	0.16	0.06	0
**NCI-H920**	3.49	5.18	1.83	1.71	7.35	3.45	2.61	5.73	-1.31	5.67	-0.578	0.77	0.67	0.97	0.81	0.32	0.001	0
**SW 900**	3.58	7.17	5.25	4.30	7.93	3.94	4.30	6.16	2.93	7.41	-0.519	0.77	0.69	0.72	0.57	0.67	0.02	1

Supplementary Table 6: Gene expression, KRAS mutation status and predicted/actual mean viabilities to both trametinib and cobimetinib for cell lines in NSCLC validation set

Supplementary Table 7

Study	Figure ID	Median	Max	Min
COBRIM	3	0.08	1.33	-2.26
mCRC	4B	-0.13	4.09	-1.90
AVANT	4C	1.10	2.96	-1.27
TCGA (Adrenal)	5A	-0.62	2.55	-4.69
METABRIC (HER2+)	5Civ	0.14	4.51	-4.47

Supplementary Table 7: Median, maximum and minimum MPAS values for each clinical study.

Supplementary Table 8

	Vendor
Melanoma (n=50)	Folio Biosciences, LLC
	Asterand Bioscience
	ProteoGenex Inc.
	The MT group Inc.
CRC (n=50)	The MT group Inc.
	Tristar Technology ventures
Lung (n=100)	Tristar Technology ventures
	The MT group Inc.
	Cureline, Inc
	ClinPath advisors

Supplementary Table 8: List of vendors from which melanoma, crc and lung patient samples were procured.

Supplementary Table 9

Gene name	Role on panel
**DUSP6**	**MAPK target**
**SPRY2**	**MAPK target**
**CCND1**	**MAPK target**
**DUSP4**	**MAPK target**
**SPRY4**	**MAPK target**
**ETV4**	**MAPK target**
**ETV5**	**MAPK target**
**EPHA2**	**MAPK target**
**EPHA4**	**MAPK target**
**PHLDA1**	**MAPK target**
CLTC	HK genes
GUSB	HK genes
HPRT1	HK genes
PGK1	HK genes
SDHA	HK genes

Supplementary Table 9: Genes included in the custom MAPK-specific nanostring panel.

Supplementary Table 10

		predicted values of mean viability		actual mean viability	
**Cell line name**	**primary tissue**	**cobimetinib**	**trametinib**	**cobimetinib**	**trametinib**
**LS-174T**	Colon	0.673	0.533	0.691	0.545
**SW 48**	Colon	0.650	0.535	0.615	0.421
**OUMS-23**	Colon	0.780	0.748	0.826	0.862
**COLO 201**	Colon	0.527	0.407	0.279	0.172
**COLO 320**	Colon	0.854	0.849	0.934	1.000
**DLD-1**	Colon	0.706	0.587	0.801	0.682
**CL-34**	Colon	0.636	0.520	0.623	0.532
**SW 1463**	Colon	0.642	0.527	0.633	0.510
**LS-174T**	Colon	0.672	0.535	0.593	0.429
**SW 480**	Colon	0.664	0.543	0.626	0.490
**SK-CO-1**	Colon	0.626	0.496	0.492	0.325
**DLD-1**	Colon	0.665	0.558	0.750	0.608
**RKO**	Colon	0.614	0.514	0.479	0.442
**COLO 741**	Colon	0.764	0.690	0.671	0.536
**Caco-2**	Colon	0.779	0.675	0.865	0.748
**LS-174T**	Colon	0.670	0.537	0.754	0.557
**HCT 116**	Colon	0.677	0.530	0.892	0.836
**HT-29**	Colon	0.605	0.504	0.374	0.305
**LoVo**	Colon	0.670	0.522	0.613	0.441
**COLO 201**	Colon	0.556	0.462	0.360	0.302
**HCA-7**	Colon	0.709	0.592	0.694	0.465
**CL-11**	Colon	0.646	0.480	0.528	0.372
**SW 620**	Colon	0.643	0.525	0.627	0.424
**KM-12**	Colon	0.667	0.547	0.859	0.802
**HT-55**	Colon	0.691	0.572	0.636	0.573
**HT-29**	Colon	0.617	0.508	0.384	0.244
**T84**	Colon	0.655	0.506	0.745	0.560
**SW 403**	Colon	0.639	0.530	0.685	0.519
**SW 948**	Colon	0.641	0.501	0.651	0.556
**C170**	Colon	0.661	0.529	0.623	0.460
**COLO 678**	Colon	0.699	0.606	0.726	0.647
**CX-1**	Colon	0.597	0.453	0.509	0.293
**MDST8**	Colon	0.675	0.591	0.723	0.642
**Caco-2**	Colon	0.777	0.683	0.770	0.696
**COLO 320**	Colon	0.869	0.854	0.962	1.001
**HCC1263**	Colon	0.710	0.627	0.746	0.651
**SNU-C1**	Colon	0.720	0.618	0.729	0.593
**RKO**	Colon	0.644	0.532	0.569	0.450
**HCT 116**	Colon	0.666	0.523	0.512	0.277
**HT-115**	Colon	0.714	0.576	0.678	0.557
**GP2d**	Colon	0.736	0.658	0.731	0.623
**CaR-1**	Colon	0.709	0.608	0.736	0.675
**CW-2**	Colon	0.718	0.621	0.845	0.784
**RCM-1**	Colon	0.651	0.532	0.732	0.575
**NCI-H358**	Lung	0.716	0.606	0.665	0.491
**NCI-H292**	Lung	0.734	0.654	0.503	0.354
**NCI-H522**	Lung	0.825	0.812	0.823	0.854
**NCI-H650**	Lung	0.664	0.543	0.742	0.603
**NCI-H23**	Lung	0.728	0.643	0.563	0.388
**NCI-H520**	Lung	0.814	0.747	0.861	0.792
**NCI-H647**	Lung	0.752	0.682	1.021	0.962
**NCI-H1437**	Lung	0.697	0.601	0.529	0.389
**HCC827**	Lung	0.728	0.604	0.713	0.567
**ABC-1**	Lung	0.781	0.716	0.842	0.764
**RERF-LC-KJ**	Lung	0.757	0.684	0.770	0.748
**Calu-3**	Lung	0.724	0.619	0.806	0.772
**NCI-H460**	Lung	0.713	0.611	0.719	0.629
**NCI-H1299**	Lung	0.759	0.654	0.655	0.553
**NCI-H226**	Lung	0.755	0.701	0.756	0.689
**NCI-H1944**	Lung	0.741	0.656	0.722	0.614
**NCI-H2106**	Lung	0.892	0.892	0.991	0.994

**SW 1573**	Lung	0.718	0.609	0.813	0.740
**SK-MES-1**	Lung	0.702	0.601	0.682	0.526
**HCC-15**	Lung	0.671	0.548	0.597	0.494
**NCI-H2122**	Lung	0.696	0.570	0.636	0.439
**NCI-H441**	Lung	0.734	0.625	0.793	0.727
**A549**	Lung	0.688	0.573	0.541	0.400
**NCI-H1703**	Lung	0.765	0.740	0.866	0.935
**NCI-H1666**	Lung	0.662	0.548	0.637	0.559
**NCI-H82**	Lung	0.908	0.919	0.944	1.037
**LXFL529**	Lung	0.829	0.756	0.863	0.730
**NCI-H2171**	Lung	0.911	0.908	0.948	0.997
**NCI-N417**	Lung	0.887	0.860	1.025	1.010
**NCI-H2405**	Lung	0.743	0.670	0.360	0.296
**NCI-H661**	Lung	0.754	0.684	0.753	0.728
**NCI-H1155**	Lung	0.844	0.827	1.013	1.029
**RERF-LC-MS**	Lung	0.710	0.616	0.658	0.534
**HOP-62**	Lung	0.704	0.618	0.761	0.658
**NCI-H1838**	Lung	0.737	0.646	0.856	0.860
**NCI-H2009**	Lung	0.724	0.619	0.575	0.472
**VMRC-LCD**	Lung	0.849	0.821	0.892	0.943
**H322T**	Lung	0.775	0.677	0.724	0.610
**RERF-LC-OK**	Lung	0.739	0.674	0.835	0.759
**NCI-H727**	Lung	0.681	0.594	0.479	0.331
**NCI-H1651**	Lung	0.818	0.766	0.890	0.837
**KNS-62**	Lung	0.710	0.588	0.644	0.488
**HOP-92**	Lung	0.701	0.590	0.633	0.450
**NCI-H446**	Lung	0.855	0.834	0.873	0.783
**NCI-H322T**	Lung	0.737	0.609	0.759	0.583
**HCC4006**	Lung	0.747	0.655	0.783	0.653
**SU-DHL-10**	Lung	0.787	0.704	0.925	0.848
**NCI-H1355**	Lung	0.727	0.641	0.739	0.740
**NCI-H187**	Lung	0.921	0.938	1.032	1.030
**NCI-H196**	Lung	0.817	0.757	0.916	0.842
**NCI-H2170**	Lung	0.822	0.804	1.054	1.106
**NCI-H146**	Lung	0.899	0.907	0.913	0.934
**NCI-H889**	Lung	0.881	0.886	0.926	0.956
**ChaGo-K-1**	Lung	0.818	0.796	0.917	0.938
**NCI-H2110**	Lung	0.740	0.649	0.892	0.823
**HCC515**	Lung	0.684	0.555	0.698	0.618
**HCC1171**	Lung	0.734	0.634	0.680	0.575
**HCC4017**	Lung	0.708	0.578	0.714	0.604
**NCI-H2228**	Lung	0.716	0.611	0.842	0.726
**DV-90**	Lung	0.693	0.595	0.621	0.513
**HCC44**	Lung	0.686	0.569	0.607	0.421
**HCC78**	Lung	0.734	0.646	0.794	0.748
**LCLC-103H**	Lung	0.776	0.726	0.829	0.955
**BEN**	Lung	0.832	0.773	0.917	0.849
**CAL-12T**	Lung	0.677	0.550	0.628	0.548
**EPLC-272H**	Lung	0.744	0.658	0.783	0.726
**LCLC-97TM1**	Lung	0.688	0.557	0.702	0.488
**LXF-289**	Lung	0.752	0.643	0.687	0.576
**SCLC-21H**	Lung	0.917	0.925	0.912	0.985
**SCLC-22H**	Lung	0.874	0.873	0.926	0.972
**NCI-H1339**	Lung	0.754	0.678	0.814	0.703
**NCI-H1930**	Lung	0.942	0.968	1.081	1.137
**NCI-H23**	Lung	0.757	0.663	0.778	0.645
**HCC2885**	Lung	0.763	0.666	0.834	0.740
**UMC-11**	Lung	0.851	0.873	0.882	0.937
**COR-L26**	Lung	0.739	0.646	0.753	0.602
**COR-L279**	Lung	0.882	0.869	0.979	1.019
**COR-L47**	Lung	0.901	0.916	1.003	1.028
**COR-L88**	Lung	0.894	0.903	0.915	0.925
**MOR**	Lung	0.751	0.690	0.876	0.837

**HCC2279**	Lung	0.755	0.675	0.842	0.817
**NCI-H1581**	Lung	0.814	0.810	0.740	0.681
**NCI-H1869**	Lung	0.741	0.656	0.751	0.701
**NCI-H2023**	Lung	0.759	0.674	0.742	0.698
**NCI-H2135**	Lung	0.680	0.513	0.588	0.308
**NCI-H2347**	Lung	0.699	0.556	0.627	0.463
**HCC364**	Lung	0.658	0.500	0.380	0.146
**HCC4011**	Lung	0.764	0.659	0.836	0.757
**HCC2302**	Lung	0.764	0.699	0.796	0.740
**DMS 273**	Lung	0.840	0.820	0.904	0.990
**HCC4019**	Lung	0.749	0.677	0.755	0.659
**HCC1534**	Lung	0.797	0.732	0.882	0.780
**NCI-H2461**	Lung	0.776	0.725	0.780	0.699
**NCI-H2052**	Lung	0.745	0.700	0.864	1.030
**HCC461**	Lung	0.714	0.612	0.735	0.583
**NCI-H2373**	Lung	0.737	0.677	0.759	0.702
**NCI-H2452**	Lung	0.754	0.671	0.835	0.748
**NCI-H28**	Lung	0.780	0.732	0.925	0.900
**NCI-H2595**	Lung	0.783	0.745	0.800	0.814
**NCI-H2369**	Lung	0.774	0.703	0.855	0.823
**NCI-H2803**	Lung	0.779	0.735	0.781	0.721
**NCI-H2804**	Lung	0.781	0.744	0.866	0.876
**NCI-H2722**	Lung	0.779	0.707	0.836	0.769
**NCI-H2810**	Lung	0.734	0.656	0.635	0.533
**NCI-H2731**	Lung	0.747	0.672	0.853	0.816
**HPAC**	Pancreas	0.610	0.491	0.540	0.398
**BxPC-3**	Pancreas	0.674	0.560	0.742	0.632
**SW 1990**	Pancreas	0.675	0.575	0.708	0.621
**MIA Paca-2**	Pancreas	0.697	0.595	0.584	0.447
**HUP-T3**	Pancreas	0.708	0.581	0.672	0.476
**PANC-1**	Pancreas	0.725	0.624	0.786	0.725
**PSN1**	Pancreas	0.622	0.478	0.422	0.194
**KP4**	Pancreas	0.696	0.599	0.697	0.625
**SU.86.86**	Pancreas	0.723	0.615	0.725	0.627
**Capan-2**	Pancreas	0.648	0.524	0.607	0.462
**CFPAC-1**	Pancreas	0.694	0.591	0.707	0.690
**Capan-1**	Pancreas	0.693	0.568	0.795	0.669
**HPAF-II**	Pancreas	0.623	0.455	0.553	0.343
**AsPC-1**	Pancreas	0.603	0.458	0.611	0.479
**Hs 766T**	Pancreas	0.674	0.559	0.512	0.344
**Panc 04.03**	Pancreas	0.649	0.537	0.603	0.460
**Panc 03.27**	Pancreas	0.663	0.543	0.660	0.523
**Panc 05.04**	Pancreas	0.649	0.524	0.563	0.465
**Panc 02.03**	Pancreas	0.607	0.464	0.447	0.257
**PA-TU-8988T**	Pancreas	0.738	0.655	0.713	0.708
**PA-TU-8902**	Pancreas	0.643	0.504	0.638	0.446
**HUP-T4**	Pancreas	0.696	0.601	0.597	0.480
**KLM-1**	Pancreas	0.725	0.624	0.782	0.627
**KP-3**	Pancreas	0.722	0.636	0.811	0.772
**PK-45H**	Pancreas	0.702	0.601	0.506	0.366
**PK-59**	Pancreas	0.645	0.516	0.678	0.552
**PK-8**	Pancreas	0.661	0.536	0.593	0.399
**QGP-1**	Pancreas	0.804	0.743	0.755	0.706
**DAN-G**	Pancreas	0.713	0.627	0.821	0.678
**KCI-MOH1**	Pancreas	0.623	0.501	0.563	0.394
**PK-1**	Pancreas	0.647	0.530	0.579	0.441
**PK-45P**	Pancreas	0.675	0.568	0.632	0.501
**PA-TU-8988S**	Pancreas	0.682	0.568	0.668	0.512
**SUIT-2**	Pancreas	0.674	0.557	0.580	0.362
**TCC-PAN2**	Pancreas	0.661	0.530	0.675	0.601
**YAPC**	Pancreas	0.672	0.567	0.780	0.718

Supplementary Table 10: Predicted and actual mean viabilities in response to either trametinib or cobimetinib for cell lines included in the E-Net training set .

Supplementary Table 11

CC/IM	MPAS	PFS (Months)
Cell Cycle	0.33	2.76
Cell Cycle	-0.16	3.32
Cell Cycle	0.05	7.72
Cell Cycle	0.16	1.84
Cell Cycle	-0.25	2.99
Cell Cycle	-0.95	0.10
Cell Cycle	0.05	6.54
Cell Cycle	0.45	3.45
Cell Cycle	1.33	7.16
Cell Cycle	0.15	3.45
Cell Cycle	0.80	5.52
Cell Cycle	0.64	3.48
Cell Cycle	0.24	11.07
Cell Cycle	0.33	5.55
Cell Cycle	0.45	5.55
Cell Cycle	0.36	16.59
Cell Cycle	1.14	12.91
Cell Cycle	0.25	2.76
Cell Cycle	0.00	7.46
Cell Cycle	0.74	15.18
Cell Cycle	1.21	12.98
Cell Cycle	0.15	0.03
Cell Cycle	0.78	11.04
Cell Cycle	0.08	3.65
Cell Cycle	0.52	2.83
Cell Cycle	0.54	3.52
Cell Cycle	0.19	2.04
Cell Cycle	0.51	3.94
Cell Cycle	-0.23	5.29
Cell Cycle	1.30	14.65
Cell Cycle	0.34	14.98
Cell Cycle	-0.42	7.39
Cell Cycle	0.34	2.76
Cell Cycle	0.12	13.67
Cell Cycle	-0.26	5.59
Cell Cycle	0.63	9.46
Cell Cycle	-0.01	9.13
Cell Cycle	0.57	1.74
Cell Cycle	0.66	3.48
Cell Cycle	-0.32	1.87
Cell Cycle	0.96	5.13
Cell Cycle	-0.29	3.58
Cell Cycle	-0.05	1.84
Cell Cycle	0.49	5.52
Cell Cycle	0.64	3.58
Cell Cycle	0.26	7.62
Cell Cycle	-0.06	7.69
Immune	0.89	16.43
Immune	-0.11	5.55
Immune	-0.87	4.50
Immune	-0.34	11.10
Immune	-0.35	15.01
Immune	-0.26	18.63
Immune	-0.49	7.82
Immune	-0.06	1.87
Immune	-0.09	5.78
Immune	0.18	18.40
Immune	0.54	18.60
Immune	-0.66	18.17
Immune	-0.23	3.81
Immune	0.11	16.85

Immune	-0.43	18.37
Immune	-0.15	16.46
Immune	0.02	11.50
Immune	-0.69	3.71
Immune	-0.59	15.70
Immune	0.79	7.23
Immune	0.50	5.75
Immune	0.13	16.85
Immune	-0.08	7.33
Immune	-0.32	7.69
Immune	-1.87	6.51
Immune	0.28	13.86
Immune	-0.12	6.21
Immune	0.05	9.13
Immune	0.18	3.42
Immune	0.16	0.03
Immune	0.20	13.04
Immune	-0.20	5.59
Immune	0.00	13.14
Immune	0.14	13.80
Immune	-0.15	12.88
Immune	-0.58	8.97
Immune	-0.29	1.91
Immune	-0.59	5.55
Immune	0.42	7.46
Immune	-0.64	1.71
Immune	-0.52	3.48
Immune	-0.57	1.77
Immune	-0.55	13.14
Immune	0.43	13.04
Immune	0.12	13.01
Immune	-1.58	1.84
Immune	-0.78	9.33
Immune	-0.12	7.00
Immune	-0.01	4.11
Immune	0.54	1.87
Immune	-2.26	7.29
Immune	0.08	2.20

Supplementary Table 11: MPAS and PFS of patients included in the vemurafenib arm of the CoBrim trial.

Supplementary Table 12

Individual MAPK gene expression (nanostring counts)										MPAS	BRAFmutation	KRASmutation	DeathCensor	OSyear
CCND1	DUSP4	DUSP6	EPHA2	EPHA4	ETV4	ETV5	PHLDA1	SPRY2	SPRY4					
0.71	1.67	1.58	1.18	1.50	0.68	1.32	2.12	0.42	1.07	3.87	MND	MUT	'Censored'	1.5
-1.02	-0.22	-0.43	-0.66	-1.10	0.18	-0.41	-0.12	1.01	-0.60	-1.07	MND	MUT	'Death'	5.08
0.04	0.90	0.55	0.71	1.13	-0.29	0.34	0.52	0.48	-0.01	1.38	MND	MUT	'Censored'	5.09
-0.29	-0.43	-0.68	-0.08	-0.86	-0.89	-0.75	-0.40	0.20	-0.24	-1.40	<undefined>	<undefined>	'Censored'	0.58
-2.11	-0.87	-1.44	-2.61	-2.25	-1.05	-1.55	-1.98	-1.17	-1.97	-5.38	Unknown	MUT	'Censored'	6.42
-0.32	0.45	0.29	0.07	0.85	1.04	0.20	-0.08	1.59	0.42	1.42	MND	MUT	'Death'	4.67
0.35	2.30	1.47	1.07	0.63	1.22	1.30	0.88	0.57	0.77	3.34	MND	MUT	'Death'	2.33
-0.08	0.66	0.14	0.61	0.89	-0.36	-0.44	0.29	0.80	0.28	0.89	MND	MND	'Death'	2.92
1.39	0.31	1.10	1.04	0.74	0.99	1.26	0.65	0.26	0.60	2.63	MND	MND	'Censored'	4.67
1.01	-0.19	0.49	-0.09	-0.23	0.08	0.04	0.20	-0.01	0.53	0.58	MND	MND	'Death'	3.83
-0.47	-0.57	-0.70	-0.37	-0.40	-0.83	-0.39	0.93	0.13	-0.30	-0.94	MND	MUT	'Death'	2.41
-0.20	0.89	-0.36	-1.49	0.60	-0.76	-0.61	0.14	-0.73	-1.22	-1.18	MND	MUT	'Death'	0.59
-4.16	-1.56	-2.87	-2.79	-1.61	-3.29	-3.15	-3.17	-3.17	-3.28	-9.19	MND	MUT	'Censored'	1.33
0.10	0.47	0.58	1.02	1.22	0.36	0.08	0.01	0.03	0.54	1.40	MND	MUT	'Death'	4.92
1.23	-0.27	0.43	0.78	0.37	0.85	0.18	0.50	0.43	0.93	1.72	MND	MND	'Death'	9.41
0.05	-2.29	0.21	0.06	-0.49	1.25	0.47	-0.07	1.18	-0.13	0.07	MND	MUT	'Death'	2.83
-0.53	-0.07	-0.34	0.17	-2.25	-0.16	0.31	0.20	-0.74	-1.05	-1.41	MND	MUT	'Death'	2.92
1.31	0.55	0.43	0.66	0.21	0.77	1.27	0.23	0.67	0.23	2.00	MND	MUT	'Death'	1.59
0.08	-0.15	-0.30	-0.28	-0.46	1.04	0.07	-0.97	0.63	-0.10	-0.14	MND	MUT	'Death'	1.42
0.61	0.48	-0.22	0.20	-1.10	0.91	-0.34	-0.45	-0.06	0.27	0.10	MND	MUT	'Death'	3.92
-0.14	-0.36	0.85	-0.36	0.61	0.49	0.72	-0.23	0.23	0.42	0.71	MND	MUT	'Death'	1.66
-0.17	0.24	0.39	0.69	-0.46	1.18	0.13	-0.64	1.67	1.36	1.39	MND	MND	'Death'	2.5
-0.66	-1.39	-1.42	-0.22	0.31	0.08	-0.99	-1.22	-0.12	-0.39	-1.90	MND	MUT	'Death'	7.33
0.03	-0.60	0.14	-0.01	0.59	0.68	-0.06	0.88	0.15	0.57	0.75	MND	MUT	'Death'	4.33
-1.43	-1.19	-1.61	-1.53	-2.25	-1.21	-1.74	-0.81	-0.85	-1.54	-4.48	MND	MUT	'Death'	0.67
-1.86	-1.47	-1.92	-1.88	-1.32	-1.45	-1.55	-1.06	-0.93	-1.41	-4.69	MND	MND	'Death'	1.83
-1.40	-1.25	-1.64	-2.17	-1.50	-0.85	-1.64	-0.93	-1.10	-1.77	-4.50	MND	MUT	'Death'	7.59
-0.41	-0.09	-0.94	-0.22	0.81	-0.99	-1.14	-0.05	-0.52	-0.42	-1.26	MND	MND	'Death'	4.5
1.13	0.22	0.70	0.86	-0.15	1.51	0.27	0.84	0.92	0.83	2.25	MND	MND	'Censored'	8.25
-0.35	-0.87	-0.61	-0.12	-1.61	-0.13	-1.32	-0.10	-0.55	-0.68	-2.01	MND	MND	'Death'	3.59
-1.15	-0.74	-1.13	-0.35	-1.24	-0.82	-0.49	-1.34	-1.34	-1.12	-3.08	MND	MND	'Death'	0.08
-0.20	-0.65	-0.01	-0.03	0.04	-0.42	0.05	-0.18	-0.56	-0.06	-0.64	MND	MND	'Death'	1.84
0.41	0.88	0.55	-0.37	1.19	0.03	-0.17	1.12	0.41	0.41	1.41	MND	MUT	'Death'	0.25
1.27	-0.48	1.20	0.54	-0.77	1.70	0.34	0.32	0.78	0.84	1.82	MND	MND	'Death'	0.33
-1.74	-1.56	-1.79	-1.91	-0.09	-2.32	-1.85	-0.80	-1.33	-1.77	-4.79	MUT	MND	'Death'	0.08
0.29	-0.41	-0.10	0.41	0.97	-1.08	0.52	-0.39	-1.85	-0.09	-0.55	MND	MND	'Death'	0.08
0.63	-1.91	-0.18	-0.42	-1.40	0.90	-0.90	-0.91	0.17	-0.13	-1.31	MND	MND	'Death'	3.17
1.62	0.75	0.26	0.03	0.27	0.96	0.84	1.59	1.24	1.13	2.75	MND	MND	'Death'	1.08
-0.26	-1.91	-1.16	-0.59	-0.86	-0.58	-1.09	-1.00	-1.46	-1.26	-3.22	MND	MND	'Death'	0.84
-0.52	0.22	0.01	0.21	-0.23	0.15	-0.11	-0.12	-0.16	0.02	-0.17	MND	MUT	'Death'	0.92
-0.77	-0.99	-1.55	-0.30	0.27	-1.30	-0.39	-1.11	-0.92	-1.23	-2.62	MND	MND	'Death'	0.42
0.72	0.41	0.93	0.92	-0.18	0.58	0.80	-0.33	-0.35	0.97	1.41	MND	MND	'Censored'	3.5
0.51	0.37	0.68	0.99	1.30	0.14	0.31	0.78	0.91	0.37	2.01	MND	MUT	'Death'	1.58

0.53	0.21	0.06	-0.25	0.24	0.74	0.26	-0.15	0.26	0.17	0.65	MND	MUT	'Censored'	7.25
0.26	0.99	0.91	1.46	1.53	-0.60	-0.25	0.50	-0.53	0.50	1.51	MND	MUT	'Death'	0.17
1.11	1.40	1.65	1.58	1.12	1.10	1.61	2.65	2.26	1.98	5.20	MND	MUT	'Death'	2.5
-1.54	-0.95	-1.81	-1.66	-0.06	-1.75	-1.64	-0.45	-1.26	-1.54	-4.00	MND	MUT	'Death'	1.25
0.66	0.72	-0.19	-0.30	1.03	0.06	-0.32	0.61	0.49	0.03	0.89	MND	MUT	'Censored'	2.67
0.12	0.55	0.66	0.62	-0.20	0.18	0.25	0.23	-0.69	-0.18	0.48	MUT	MND	'Death'	9
0.31	2.26	1.96	1.62	1.19	1.35	2.82	0.64	2.42	2.51	5.40	MND	MUT	'Death'	0.08
1.49	-0.30	0.69	0.02	0.40	0.71	0.88	-0.05	0.73	0.52	1.60	MND	MND	'Censored'	1.67
0.92	0.20	0.37	0.43	0.59	0.36	1.11	0.58	0.06	0.46	1.60	MND	MND	'Death'	0.42
-1.19	-1.04	-1.31	-0.47	-1.50	-0.83	-0.75	-1.71	-1.74	-1.17	-3.70	MND	MND	'Death'	1
0.72	-0.07	0.38	0.21	0.24	0.69	0.47	-0.20	0.44	0.38	1.03	MND	MND	'Death'	5.5
0.71	1.86	0.88	1.65	0.37	0.55	1.40	0.80	0.66	1.38	3.24	MUT	MND	'Death'	0.83
0.58	0.30	1.34	0.69	0.53	1.08	1.42	0.67	1.06	1.07	2.76	MND	MND	'Death'	1.08
0.26	0.12	0.12	0.77	-0.11	-0.13	0.25	0.04	-0.25	0.44	0.48	MND	MND	'Death'	2.25
1.48	2.24	1.97	2.02	2.34	0.25	1.75	2.25	0.92	0.93	5.11	MND	MUT	'Death'	0.08
-1.47	-0.71	-1.02	-1.53	-1.24	-0.79	-0.44	-2.18	0.15	-0.72	-3.15	MND	MUT	'Censored'	5.25
0.98	0.55	1.25	1.33	1.25	1.12	1.50	1.21	0.80	1.53	3.64	MND	MND	'Censored'	0.67
0.08	1.47	0.37	0.49	1.16	-0.46	0.75	0.12	-0.13	-0.11	1.19	MUT	MND	'Death'	2.83
0.31	-0.36	0.06	-0.91	-0.81	0.40	-0.41	1.35	0.35	0.36	0.11	MND	MUT	'Death'	1.34
-0.90	0.36	-1.00	-0.20	0.99	-2.49	-0.08	-1.19	-1.34	-1.29	-2.26	MUT	MND	'Death'	1.25
-0.33	-0.71	0.05	-0.23	-0.60	0.32	-1.09	-0.92	-0.41	-0.64	-1.44	<undefined>	<undefined>	'Censored'	13.08
0.07	0.15	-0.23	-0.34	-0.97	0.05	0.04	-0.29	-0.72	-0.12	-0.75	MND	MND	'Death'	1.34
0.34	-0.43	0.12	0.49	0.04	0.29	-0.64	0.20	0.19	0.34	0.29	MND	MND	'Death'	6.34
0.53	1.09	0.52	-0.61	0.31	-1.34	0.43	1.67	-0.07	0.27	0.88	MND	MUT	'Death'	1.92
-0.42	-0.41	-0.36	-0.35	0.26	-0.47	0.20	-0.10	-1.35	-0.85	-1.23	MND	MUT	'Death'	1.59
-0.60	-0.46	-0.26	-0.45	-0.86	-0.20	-0.13	0.12	-1.01	0.31	-1.12	<undefined>	<undefined>	'Death'	2.92
0.86	0.66	0.67	0.81	0.45	0.69	0.57	0.13	1.28	0.84	2.20	<undefined>	<undefined>	'Censored'	3.25
1.13	1.63	1.25	0.90	0.77	1.10	0.81	0.52	1.41	1.14	3.37	<undefined>	<undefined>	'Death'	1.08
-0.21	-0.60	-0.71	-1.15	-0.18	-0.99	-0.44	-0.81	-0.90	-0.33	-2.00	<undefined>	<undefined>	'Death'	6.25

Supplementary Table 12: Individual MAPK gene expression, MPAS and overall survival of patients with metastatic CRC.

Supplementary Table 13

Treatment	individual MAPK gene expression						MPAS	CSDIED	TTMDIED	mutation status	
	DUSP6	SPRY2	CCND1	ETV4	ETV5	EPHA4				BRAF	KRAS
FOLFOX-4	2.99	3.11	3.12	2.73	2.25	1.59	0.70	0	72.38	WT	WT
FOLFOX-4	2.86	2.84	2.66	2.50	2.32	2.31	0.21	0	72.54	MUT	NA
FOLFOX-4	3.01	2.76	3.54	2.86	2.05	1.99	1.10	0	76.25	WT	NA
FOLFOX-4	3.29	3.01	3.19	2.75	2.10	2.15	1.50	1	61.11	WT	WT
FOLFOX-4	3.09	2.63	3.21	2.21	2.06	2.68	0.60	0	58.84	WT	MUT
FOLFOX-4	2.90	2.67	3.13	2.23	2.08	2.42	0.04	1	58.87	WT	WT
FOLFOX-4	2.90	2.85	3.04	2.82	2.15	1.57	-0.02	0	60.78	WT	WT
FOLFOX-4	3.35	3.00	3.25	2.47	2.12	2.27	1.49	0	48.13	WT	MUT
FOLFOX-4	2.82	2.66	2.99	2.30	2.00	1.85	-1.05	1	56.34	WT	NA
FOLFOX-4	3.13	2.69	3.32	2.61	1.92	1.91	0.21	0	66.23	WT	MUT
FOLFOX-4	3.18	2.88	3.18	2.77	2.08	1.72	0.59	0	81.02	WT	MUT
FOLFOX-4	2.96	2.95	3.52	2.67	1.89	1.95	0.71	0	75.63	WT	WT
FOLFOX-4	3.17	3.15	3.36	3.01	1.92	2.22	1.83	0	61.17	WT	MUT
FOLFOX-4	3.14	2.53	3.33	2.34	2.31	2.29	0.86	0	76.62	WT	MUT
FOLFOX-4	2.72	2.37	3.18	2.06	1.93	2.09	-1.46	0	68.9	WT	NA
FOLFOX-4	2.74	2.49	2.85	1.84	1.72	1.96	-2.55	1	79.34	WT	MUT
FOLFOX-4	3.47	2.72	3.31	2.66	2.13	2.42	1.76	0	76.25	WT	MUT
FOLFOX-4	3.18	2.77	3.42	2.67	2.19	2.71	2.09	0	66.99	WT	WT
FOLFOX-4	3.10	3.02	2.99	2.95	2.03	1.88	0.72	0	78.49	WT	MUT
FOLFOX-4	2.70	2.81	3.02	2.14	1.89	2.16	-0.97	0	78.16	WT	WT
FOLFOX-4	2.87	2.67	3.15	2.72	1.90	2.52	0.38	0	63.18	WT	NA
FOLFOX-4	2.61	2.81	2.82	2.64	1.86	1.71	-1.40	0	67.25	WT	MUT
FOLFOX-4	2.84	2.48	2.97	2.61	2.11	1.91	-0.66	0	79.93	WT	NA
FOLFOX-4	3.14	2.81	3.42	2.98	2.07	1.87	1.20	0	71.33	WT	MUT
FOLFOX-4	2.98	2.77	3.31	2.38	2.18	2.29	0.77	0	84.24	WT	NA
FOLFOX-4	3.33	2.94	3.23	2.65	2.32	2.21	1.88	0	79.01	WT	MUT
FOLFOX-4	3.11	3.03	3.30	2.66	1.70	2.46	0.95	0	81.35	WT	MUT
FOLFOX-4	3.01	3.20	3.25	3.08	2.23	2.04	2.00	1	62.88	WT	MUT
FOLFOX-4	2.91	2.61	3.12	2.28	1.99	2.27	-0.35	1	55.59	WT	MUT
FOLFOX-4	2.83	3.22	3.16	2.80	1.88	2.67	1.39	0	78.46	WT	MUT
FOLFOX-4	3.15	2.73	3.29	2.46	2.26	2.52	1.44	1	61.7	MUT	WT
FOLFOX-4	2.96	2.31	3.13	2.35	2.34	1.97	-0.34	0	0.03	MUT	NA
FOLFOX-4	2.30	2.34	2.44	1.93	1.54	1.41	-4.87	0	82.5	WT	WT
FOLFOX-4	3.01	2.47	3.07	2.70	1.91	1.72	-0.82	0	76.58	WT	MUT
FOLFOX-4	2.98	2.48	3.21	2.83	1.79	1.93	-0.46	0	81.74	WT	WT
FOLFOX-4	2.88	2.70	3.11	2.89	1.88	1.72	-0.42	0	59.37	WT	MUT
FOLFOX-4	2.33	2.38	3.14	2.25	1.90	1.77	-2.25	0	65.05	WT	WT
FOLFOX-4	2.53	2.11	2.79	1.98	1.69	1.82	-3.57	0	80.76	WT	NA
FOLFOX-4	3.05	2.63	3.39	2.34	2.05	2.39	0.60	0	80.79	WT	MUT
FOLFOX-4	2.92	2.71	3.15	2.56	1.86	1.91	-0.47	0	73.36	WT	MUT
FOLFOX-4	3.46	2.83	3.47	2.71	2.30	2.37	2.47	0	68.8	WT	MUT
FOLFOX-4	3.05	2.86	3.49	2.26	2.11	2.76	1.58	0	78	WT	WT
FOLFOX-4	3.15	2.73	3.20	2.27	2.32	2.35	1.01	0	84.11	WT	WT

FOLFOX-4	3.29	3.04	3.12	2.85	2.31	1.79	1.52	0	62.09	WT	MUT
FOLFOX-4	2.95	2.58	3.28	2.32	1.82	2.12	-0.54	0	81.84	WT	WT
FOLFOX-4	2.58	2.46	3.01	2.39	1.99	1.81	-1.62	1	56.8	WT	NA
FOLFOX-4	2.98	2.38	3.13	2.60	2.05	2.09	-0.31	0	72.57	WT	NA
FOLFOX-4	3.09	2.62	3.22	1.59	1.95	1.99	-1.16	0	81.22	WT	NA
FOLFOX-4	2.89	2.65	3.28	2.45	2.05	1.66	-0.48	0	84.27	WT	WT
FOLFOX-4	2.97	2.94	3.28	2.63	2.62	2.04	1.81	0	68.7	WT	WT
FOLFOX-4	2.91	2.71	3.19	2.82	1.88	1.76	-0.27	0	84.01	WT	NA
FOLFOX-4	3.35	2.81	2.95	2.60	2.11	1.97	0.54	0	65.31	WT	MUT
FOLFOX-4	3.16	2.72	3.31	2.60	2.15	2.00	0.78	0	84.11	WT	NA
FOLFOX-4	3.01	2.81	3.23	2.82	1.89	1.89	0.23	0	72.25	WT	WT
FOLFOX-4	2.96	2.75	3.37	2.74	2.04	1.63	0.18	0	64.43	WT	WT
FOLFOX-4	2.90	2.95	3.57	2.57	2.07	2.35	1.40	0	86.28	WT	MUT
FOLFOX-4	3.42	3.02	3.42	2.66	1.72	1.81	0.78	0	84.37	WT	MUT
FOLFOX-4	2.88	2.77	3.20	2.69	1.83	1.81	-0.43	0	63.41	WT	WT
FOLFOX-4	2.96	2.76	2.93	2.50	1.80	2.35	-0.34	0	79.01	WT	NA
FOLFOX-4	3.11	2.68	3.16	2.81	2.10	1.89	0.46	0	62.03	WT	WT
FOLFOX-4	3.02	3.26	3.17	2.94	2.03	1.76	1.08	0	76.42	WT	MUT
FOLFOX-4	2.99	2.59	3.21	2.49	2.21	1.90	0.09	0	67.38	WT	WT
FOLFOX-4	3.04	2.82	3.39	2.83	1.88	1.81	0.42	0	63.15	WT	WT
FOLFOX-4	2.64	2.81	3.01	2.37	1.85	1.76	-1.35	0	82.83	WT	WT
FOLFOX-4	3.12	3.33	3.40	3.01	2.21	2.64	3.16	0	86.01	WT	MUT
FOLFOX-4	3.21	2.77	3.42	2.73	2.02	2.42	1.51	0	72.11	WT	WT
FOLFOX-4	3.09	3.40	2.60	2.82	2.37	2.63	2.12	0	84.63	MUT	NA
FOLFOX-4	2.34	2.45	2.96	2.35	1.60	1.83	-2.79	0	76.65	WT	NA
FOLFOX-4	3.31	2.99	3.33	2.98	2.21	2.01	2.03	0	61.7	WT	MUT
FOLFOX-4	2.73	2.53	3.10	2.23	2.09	2.01	-0.90	1	75.04	WT	WT
FOLFOX-4	3.10	2.71	3.27	2.26	2.33	2.41	1.08	1	72.15	WT	MUT
FOLFOX-4	2.88	2.51	2.83	1.46	1.93	2.56	-1.69	0	60.94	WT	WT
FOLFOX-4	2.97	2.69	2.91	2.56	1.89	2.12	-0.49	0	83.65	WT	WT
FOLFOX-4	2.71	2.62	3.12	2.51	1.80	1.60	-1.50	0	83.29	WT	WT
FOLFOX-4	2.91	2.93	3.14	2.92	1.94	1.68	0.13	0	80.03	WT	NA
FOLFOX-4	3.33	3.07	3.13	2.72	2.26	1.95	1.56	1	30.49	WT	MUT
FOLFOX-4	2.73	2.64	3.02	2.67	1.96	1.93	-0.68	0	84.96	WT	NA
FOLFOX-4	2.92	2.82	3.09	2.60	2.12	2.47	0.81	0	66.83	WT	MUT
FOLFOX-4	2.69	2.23	2.81	1.90	2.09	2.08	-2.16	0	78.95	WT	NA
FOLFOX-4	2.78	2.66	3.28	2.51	1.83	2.13	-0.41	0	78.39	WT	NA
FOLFOX-4	2.82	2.65	3.11	2.81	2.10	1.93	0.01	0	83.12	MUT	NA
FOLFOX-4	3.12	2.70	3.05	1.92	2.08	2.51	0.01	0	30.09	WT	WT
FOLFOX-4	3.43	2.82	3.25	3.02	2.12	2.34	2.08	0	79.61	WT	MUT
FOLFOX-4	2.82	3.19	3.32	2.61	2.29	1.95	1.28	0	78	WT	MUT
FOLFOX-4	2.84	2.63	3.22	2.20	1.89	1.80	-1.11	0	85.72	WT	NA
FOLFOX-4	3.06	2.72	3.19	2.71	1.80	2.11	0.08	0	71.85	WT	WT
FOLFOX-4	2.79	2.66	2.92	1.93	1.75	2.08	-1.82	0	60.22	WT	MUT
FOLFOX-4	3.17	2.74	3.02	2.40	1.83	2.50	0.16	0	84.53	WT	MUT
FOLFOX-4	2.95	2.74	3.28	2.81	2.12	1.70	0.34	0	77.31	WT	NA

FOLFOX-4	3.07	2.98	3.22	2.67	2.06	2.16	1.05	0	65.91	WT	WT
FOLFOX-4	2.96	2.42	3.17	2.39	1.92	1.79	-1.07	0	75.01	NA	WT
FOLFOX-4	2.94	2.89	3.43	2.94	1.86	1.84	0.58	0	79.15	WT	NA
FOLFOX-4	3.09	2.68	3.00	2.20	1.74	2.30	-0.70	0	67.19	WT	NA
FOLFOX-4	2.78	2.56	3.16	2.63	1.92	1.89	-0.71	0	66.76	WT	MUT
FOLFOX-4	3.35	2.99	3.09	2.78	2.05	2.28	1.50	0	74.51	WT	MUT
FOLFOX-4	3.32	3.01	3.01	2.58	2.00	1.95	0.64	0	72.21	WT	MUT
FOLFOX-4	2.92	2.86	3.20	2.52	1.94	1.95	-0.04	0	78.19	WT	NA
FOLFOX-4	3.10	2.88	3.56	2.85	1.92	1.88	1.05	0	78.19	WT	WT
FOLFOX-4	2.83	2.57	2.78	2.56	1.87	2.31	-0.87	0	84.63	WT	NA
FOLFOX-4	2.98	2.32	3.34	2.21	2.28	1.82	-0.41	0	66.73	WT	MUT
FOLFOX-4	2.71	2.81	2.44	2.47	1.99	1.83	-1.62	0	49.51	WT	NA
FOLFOX-4	2.40	2.64	2.94	2.76	1.56	1.86	-2.02	0	78.92	WT	WT
FOLFOX-4	2.77	2.55	2.87	1.91	2.05	2.28	-1.29	0	84.11	WT	NA
FOLFOX-4	3.30	2.99	3.28	2.80	2.30	1.99	1.86	0	60.06	WT	NA
FOLFOX-4	2.85	2.85	2.98	2.74	2.16	1.69	-0.12	0	82.2	WT	MUT
FOLFOX-4	3.21	2.85	3.03	2.44	2.07	2.31	0.67	0	72.21	WT	MUT
FOLFOX-4	3.16	2.89	3.11	2.69	1.84	1.93	0.18	0	60.12	WT	WT
FOLFOX-4	2.91	2.70	2.98	2.38	1.94	2.15	-0.52	0	73.66	WT	NA
FOLFOX-4	2.90	2.76	3.05	2.26	1.76	2.38	-0.56	0	66.27	WT	MUT
FOLFOX-4	2.91	3.04	3.32	2.27	1.81	2.52	0.55	0	65.61	MUT	WT
FOLFOX-4	2.67	2.75	2.70	2.32	1.68	1.76	-2.23	0	72.74	WT	NA
FOLFOX-4	2.63	2.81	3.12	1.83	1.83	2.23	-1.32	0	67.78	WT	WT
FOLFOX-4	2.73	2.53	3.00	2.46	1.94	2.03	-1.05	0	86.01	WT	MUT
FOLFOX-4	3.19	2.42	3.19	2.57	2.14	2.04	0.20	1	16.2	WT	NA
FOLFOX-4	2.88	2.52	3.10	2.58	2.08	2.16	-0.16	0	83.88	WT	WT
FOLFOX-4	3.24	2.62	3.28	2.98	1.74	1.89	0.24	0	78.69	WT	WT
FOLFOX-4	3.48	2.89	3.25	2.60	2.11	2.32	1.70	0	48	WT	WT
FOLFOX-4	3.02	2.70	2.73	2.81	2.08	2.05	-0.12	1	43.2	WT	MUT
FOLFOX-4	2.97	2.55	2.88	2.45	1.89	1.83	-1.23	0	82	WT	NA
FOLFOX-4	3.02	2.71	2.81	2.07	2.13	2.47	-0.25	0	79.64	WT	MUT
FOLFOX-4	3.07	2.64	3.18	2.53	1.99	2.20	0.22	0	83.58	WT	WT
FOLFOX-4	2.80	2.74	3.38	2.65	2.05	2.08	0.42	0	85.68	WT	NA
FOLFOX-4	3.32	3.02	3.14	2.51	2.26	2.44	1.85	0	83.84	WT	NA
FOLFOX-4	2.75	2.49	2.91	2.26	1.59	2.00	-2.15	0	79.11	WT	MUT
FOLFOX-4	2.40	2.15	2.68	1.98	1.38	2.10	-4.09	0	79.93	WT	NA
FOLFOX-4	2.67	2.84	3.16	2.76	1.79	2.21	-0.16	0	81.05	WT	MUT
FOLFOX-4	3.17	2.83	3.18	2.57	2.16	2.18	0.96	1	30.13	WT	MUT
FOLFOX-4	2.90	2.59	3.21	2.29	2.13	1.73	-0.62	0	75.07	WT	NA
FOLFOX-4	2.81	2.52	3.09	2.24	1.93	2.14	-0.97	0	67.38	WT	WT
FOLFOX-4	3.01	2.84	3.19	2.73	2.11	2.09	0.78	0	79.7	WT	MUT
FOLFOX-4	2.88	2.67	3.05	2.42	1.98	2.12	-0.43	0	76.42	WT	NA
FOLFOX-4	3.06	2.39	3.05	2.00	2.06	2.39	-0.62	0	84.01	WT	MUT
FOLFOX-4	3.12	2.71	3.26	2.82	1.85	1.83	0.13	0	74.74	WT	MUT
FOLFOX-4	2.61	2.68	3.00	2.64	1.71	2.11	-1.14	0	73	WT	NA
FOLFOX-4	2.71	1.87	3.11	1.86	1.89	1.81	-2.99	0	85.03	MUT	NA

FOLFOX-4	3.31	2.83	2.96	2.61	2.23	2.01	0.82	1	74.84	WT	WT
FOLFOX-4	2.94	2.40	2.92	2.18	2.15	2.39	-0.58	0	78.19	WT	NA
FOLFOX-4	2.68	2.65	2.83	2.49	1.86	1.85	-1.54	0	77.83	WT	WT
FOLFOX-4	3.05	2.58	2.95	2.54	1.88	2.00	-0.66	0	60.68	WT	NA
FOLFOX-4	3.08	2.63	3.22	2.25	2.21	2.23	0.39	0	78.59	WT	MUT
FOLFOX-4	2.80	2.79	3.28	2.73	1.90	1.71	-0.29	0	61.77	WT	NA
FOLFOX-4	2.59	2.63	2.76	2.49	2.01	2.03	-1.30	0	82.5	WT	NA
FOLFOX-4	2.79	2.47	2.56	1.91	1.76	1.95	-2.79	0	80.72	WT	MUT
FOLFOX-4	3.18	2.79	3.15	2.57	2.23	1.98	0.78	0	83.06	WT	WT
FOLFOX-4	3.12	2.75	3.17	2.34	1.70	1.76	-0.88	0	77.34	WT	WT
FOLFOX-4	2.67	2.49	3.04	2.51	1.71	1.32	-2.39	0	68.76	WT	MUT
FOLFOX-4	2.77	2.83	3.19	2.44	1.68	1.84	-1.01	0	83.61	WT	WT
FOLFOX-4	2.81	2.48	3.06	2.46	1.57	1.52	-2.28	0	72.57	WT	NA
FOLFOX-4	3.01	2.90	2.98	2.18	2.14	2.22	0.14	0	83.98	WT	WT
FOLFOX-4	2.91	2.03	3.32	1.71	2.03	1.60	-2.32	0	74.28	MUT	NA
FOLFOX-4	2.91	2.36	3.24	2.17	1.93	1.95	-1.17	0	82.23	MUT	WT
FOLFOX-4	2.85	2.67	3.06	2.23	1.94	1.76	-1.19	0	73.17	WT	WT
FOLFOX-4	3.27	2.85	3.56	2.56	2.00	2.25	1.48	0	66.04	WT	MUT
FOLFOX-4	3.02	2.60	3.13	2.41	2.26	2.41	0.64	0	47.74	WT	MUT
FOLFOX-4	3.06	2.65	3.22	2.51	2.07	2.55	0.84	0	80.62	WT	MUT
FOLFOX-4	3.29	2.88	3.09	2.77	2.03	1.83	0.64	0	60.52	WT	MUT
FOLFOX-4	2.61	2.43	2.81	2.23	1.77	1.68	-2.67	0	76.16	WT	MUT
FOLFOX-4	2.82	2.79	3.62	2.63	1.79	1.84	0.06	0	70.28	WT	WT
FOLFOX-4	2.85	2.78	2.88	2.58	1.75	1.98	-0.99	0	84.37	WT	WT
FOLFOX-4	2.98	2.62	3.03	2.46	1.76	1.66	-1.34	0	62	WT	WT
FOLFOX-4	2.98	2.79	3.13	2.37	2.04	1.99	-0.11	0	68.73	WT	NA
FOLFOX-4	2.80	2.39	2.81	2.16	2.02	2.42	-1.20	1	4.99	WT	NA
FOLFOX-4	3.14	2.35	2.88	2.03	1.88	2.03	-1.58	1	12.85	WT	NA
FOLFOX-4	3.03	2.71	2.74	2.85	1.79	1.86	-0.80	0	87	WT	NA
FOLFOX-4	2.66	3.03	3.00	2.60	1.99	1.46	-0.83	0	60.58	WT	NA
FOLFOX-4	2.82	2.36	3.23	2.53	2.08	1.38	-1.30	0	82.66	WT	WT
FOLFOX-4	3.15	2.85	3.18	2.15	2.04	2.38	0.49	0	76.16	WT	MUT
FOLFOX-4	3.33	2.72	3.30	2.65	2.09	2.31	1.34	0	78.85	WT	MUT
FOLFOX-4	2.83	2.92	3.12	2.54	1.66	2.39	-0.15	0	68.76	WT	MUT
FOLFOX-4	2.71	2.92	3.35	2.36	2.06	2.33	0.51	0	84.83	WT	NA
FOLFOX-4	3.06	2.69	3.21	2.94	2.00	1.84	0.39	0	57.1	WT	MUT
FOLFOX-4	3.47	2.66	3.33	2.68	2.13	2.10	1.35	1	21.95	WT	NA
FOLFOX-4	3.25	2.82	3.42	2.67	2.27	2.06	1.62	0	59.63	WT	MUT
FOLFOX-4	3.35	3.03	3.28	2.47	2.11	2.34	1.66	1	49.74	WT	MUT
FOLFOX-4	2.96	2.84	3.75	2.35	2.06	2.57	1.58	0	73.53	WT	MUT
FOLFOX-4	3.10	3.24	3.06	2.91	2.11	2.53	2.06	0	82.66	WT	MUT
FOLFOX-4	3.15	2.90	3.25	2.58	1.97	2.64	1.37	0	63.64	MUT	NA
FOLFOX-4	3.20	3.03	3.12	2.92	2.08	1.92	1.17	0	76.75	WT	MUT
FOLFOX-4	3.21	3.15	3.17	2.80	2.27	2.10	1.90	0	81.25	WT	MUT
FOLFOX-4	3.09	2.79	2.99	2.61	2.09	2.32	0.61	0	77.9	WT	WT
FOLFOX-4	3.38	2.77	3.13	3.07	2.39	2.16	2.11	0	72.57	MUT	WT

FOLFOX-4	3.14	2.85	3.54	2.62	2.19	2.53	2.05	1	47.28	WT	MUT
FOLFOX-4	2.88	2.78	3.44	2.03	1.97	2.39	0.15	0	59.3	WT	WT
FOLFOX-4	3.24	2.76	3.14	2.72	1.88	1.96	0.27	0	54.31	WT	WT
FOLFOX-4	3.00	3.32	3.05	2.78	1.95	1.83	0.73	0	77.14	WT	MUT
FOLFOX-4	3.35	2.94	3.19	2.54	2.29	2.47	1.97	0	60.98	WT	MUT
FOLFOX-4	2.71	2.79	2.69	1.98	1.68	2.39	-1.78	1	52.07	WT	NA
FOLFOX-4	3.08	2.75	3.15	2.16	1.95	2.24	-0.13	0	76.48	WT	NA
FOLFOX-4	3.22	2.76	3.23	2.10	2.17	2.81	1.24	0	59.63	MUT	WT
FOLFOX-4	3.27	2.78	3.67	2.74	1.90	1.91	1.16	0	57.3	WT	NA
FOLFOX-4	3.14	2.41	3.13	2.19	2.23	2.18	-0.08	1	60.94	WT	MUT
FOLFOX-4	3.43	2.76	3.50	2.54	2.16	2.48	2.06	0	60.29	WT	WT
FOLFOX-4	2.80	2.82	3.31	2.72	1.88	2.44	0.62	0	59.99	WT	WT
FOLFOX-4	3.12	2.67	3.27	2.39	2.20	2.09	0.57	1	40.25	WT	MUT
FOLFOX-4	3.22	2.87	3.27	2.75	2.39	2.43	2.19	0	63.61	WT	MUT
FOLFOX-4	3.20	2.46	3.29	2.10	1.78	2.35	-0.44	0	73.79	WT	WT
FOLFOX-4	2.96	2.64	3.23	2.51	2.02	2.12	0.08	0	75.04	WT	WT
FOLFOX-4	2.98	2.37	2.67	1.52	2.01	2.21	-2.20	0	66.27	WT	MUT
FOLFOX-4	3.05	2.98	3.44	2.63	1.71	2.21	0.67	0	75.37	WT	MUT
FOLFOX-4	3.25	2.81	3.38	2.68	1.94	2.16	1.05	0	59.93	WT	MUT
FOLFOX-4	3.31	2.89	3.11	2.69	2.05	1.86	0.70	0	77.77	WT	NA
FOLFOX-4	3.11	2.81	3.07	2.49	2.12	2.17	0.53	0	65.15	WT	NA
FOLFOX-4	2.84	2.51	2.76	2.08	2.04	1.85	-1.77	0	63.44	WT	NA
FOLFOX-4	2.76	2.73	3.38	2.54	1.93	1.92	-0.21	0	81.15	WT	NA
FOLFOX-4	3.12	2.46	3.50	2.10	2.34	1.97	0.39	0	60.81	NA	WT
FOLFOX-4	2.91	3.04	3.32	2.27	1.81	2.52	0.55	1	35.65	MUT	NA
FOLFOX-4	2.77	2.43	3.00	2.29	1.91	1.68	-1.84	0	77.8	WT	MUT
FOLFOX-4	2.91	3.04	3.32	2.27	1.81	2.52	0.55	0	81.08	WT	WT
FOLFOX-4	3.11	3.15	3.55	3.05	2.34	1.98	2.63	0	67.88	WT	MUT
FOLFOX-4	2.91	2.40	2.76	1.85	2.18	2.29	-1.30	0	71.33	WT	NA
FOLFOX-4	3.22	2.78	3.17	2.08	2.57	2.72	1.81	1	66.6	WT	WT
FOLFOX-4	3.31	2.73	3.36	2.90	1.64	1.81	0.25	0	59.66	WT	WT
FOLFOX-4	3.11	2.92	3.40	2.38	2.19	2.08	1.08	0	59.66	WT	WT
FOLFOX-4	3.15	2.84	3.31	2.88	2.12	1.94	1.15	0	77.37	WT	NA
FOLFOX-4	2.64	2.74	3.03	2.44	2.02	1.83	-0.93	0	82.17	MUT	NA
FOLFOX-4	3.28	2.74	3.30	2.53	2.13	2.16	1.05	0	66.66	WT	MUT
FOLFOX-4	3.49	2.84	3.39	2.78	1.85	2.12	1.33	0	84.63	WT	MUT
FOLFOX-4	3.28	2.65	3.32	2.52	1.91	2.02	0.36	0	77.96	WT	NA
FOLFOX-4	3.08	3.30	3.52	2.98	2.60	2.67	3.98	0	60.02	WT	MUT
FOLFOX-4	3.10	2.52	3.10	2.24	2.26	2.20	0.12	0	88.38	WT	NA
FOLFOX-4	3.25	2.88	3.51	2.31	2.13	2.51	1.69	0	71.89	WT	MUT
FOLFOX-4	3.07	2.78	2.80	2.68	2.10	2.25	0.31	0	81.25	WT	NA
FOLFOX-4	2.96	2.74	3.19	2.33	1.74	2.03	-0.69	0	78.75	WT	WT
FOLFOX-4	2.99	2.52	3.14	2.51	1.88	2.45	-0.07	0	79.31	WT	NA
FOLFOX-4	2.91	3.04	3.32	2.27	1.81	2.52	0.55	0	80.26	WT	MUT
FOLFOX-4	3.19	2.76	3.04	2.76	2.02	2.27	0.76	0	81.87	WT	NA
FOLFOX-4	3.35	3.14	3.12	3.28	2.62	1.80	2.86	0	47.61	WT	MUT

FOLFOX-4	2.91	3.04	3.32	2.27	1.81	2.52	0.55	0	80.72	WT	MUT
FOLFOX-4	2.97	2.70	3.12	2.57	1.88	2.26	-0.01	1	32.59	WT	MUT
FOLFOX-4	2.96	2.72	3.25	2.61	1.86	1.80	-0.36	0	81.58	WT	MUT
FOLFOX-4	3.31	2.83	3.31	2.65	2.24	2.19	1.60	0	67.84	MUT	WT
FOLFOX-4	2.79	2.63	2.97	2.51	1.94	1.77	-1.12	0	76.22	WT	NA
FOLFOX-4	3.23	2.95	3.53	3.09	1.75	2.75	2.28	0	72.71	WT	WT
FOLFOX-4	3.41	3.09	3.43	3.05	2.32	2.06	2.81	0	79.18	WT	MUT
FOLFOX-4	2.87	2.73	3.36	2.84	1.96	2.09	0.53	0	75.17	WT	NA
FOLFOX-4	3.39	2.51	3.18	2.47	2.14	2.70	1.26	0	86.24	WT	MUT
FOLFOX-4	3.59	2.94	3.27	2.95	2.06	2.09	2.00	0	18.6	WT	WT
FOLFOX-4	2.92	2.53	2.95	2.34	2.20	2.30	-0.20	0	76.62	WT	NA
FOLFOX-4	2.82	2.40	3.10	2.50	2.04	1.85	-0.98	1	45.86	WT	NA
FOLFOX-4	3.02	2.84	3.29	2.94	2.27	1.75	1.08	0	62.16	WT	NA
FOLFOX-4	2.92	2.69	3.59	2.58	2.17	2.03	0.88	1	53.06	WT	WT
FOLFOX-4	3.46	3.07	3.30	3.04	2.11	1.91	2.04	0	77.6	WT	WT
FOLFOX-4	2.99	2.53	2.96	2.27	1.81	1.76	-1.57	0	60.91	WT	MUT
FOLFOX-4	3.32	3.10	3.18	2.81	2.04	2.13	1.60	0	48.16	WT	WT
FOLFOX-4	3.03	2.63	3.06	2.01	2.09	1.93	-0.77	0	75.93	WT	MUT
FOLFOX-4	2.96	2.82	3.21	2.38	2.03	2.42	0.55	0	79.34	WT	MUT
FOLFOX-4	3.17	3.12	3.33	2.87	2.08	2.24	1.91	0	66.53	MUT	WT
FOLFOX-4	3.23	2.85	3.22	2.82	2.11	2.14	1.29	1	40.25	WT	WT
FOLFOX-4	3.00	2.88	3.37	2.80	1.99	2.13	1.00	0	66.33	WT	NA
FOLFOX-4	3.27	3.07	3.09	2.74	2.28	1.99	1.54	0	83.22	WT	MUT
FOLFOX-4	3.04	2.62	3.30	2.69	1.85	2.13	0.14	0	73.33	WT	NA
FOLFOX-4	2.74	2.77	2.72	2.74	1.69	1.83	-1.49	0	85.55	WT	NA
FOLFOX-4	2.87	2.81	3.31	2.68	1.97	2.11	0.42	0	67.38	WT	MUT
FOLFOX-4	2.89	2.73	3.05	2.72	1.93	1.48	-0.84	0	85.06	WT	MUT
FOLFOX-4	2.86	2.49	3.05	1.62	2.10	2.02	-1.55	0	59.86	WT	WT
FOLFOX-4	3.24	3.15	3.42	3.03	2.20	2.51	2.93	0	65.81	WT	MUT
FOLFOX-4	2.94	2.85	2.88	2.58	1.91	2.37	0.02	1	49.68	WT	MUT
FOLFOX-4	2.74	2.72	2.81	1.93	1.98	2.20	-1.39	0	67.48	WT	NA
FOLFOX-4	3.07	2.89	3.35	2.78	1.93	1.78	0.51	0	76.78	WT	NA
FOLFOX-4	2.69	2.57	3.19	2.63	1.83	1.84	-1.02	0	80.39	WT	NA
FOLFOX-4	2.88	2.56	3.16	1.52	1.88	2.12	-1.67	0	83.78	WT	WT
FOLFOX-4	2.83	3.05	2.95	2.88	2.16	2.40	1.13	0	84.14	WT	NA
FOLFOX-4	3.29	2.67	3.30	2.83	2.35	2.06	1.59	0	78.49	MUT	WT
FOLFOX-4	3.10	2.59	3.15	1.95	1.96	2.53	-0.21	0	68.47	WT	MUT
FOLFOX-4	3.28	2.58	3.19	2.48	2.08	2.43	0.81	0	60.16	WT	NA
FOLFOX-4	3.36	2.69	3.40	2.00	2.09	2.57	0.99	0	59.96	WT	MUT
FOLFOX-4	3.10	2.89	3.57	2.79	1.99	2.36	1.73	0	78.16	WT	NA
FOLFOX-4	3.05	2.48	3.25	3.11	2.00	1.61	0.04	0	83.22	WT	NA
FOLFOX-4	3.14	2.88	3.13	2.88	2.21	1.91	1.09	0	61.7	WT	MUT
FOLFOX-4	2.62	2.44	2.94	2.12	1.56	2.21	-2.36	1	52.9	WT	MUT
FOLFOX-4	2.73	2.33	3.05	1.99	1.81	1.85	-2.31	0	83.42	WT	WT
FOLFOX-4	2.60	2.53	2.77	2.40	1.78	1.45	-2.67	0	56.34	WT	NA
FOLFOX-4	2.60	2.07	2.59	1.99	1.72	1.63	-3.98	0	79.84	WT	NA

FOLFOX-4+Bevacizumab	2.91	3.04	3.32	2.27	1.81	2.52	0.55	0	80.39	WT	WT
FOLFOX-4+Bevacizumab	3.10	2.77	3.22	2.64	1.88	2.06	0.24	0	72.02	WT	WT
FOLFOX-4+Bevacizumab	2.69	2.27	2.94	2.05	2.00	2.51	-1.40	0	79.77	MUT	NA
FOLFOX-4+Bevacizumab	3.05	2.52	3.38	2.34	2.10	2.12	0.18	0	68.4	WT	WT
FOLFOX-4+Bevacizumab	3.07	3.15	3.21	2.76	1.90	2.43	1.40	0	81.08	MUT	WT
FOLFOX-4+Bevacizumab	3.08	2.73	3.37	2.63	2.05	2.13	0.77	0	76.75	WT	WT
FOLFOX-4+Bevacizumab	3.15	2.79	3.23	2.89	2.16	2.14	1.30	0	62.32	WT	MUT
FOLFOX-4+Bevacizumab	3.00	2.94	3.05	2.62	1.98	1.97	0.20	0	60.58	WT	MUT
FOLFOX-4+Bevacizumab	2.96	2.93	3.16	2.61	1.62	2.67	0.43	0	84.3	WT	MUT
FOLFOX-4+Bevacizumab	3.09	2.90	3.15	2.83	2.10	2.09	1.02	0	80.99	WT	MUT
FOLFOX-4+Bevacizumab	2.91	2.61	2.94	2.67	2.04	1.76	-0.66	0	81.77	WT	MUT
FOLFOX-4+Bevacizumab	3.40	3.12	3.31	2.90	2.12	2.79	2.98	0	60.32	WT	MUT
FOLFOX-4+Bevacizumab	2.92	3.04	2.97	2.92	1.95	1.65	0.04	0	62.95	WT	NA
FOLFOX-4+Bevacizumab	2.99	2.81	3.20	2.24	1.71	2.21	-0.48	0	87.39	WT	WT
FOLFOX-4+Bevacizumab	3.22	2.60	3.20	2.78	2.17	1.62	0.31	0	43.73	WT	MUT
FOLFOX-4+Bevacizumab	3.38	2.81	3.15	2.65	2.03	2.34	1.22	0	49.05	WT	MUT
FOLFOX-4+Bevacizumab	3.03	2.85	3.39	2.81	1.82	1.90	0.42	0	76.91	WT	WT
FOLFOX-4+Bevacizumab	3.22	2.61	3.29	2.63	2.15	2.15	0.91	0	73.2	WT	MUT
FOLFOX-4+Bevacizumab	3.17	2.96	3.10	2.76	2.37	2.29	1.84	0	67.45	NA	WT
FOLFOX-4+Bevacizumab	3.09	2.69	3.63	2.57	1.84	1.93	0.43	0	84.44	WT	NA
FOLFOX-4+Bevacizumab	3.01	2.37	2.83	2.00	2.11	2.11	-1.30	0	65.15	WT	NA
FOLFOX-4+Bevacizumab	3.29	2.40	3.07	2.50	2.17	2.08	0.14	0	61.63	MUT	NA
FOLFOX-4+Bevacizumab	3.32	2.72	3.20	2.68	2.26	2.62	1.90	0	79.44	WT	MUT
FOLFOX-4+Bevacizumab	2.82	2.66	3.18	2.13	1.91	2.34	-0.54	0	66.79	WT	MUT
FOLFOX-4+Bevacizumab	3.21	2.76	3.31	2.64	2.05	2.10	0.91	0	78.52	WT	MUT
FOLFOX-4+Bevacizumab	3.28	2.72	3.20	2.69	2.05	2.56	1.39	0	74.58	WT	MUT
FOLFOX-4+Bevacizumab	2.96	2.86	3.54	2.73	1.85	2.18	0.87	0	77.93	WT	WT
FOLFOX-4+Bevacizumab	3.28	2.78	3.13	2.87	2.16	1.89	0.98	0	85.03	WT	MUT
FOLFOX-4+Bevacizumab	3.12	2.76	3.07	2.16	1.91	2.13	-0.39	0	79.93	WT	MUT
FOLFOX-4+Bevacizumab	3.01	2.89	3.06	2.83	2.16	2.05	0.82	0	84.24	WT	MUT
FOLFOX-4+Bevacizumab	2.65	2.64	3.06	2.64	1.86	1.75	-1.20	0	80.72	WT	NA
FOLFOX-4+Bevacizumab	3.33	2.90	3.44	2.62	2.25	2.37	2.13	0	67.15	WT	MUT
FOLFOX-4+Bevacizumab	3.13	3.19	3.48	2.94	2.08	1.84	1.79	0	77.63	WT	WT
FOLFOX-4+Bevacizumab	3.28	2.63	3.15	2.23	2.09	1.88	-0.12	0	81.54	WT	MUT
FOLFOX-4+Bevacizumab	2.81	2.66	3.15	2.64	1.80	1.97	-0.66	0	75.89	WT	NA
FOLFOX-4+Bevacizumab	2.99	2.64	3.24	2.85	1.89	2.05	0.20	0	83.94	WT	NA
FOLFOX-4+Bevacizumab	3.24	2.88	3.07	2.79	2.12	2.28	1.31	0	58.41	WT	MUT
FOLFOX-4+Bevacizumab	2.91	3.04	3.32	2.27	1.81	2.52	0.55	0	80.49	WT	NA
FOLFOX-4+Bevacizumab	3.30	2.61	3.09	2.23	2.19	2.67	0.93	0	66.46	WT	MUT
FOLFOX-4+Bevacizumab	3.15	2.56	3.19	2.05	1.93	2.39	-0.22	0	74.02	WT	MUT
FOLFOX-4+Bevacizumab	3.37	2.67	3.63	2.30	2.58	2.65	2.73	0	81.91	WT	WT
FOLFOX-4+Bevacizumab	2.91	3.04	3.32	2.27	1.81	2.52	0.55	0	78.52	WT	MUT
FOLFOX-4+Bevacizumab	3.87	3.09	2.68	2.63	2.68	2.80	3.37	0	80.3	WT	MUT
FOLFOX-4+Bevacizumab	2.92	2.28	3.05	1.98	1.82	2.23	-1.67	0	88.15	WT	MUT
FOLFOX-4+Bevacizumab	2.61	2.41	2.93	1.76	1.88	1.90	-2.60	0	48.62	WT	NA
FOLFOX-4+Bevacizumab	3.13	3.05	3.29	2.50	2.34	2.85	2.50	0	79.93	WT	MUT

FOLFOX-4+Bevacizumab	2.60	2.43	3.08	2.41	1.68	1.81	-2.07	0	77.17	WT	NA
FOLFOX-4+Bevacizumab	3.02	2.99	3.07	2.39	2.20	2.62	1.24	0	61.54	WT	MUT
FOLFOX-4+Bevacizumab	3.27	3.11	3.23	2.80	1.97	2.34	1.72	0	79.9	WT	MUT
FOLFOX-4+Bevacizumab	2.87	2.70	3.19	2.64	2.02	1.79	-0.26	0	48.82	WT	NA
FOLFOX-4+Bevacizumab	3.13	2.96	3.27	2.92	1.99	1.78	0.85	0	78.42	WT	NA
FOLFOX-4+Bevacizumab	3.12	2.93	3.18	2.87	2.17	1.91	1.11	0	82.17	WT	NA
FOLFOX-4+Bevacizumab	3.06	3.06	3.15	2.91	2.02	1.88	0.92	0	66.53	WT	MUT
FOLFOX-4+Bevacizumab	2.82	2.70	3.29	2.73	2.00	2.11	0.28	0	82.2	WT	NA
FOLFOX-4+Bevacizumab	2.90	3.04	2.93	2.58	1.95	2.57	0.63	1	80.62	WT	MUT
FOLFOX-4+Bevacizumab	2.61	2.68	3.25	2.60	1.69	1.66	-1.37	0	64.95	WT	NA
FOLFOX-4+Bevacizumab	2.86	2.77	3.51	2.78	1.91	1.85	0.35	1	68.76	WT	NA
FOLFOX-4+Bevacizumab	2.89	2.84	3.09	2.39	2.01	2.12	-0.10	0	75.73	WT	NA
FOLFOX-4+Bevacizumab	3.23	2.86	3.11	2.24	2.10	2.43	0.80	0	68.99	WT	MUT
FOLFOX-4+Bevacizumab	3.01	2.54	3.18	2.25	1.94	2.43	-0.15	0	68.37	WT	NA
FOLFOX-4+Bevacizumab	2.80	2.62	3.04	2.67	1.99	1.72	-0.81	0	79.93	WT	NA
FOLFOX-4+Bevacizumab	3.19	2.76	3.48	2.38	2.07	2.08	0.85	0	87.13	WT	MUT
FOLFOX-4+Bevacizumab	3.22	2.56	3.17	2.40	2.14	1.94	0.12	0	73.3	WT	NA
FOLFOX-4+Bevacizumab	3.26	2.98	3.38	2.80	1.93	2.67	2.05	0	72.8	WT	MUT
FOLFOX-4+Bevacizumab	3.24	2.34	3.33	2.25	1.92	2.21	-0.23	0	63.87	MUT	WT
FOLFOX-4+Bevacizumab	3.12	2.89	3.46	2.91	1.96	2.09	1.33	0	72.18	WT	WT
FOLFOX-4+Bevacizumab	2.89	2.88	3.03	2.63	1.93	1.89	-0.27	0	54.54	WT	MUT
FOLFOX-4+Bevacizumab	3.03	3.15	3.35	2.89	2.03	2.73	2.33	1	34.56	WT	MUT
FOLFOX-4+Bevacizumab	3.14	2.85	3.62	2.91	1.95	2.06	1.48	0	84.21	WT	WT
FOLFOX-4+Bevacizumab	2.99	2.56	3.22	2.65	2.17	1.93	0.21	0	72.25	WT	NA
FOLFOX-4+Bevacizumab	3.19	3.02	3.27	2.72	2.05	2.18	1.39	1	46.42	WT	MUT
FOLFOX-4+Bevacizumab	3.00	3.08	3.37	3.02	2.33	2.44	2.59	0	67.19	WT	MUT
FOLFOX-4+Bevacizumab	3.06	2.57	3.18	2.43	2.13	2.00	0.00	0	69.22	WT	MUT
FOLFOX-4+Bevacizumab	2.73	2.84	3.21	2.83	1.86	2.26	0.29	0	85.75	WT	NA
FOLFOX-4+Bevacizumab	3.10	2.93	3.55	2.72	2.23	2.65	2.47	0	78.88	WT	MUT
FOLFOX-4+Bevacizumab	3.26	3.07	3.09	2.93	2.32	2.52	2.46	0	86.57	WT	MUT
FOLFOX-4+Bevacizumab	2.76	2.95	3.20	2.53	2.06	1.93	0.10	0	61.14	WT	NA
FOLFOX-4+Bevacizumab	3.17	3.28	3.27	2.92	2.37	2.13	2.55	0	84.99	WT	NA
FOLFOX-4+Bevacizumab	2.91	3.04	3.32	2.27	1.81	2.52	0.55	0	79.74	WT	MUT
FOLFOX-4+Bevacizumab	2.96	2.69	3.03	2.25	2.12	2.47	0.16	0	71.79	WT	WT
FOLFOX-4+Bevacizumab	3.27	2.98	3.32	2.80	2.09	2.38	1.92	0	84.21	WT	MUT
FOLFOX-4+Bevacizumab	2.76	2.58	2.97	2.50	1.84	1.61	-1.63	0	78.52	WT	WT
FOLFOX-4+Bevacizumab	3.32	2.84	3.32	2.42	2.09	2.64	1.65	0	72.15	WT	NA
FOLFOX-4+Bevacizumab	3.03	2.63	3.48	2.65	1.86	1.94	0.17	0	62.32	WT	WT
FOLFOX-4+Bevacizumab	3.01	2.81	3.27	2.40	1.99	2.00	0.12	0	61.54	WT	MUT
FOLFOX-4+Bevacizumab	3.13	2.78	3.01	2.57	2.16	1.76	0.09	0	82.04	WT	MUT
FOLFOX-4+Bevacizumab	2.62	2.53	3.04	2.54	1.97	1.67	-1.43	0	66.89	WT	NA
FOLFOX-4+Bevacizumab	3.32	2.94	2.97	2.41	2.13	2.01	0.57	1	42.64	WT	MUT
FOLFOX-4+Bevacizumab	2.99	2.69	3.01	3.01	1.97	1.92	0.13	0	84.01	WT	WT
FOLFOX-4+Bevacizumab	2.76	2.58	2.79	2.44	1.93	1.87	-1.50	0	72.21	WT	NA
FOLFOX-4+Bevacizumab	3.17	2.96	3.07	2.82	2.08	1.94	0.86	0	74.91	WT	MUT
FOLFOX-4+Bevacizumab	2.91	3.04	3.32	2.27	1.81	2.52	0.55	0	75.33	WT	NA

FOLFOX-4+Bevacizumab	2.69	2.57	2.95	2.31	1.86	1.62	-1.94	0	72.61	WT	MUT
FOLFOX-4+Bevacizumab	3.08	2.65	3.47	2.48	2.22	2.21	1.06	0	66.37	WT	NA
FOLFOX-4+Bevacizumab	2.97	2.73	2.94	2.80	2.02	2.35	0.43	0	62.59	WT	NA
FOLFOX-4+Bevacizumab	3.23	2.88	3.25	2.36	2.21	1.94	0.81	0	60.25	WT	MUT
FOLFOX-4+Bevacizumab	3.25	3.08	3.42	2.86	2.16	2.03	2.00	0	68.17	WT	NA
FOLFOX-4+Bevacizumab	2.82	2.69	3.28	2.50	2.03	1.97	-0.13	0	72.02	WT	MUT
FOLFOX-4+Bevacizumab	3.33	2.77	3.30	2.45	1.86	2.26	0.69	0	74.55	WT	MUT
FOLFOX-4+Bevacizumab	2.77	3.09	3.09	3.00	1.89	1.80	0.23	0	76.75	WT	NA
FOLFOX-4+Bevacizumab	3.17	2.97	3.22	2.71	2.08	1.74	0.75	0	83.25	WT	NA
FOLFOX-4+Bevacizumab	2.89	2.79	3.39	2.57	1.74	1.65	-0.57	0	78.19	WT	NA
FOLFOX-4+Bevacizumab	2.53	2.67	2.85	2.26	1.76	1.85	-2.16	0	78.55	NA	NA
FOLFOX-4+Bevacizumab	2.86	2.38	3.12	2.49	2.02	1.69	-1.17	0	67.38	WT	NA
FOLFOX-4+Bevacizumab	2.85	2.45	3.17	2.57	1.90	2.24	-0.46	0	69.26	WT	NA
FOLFOX-4+Bevacizumab	2.86	2.71	3.36	2.51	1.69	2.05	-0.46	0	81.12	WT	WT
FOLFOX-4+Bevacizumab	3.53	2.88	2.62	2.91	2.21	1.71	0.63	0	68.76	WT	MUT
FOLFOX-4+Bevacizumab	2.82	2.85	3.06	2.63	1.89	1.96	-0.36	0	80	WT	NA
FOLFOX-4+Bevacizumab	3.22	2.77	3.42	2.64	2.15	1.85	0.97	0	79.87	WT	MUT
FOLFOX-4+Bevacizumab	2.64	2.31	2.81	2.38	1.78	1.83	-2.43	0	78.16	WT	MUT
FOLFOX-4+Bevacizumab	2.86	2.95	2.98	2.68	2.03	1.72	-0.21	0	65.97	WT	NA
FOLFOX-4+Bevacizumab	3.06	2.77	3.08	2.29	2.22	2.29	0.52	0	79.74	MUT	NA
FOLFOX-4+Bevacizumab	3.29	2.93	3.15	2.60	2.11	2.67	1.78	1	23.72	WT	WT
FOLFOX-4+Bevacizumab	2.85	2.58	3.10	2.70	2.03	1.85	-0.44	0	77.24	WT	WT
FOLFOX-4+Bevacizumab	3.31	3.28	3.29	2.82	2.08	1.77	1.66	0	65.61	WT	MUT
FOLFOX-4+Bevacizumab	3.16	2.88	3.50	2.92	1.89	1.80	0.95	0	72.05	WT	WT
FOLFOX-4+Bevacizumab	3.26	2.67	3.17	2.47	2.19	2.44	1.12	0	65.45	WT	NA
FOLFOX-4+Bevacizumab	2.99	2.83	2.86	2.25	1.92	2.05	-0.73	0	81.25	MUT	WT
FOLFOX-4+Bevacizumab	3.06	2.69	2.97	2.62	1.80	2.35	-0.10	0	79.84	WT	MUT
FOLFOX-4+Bevacizumab	3.11	2.61	3.16	2.21	1.73	2.28	-0.57	0	84.47	NA	NA
FOLFOX-4+Bevacizumab	2.99	2.70	3.16	2.53	2.14	2.56	0.86	0	59.99	WT	WT
FOLFOX-4+Bevacizumab	2.99	2.56	2.97	1.83	1.95	2.08	-1.37	0	66.69	WT	NA
FOLFOX-4+Bevacizumab	3.35	2.53	2.99	2.42	2.06	2.04	-0.05	0	66.1	WT	MUT
FOLFOX-4+Bevacizumab	3.03	2.96	3.03	2.40	2.20	2.40	0.91	0	62.26	WT	MUT
FOLFOX-4+Bevacizumab	2.75	2.64	3.21	2.50	1.83	1.78	-1.02	0	80.33	WT	WT
FOLFOX-4+Bevacizumab	3.07	2.73	3.19	1.74	2.01	2.10	-0.63	0	83.98	WT	WT
FOLFOX-4+Bevacizumab	2.60	2.52	3.08	2.16	1.51	1.83	-2.57	0	60.42	WT	NA
FOLFOX-4+Bevacizumab	3.16	2.82	3.57	2.54	2.25	2.69	2.31	0	54.08	WT	WT
FOLFOX-4+Bevacizumab	3.06	2.57	3.04	2.39	2.42	2.28	0.64	1	22.11	NA	WT
FOLFOX-4+Bevacizumab	2.96	2.82	2.92	2.52	2.00	2.60	0.43	0	78.69	WT	NA
FOLFOX-4+Bevacizumab	2.78	2.38	2.70	2.45	2.06	1.84	-1.67	0	82.4	WT	NA
FOLFOX-4+Bevacizumab	3.20	3.01	3.19	1.95	1.94	2.47	0.51	0	59.7	WT	MUT
FOLFOX-4+Bevacizumab	2.75	2.57	3.10	2.43	1.77	1.94	-1.28	0	74.78	WT	NA
FOLFOX-4+Bevacizumab	3.68	3.01	3.40	2.51	2.24	2.46	2.69	1	34.23	NA	WT
FOLFOX-4+Bevacizumab	2.83	2.51	2.90	2.26	2.14	1.85	-1.17	0	64.16	WT	NA
FOLFOX-4+Bevacizumab	3.13	2.84	3.57	3.05	2.06	1.96	1.63	1	67.38	WT	MUT
FOLFOX-4+Bevacizumab	3.63	3.20	2.97	3.08	2.23	2.55	3.03	0	81.18	WT	MUT
FOLFOX-4+Bevacizumab	2.97	2.61	3.41	2.37	2.08	1.93	0.04	0	61.57	WT	NA

FOLFOX-4+Bevacizumab	2.71	2.63	2.59	2.57	1.84	1.59	-2.15	0	76.88	WT	NA
FOLFOX-4+Bevacizumab	2.82	2.51	2.54	2.51	1.98	2.26	-1.25	0	80.3	WT	MUT
FOLFOX-4+Bevacizumab	3.08	2.89	3.33	2.24	2.34	2.00	0.91	0	60.94	MUT	WT
FOLFOX-4+Bevacizumab	2.52	2.49	2.85	1.74	1.76	2.14	-2.68	0	70.21	WT	WT
FOLFOX-4+Bevacizumab	2.68	2.64	3.08	2.36	1.75	1.83	-1.58	0	85.26	WT	NA
FOLFOX-4+Bevacizumab	3.00	2.61	2.79	2.34	1.92	2.36	-0.67	0	77.11	WT	MUT
FOLFOX-4+Bevacizumab	3.17	2.69	2.95	2.35	1.90	2.58	0.17	0	65.81	WT	MUT
FOLFOX-4+Bevacizumab	3.19	2.15	3.53	2.57	2.27	2.54	1.13	0	79.21	MUT	NA
FOLFOX-4+Bevacizumab	2.34	2.46	2.82	2.12	1.60	1.87	-3.21	0	60.71	WT	NA
FOLFOX-4+Bevacizumab	2.70	2.41	2.86	2.35	1.74	2.06	-1.97	0	78	WT	MUT
FOLFOX-4+Bevacizumab	3.16	2.49	2.90	2.08	1.93	1.92	-1.30	0	79.77	WT	MUT
FOLFOX-4+Bevacizumab	3.28	3.04	3.44	2.98	2.15	1.80	1.85	0	76.19	WT	MUT
FOLFOX-4+Bevacizumab	2.70	2.68	2.91	2.61	1.92	1.54	-1.44	0	80.03	WT	MUT
FOLFOX-4+Bevacizumab	3.07	3.08	3.20	3.21	2.15	1.82	1.55	0	79.38	WT	WT
FOLFOX-4+Bevacizumab	3.26	2.73	3.10	2.48	2.13	2.39	0.94	0	76.39	WT	MUT
FOLFOX-4+Bevacizumab	2.73	2.46	2.89	2.12	1.77	2.26	-1.78	0	74.38	WT	MUT
FOLFOX-4+Bevacizumab	2.91	2.76	3.28	2.78	2.05	1.56	-0.04	0	83.61	WT	WT
FOLFOX-4+Bevacizumab	3.01	2.92	3.29	2.78	2.11	2.08	1.10	0	46.16	WT	MUT
FOLFOX-4+Bevacizumab	3.03	3.10	3.33	2.53	2.17	2.85	2.20	1	65.18	MUT	WT
FOLFOX-4+Bevacizumab	2.99	2.34	3.26	2.24	2.13	2.46	0.01	0	84.5	WT	MUT
FOLFOX-4+Bevacizumab	3.15	2.25	3.30	2.57	2.04	1.97	-0.23	0	78.39	WT	MUT
FOLFOX-4+Bevacizumab	2.76	2.49	3.33	2.47	1.65	2.14	-0.99	0	79.18	WT	MUT
FOLFOX-4+Bevacizumab	3.41	2.86	3.45	3.13	2.35	1.85	2.39	0	82.4	WT	MUT
FOLFOX-4+Bevacizumab	2.83	2.72	2.82	2.34	1.83	2.16	-1.08	0	77.6	WT	MUT
FOLFOX-4+Bevacizumab	3.32	2.49	2.91	2.22	2.16	2.06	-0.29	0	82.07	WT	MUT
FOLFOX-4+Bevacizumab	3.04	2.62	3.32	2.82	2.14	2.11	0.87	0	62.59	WT	NA
FOLFOX-4+Bevacizumab	2.77	2.66	3.27	2.61	1.95	1.97	-0.29	0	82.17	WT	NA
FOLFOX-4+Bevacizumab	3.20	2.87	3.34	2.16	2.18	2.44	1.19	0	77.11	WT	MUT
FOLFOX-4+Bevacizumab	3.06	2.65	2.99	1.90	2.01	2.17	-0.79	0	66.1	WT	WT
FOLFOX-4+Bevacizumab	3.06	3.03	3.11	2.87	2.05	1.93	0.88	0	67.42	MUT	NA
FOLFOX-4+Bevacizumab	3.19	2.79	3.20	2.65	2.05	1.98	0.62	0	77.86	WT	NA
FOLFOX-4+Bevacizumab	2.88	2.81	3.28	2.33	1.89	2.53	0.34	0	60.55	WT	MUT
FOLFOX-4+Bevacizumab	3.12	2.86	3.31	2.73	1.94	2.03	0.77	0	71.89	WT	MUT
FOLFOX-4+Bevacizumab	3.06	2.88	3.15	2.21	2.01	1.78	-0.32	0	77.47	WT	NA
FOLFOX-4+Bevacizumab	2.85	2.86	3.27	2.43	1.78	2.21	-0.13	0	77.9	WT	MUT
FOLFOX-4+Bevacizumab	2.71	2.24	3.01	1.89	2.19	1.82	-1.95	0	84.44	WT	NA
FOLFOX-4+Bevacizumab	3.34	2.51	3.09	2.28	1.98	2.55	0.37	0	80.33	WT	MUT
FOLFOX-4+Bevacizumab	3.17	2.62	3.07	2.16	2.08	2.41	0.14	0	71.43	WT	MUT
FOLFOX-4+Bevacizumab	3.19	2.93	3.06	2.28	2.28	2.39	1.15	0	85.52	WT	WT
FOLFOX-4+Bevacizumab	3.47	2.92	3.42	2.62	2.14	2.35	2.10	0	61.96	WT	MUT
FOLFOX-4+Bevacizumab	3.11	2.93	3.35	2.63	2.17	2.04	1.24	0	72.54	WT	MUT
FOLFOX-4+Bevacizumab	3.22	2.46	3.60	2.05	2.39	2.22	0.99	0	83.38	MUT	WT
FOLFOX-4+Bevacizumab	2.73	2.68	3.10	2.53	1.86	1.93	-0.87	0	68.34	WT	NA
FOLFOX-4+Bevacizumab	3.13	2.79	2.86	2.67	2.36	2.23	0.96	1	50.3	MUT	WT
FOLFOX-4+Bevacizumab	3.10	2.90	3.93	2.77	2.69	2.06	3.21	0	74.81	WT	NA
FOLFOX-4+Bevacizumab	3.23	2.79	3.33	2.39	2.16	2.65	1.57	1	45.86	WT	MUT

FOLFOX-4+Bevacizumab	3.19	2.51	3.58	2.75	1.69	1.67	-0.18	0	82.37	WT	NA
FOLFOX-4+Bevacizumab	2.94	2.76	3.02	2.64	2.11	1.86	-0.07	0	78.49	MUT	NA
FOLFOX-4+Bevacizumab	2.98	2.84	3.37	2.53	2.03	1.93	0.42	0	60.71	WT	WT
FOLFOX-4+Bevacizumab	2.86	2.64	3.19	2.65	1.95	2.29	0.12	0	84.21	WT	NA
FOLFOX-4+Bevacizumab	3.17	3.12	3.35	2.78	2.05	2.55	2.16	0	72.94	WT	MUT
FOLFOX-4+Bevacizumab	3.38	3.12	2.97	2.66	2.14	2.12	1.39	0	69.49	WT	WT
FOLFOX-4+Bevacizumab	2.94	2.57	3.12	1.89	1.93	2.77	-0.33	0	70.37	WT	MUT
FOLFOX-4+Bevacizumab	3.32	2.58	3.60	2.44	2.21	2.38	1.61	0	64.89	WT	WT
FOLFOX-4+Bevacizumab	3.19	2.58	3.38	2.24	1.98	2.07	0.05	0	82.43	WT	MUT
FOLFOX-4+Bevacizumab	3.00	2.78	3.15	2.40	2.22	2.31	0.71	0	85.82	WT	WT
FOLFOX-4+Bevacizumab	3.03	2.58	3.32	2.71	2.03	2.10	0.43	0	77.8	WT	MUT
FOLFOX-4+Bevacizumab	3.33	2.94	3.20	2.56	1.96	1.87	0.64	1	27.43	WT	MUT
FOLFOX-4+Bevacizumab	3.12	2.58	2.87	2.52	2.10	2.03	-0.29	0	60.98	WT	NA
FOLFOX-4+Bevacizumab	3.07	2.43	2.98	2.19	2.12	2.25	-0.48	0	68.53	WT	WT
FOLFOX-4+Bevacizumab	3.21	2.64	2.97	2.18	2.26	2.32	0.33	0	63.74	WT	NA
FOLFOX-4+Bevacizumab	3.05	2.53	3.35	2.26	2.17	2.09	0.17	0	71.56	WT	MUT
FOLFOX-4+Bevacizumab	3.02	2.81	3.16	2.48	1.93	2.76	0.88	1	76.19	WT	MUT
FOLFOX-4+Bevacizumab	2.95	2.60	3.23	2.46	1.96	2.06	-0.25	0	82.73	WT	NA
FOLFOX-4+Bevacizumab	3.14	2.85	3.36	2.78	2.04	2.18	1.27	0	60.16	WT	MUT
FOLFOX-4+Bevacizumab	3.03	2.98	3.20	2.59	1.68	2.08	0.04	1	80.76	WT	NA
FOLFOX-4+Bevacizumab	3.38	2.29	3.17	2.23	1.99	2.36	-0.07	1	30.09	WT	MUT
FOLFOX-4+Bevacizumab	2.97	2.89	3.03	1.77	2.35	1.76	-0.50	0	67.32	NA	WT
FOLFOX-4+Bevacizumab	3.15	2.56	3.32	2.52	1.90	1.82	-0.23	0	75.07	WT	WT
FOLFOX-4+Bevacizumab	3.48	2.82	3.13	2.77	2.12	2.65	2.04	0	59.66	WT	MUT
FOLFOX-4+Bevacizumab	3.68	2.89	3.28	2.83	2.26	2.42	2.68	0	63.11	WT	MUT
FOLFOX-4+Bevacizumab	2.97	2.51	2.91	2.34	2.06	2.28	-0.50	1	12.45	WT	WT
FOLFOX-4+Bevacizumab	3.44	2.77	3.36	2.63	2.20	2.48	2.04	1	75.47	MUT	WT
FOLFOX-4+Bevacizumab	3.13	2.61	3.16	2.10	2.09	2.29	0.01	0	84.67	WT	NA
FOLFOX-4+Bevacizumab	3.21	3.01	3.45	2.51	2.29	2.46	2.23	0	60.19	MUT	NA
FOLFOX-4+Bevacizumab	3.03	3.05	2.96	2.63	1.91	2.45	0.73	0	60.91	WT	NA
FOLFOX-4+Bevacizumab	3.57	2.83	3.32	2.87	2.28	2.12	2.24	0	29.34	WT	NA
FOLFOX-4+Bevacizumab	3.42	2.96	3.29	2.59	2.18	2.44	2.07	0	66.04	WT	MUT
FOLFOX-4+Bevacizumab	3.04	2.52	3.30	2.60	2.15	2.00	0.32	0	60.25	WT	WT
FOLFOX-4+Bevacizumab	2.96	2.72	3.10	2.57	2.01	2.13	0.07	0	63.57	WT	WT
FOLFOX-4+Bevacizumab	3.26	2.76	3.29	2.45	2.05	2.03	0.63	0	2.79	WT	MUT
FOLFOX-4+Bevacizumab	3.24	2.91	3.19	2.85	2.07	1.64	0.72	1	83.12	WT	MUT
FOLFOX-4+Bevacizumab	3.22	2.58	2.82	2.11	2.30	2.18	-0.11	1	1.15	WT	NA
FOLFOX-4+Bevacizumab	3.27	2.82	3.41	2.78	1.84	1.85	0.67	0	77.54	WT	MUT
FOLFOX-4+Bevacizumab	3.50	2.82	3.27	2.75	2.63	1.99	2.42	1	42.81	MUT	NA
FOLFOX-4+Bevacizumab	2.87	2.11	2.94	2.34	1.90	1.96	-1.88	0	60.52	WT	NA
FOLFOX-4+Bevacizumab	2.46	2.13	2.65	1.88	2.03	1.66	-3.50	0	59.79	MUT	NA
FOLFOX-4+Bevacizumab	3.12	2.71	3.27	2.63	1.91	1.98	0.24	0	63.44	WT	WT
FOLFOX-4+Bevacizumab	3.23	2.95	3.25	2.98	2.45	1.80	1.91	0	59.7	WT	MUT
FOLFOX-4+Bevacizumab	2.99	2.62	3.32	2.80	1.92	2.04	0.28	0	72.54	WT	WT
FOLFOX-4+Bevacizumab	3.20	2.89	3.37	2.76	1.92	2.11	1.10	0	77.54	WT	NA
FOLFOX-4+Bevacizumab	3.25	3.13	3.42	2.91	2.21	2.37	2.62	0	60.94	WT	MUT

FOLFOX-4+Bevacizumab	3.23	2.87	3.26	2.79	1.98	2.09	1.05	0	80.95	WT	WT
FOLFOX-4+Bevacizumab	3.08	3.13	3.36	2.67	1.78	1.90	0.63	1	23.1	WT	MUT
FOLFOX-4+Bevacizumab	3.05	2.68	3.39	1.96	2.06	2.53	0.43	0	65.94	WT	NA
FOLFOX-4+Bevacizumab	2.91	2.90	3.25	2.95	2.11	2.06	1.06	0	60.85	WT	WT
FOLFOX-4+Bevacizumab	2.48	2.51	2.98	2.78	1.73	1.79	-1.77	0	66.07	WT	WT
FOLFOX-4+Bevacizumab	3.19	2.97	3.24	2.40	2.14	2.38	1.31	0	66.53	WT	MUT
FOLFOX-4+Bevacizumab	3.00	2.61	2.99	2.35	2.06	2.48	0.07	1	51.02	WT	MUT
FOLFOX-4+Bevacizumab	2.68	2.63	2.83	2.85	2.01	1.85	-0.87	0	82.63	WT	NA
FOLFOX-4+Bevacizumab	2.72	2.94	2.76	2.40	1.69	2.09	-1.29	0	87.92	WT	NA
FOLFOX-4+Bevacizumab	3.33	2.92	3.55	3.07	2.11	2.26	2.47	0	79.18	WT	WT
FOLFOX-4+Bevacizumab	2.92	2.62	3.24	2.58	2.03	1.95	-0.10	0	83.91	WT	WT
FOLFOX-4+Bevacizumab	2.82	2.57	2.88	2.26	2.18	2.37	-0.42	0	59.2	WT	MUT
FOLFOX-4+Bevacizumab	3.14	2.75	3.01	2.56	2.09	2.24	0.51	0	68.3	WT	WT
FOLFOX-4+Bevacizumab	3.06	2.65	3.31	2.39	2.05	2.79	1.07	0	78.85	WT	MUT
FOLFOX-4+Bevacizumab	3.19	2.79	3.39	2.78	1.97	2.05	0.99	0	77.9	WT	NA
FOLFOX-4+Bevacizumab	3.51	3.07	3.20	2.93	2.38	2.38	2.93	1	41.53	WT	MUT
FOLFOX-4+Bevacizumab	3.06	2.82	3.31	2.68	2.08	2.15	0.95	0	64.62	WT	WT
FOLFOX-4+Bevacizumab	2.94	2.33	3.33	1.83	2.22	2.56	-0.14	1	10.45	WT	NA
FOLFOX-4+Bevacizumab	3.21	2.93	3.12	2.43	2.18	2.57	1.44	1	19.25	MUT	WT
FOLFOX-4+Bevacizumab	2.88	2.77	3.09	2.17	2.20	2.60	0.48	0	78.13	WT	MUT
FOLFOX-4+Bevacizumab	3.21	2.72	3.35	2.36	2.22	2.74	1.67	0	83.15	MUT	NA
FOLFOX-4+Bevacizumab	2.91	2.44	3.17	2.78	2.16	2.00	0.08	0	72.11	WT	WT
FOLFOX-4+Bevacizumab	3.17	2.73	3.04	2.36	1.98	2.03	-0.16	0	78.19	WT	WT
FOLFOX-4+Bevacizumab	3.21	2.50	2.99	2.32	2.08	2.16	-0.23	0	72.34	WT	WT
FOLFOX-4+Bevacizumab	3.00	3.15	3.40	2.64	2.06	2.92	2.35	0	2.79	WT	MUT
FOLFOX-4+Bevacizumab	3.33	2.75	3.16	2.67	2.18	2.29	1.33	0	58.58	WT	WT
FOLFOX-4+Bevacizumab	3.12	2.89	3.24	2.49	2.24	2.51	1.57	0	60.39	MUT	NA
FOLFOX-4+Bevacizumab	2.72	2.47	3.07	2.24	2.16	2.31	-0.57	0	42.71	WT	WT
FOLFOX-4+Bevacizumab	2.72	2.79	3.18	2.72	1.85	1.68	-0.72	1	68.73	WT	NA
FOLFOX-4+Bevacizumab	2.84	2.69	3.19	2.66	1.81	1.84	-0.64	0	60.06	WT	NA
FOLFOX-4+Bevacizumab	2.95	2.64	3.17	2.66	2.02	1.89	-0.12	0	60.06	WT	NA
FOLFOX-4+Bevacizumab	3.05	2.65	3.12	2.47	2.06	2.63	0.69	0	61.01	WT	MUT
FOLFOX-4+Bevacizumab	3.24	2.74	3.24	2.45	2.06	2.35	0.90	0	60.62	WT	NA
FOLFOX-4+Bevacizumab	3.23	3.23	3.06	3.09	2.09	1.89	1.62	0	60.98	WT	MUT
FOLFOX-4+Bevacizumab	3.26	2.63	3.16	2.43	2.23	2.01	0.54	1	33.97	WT	MUT
FOLFOX-4+Bevacizumab	2.94	2.76	3.41	2.46	1.84	2.14	0.12	0	60.94	WT	MUT
FOLFOX-4+Bevacizumab	3.22	2.82	3.31	2.62	2.01	2.42	1.29	0	74.02	WT	MUT
FOLFOX-4+Bevacizumab	3.29	2.66	3.37	2.72	2.26	2.13	1.49	0	63.7	WT	MUT
FOLFOX-4+Bevacizumab	3.22	2.42	3.22	2.50	1.91	1.75	-0.60	0	77.31	WT	NA
FOLFOX-4+Bevacizumab	3.03	2.85	3.15	2.58	1.81	2.06	-0.01	1	34.23	WT	MUT
FOLFOX-4+Bevacizumab	3.22	3.00	3.29	2.80	2.16	1.74	1.22	0	74.78	WT	MUT
FOLFOX-4+Bevacizumab	3.06	2.74	3.20	2.87	1.94	1.96	0.39	0	61.96	WT	WT
FOLFOX-4+Bevacizumab	3.18	2.92	3.64	2.82	2.00	2.11	1.70	0	60.52	WT	WT
FOLFOX-4+Bevacizumab	3.00	2.90	3.38	3.08	2.14	1.83	1.29	0	59.6	WT	NA
FOLFOX-4+Bevacizumab	2.93	2.76	3.42	2.52	1.94	2.14	0.40	0	77.67	WT	WT
FOLFOX-4+Bevacizumab	2.99	2.87	3.09	2.85	2.08	1.89	0.50	0	84.67	WT	NA

FOLFOX-4+Bevacizumab	3.05	2.75	3.26	1.96	2.02	1.86	-0.54	0	78.88	WT	WT
FOLFOX-4+Bevacizumab	3.36	2.90	3.35	2.39	1.91	2.33	1.08	0	79.93	WT	MUT
FOLFOX-4+Bevacizumab	3.48	2.58	3.51	2.14	2.26	1.88	0.83	0	72.9	WT	MUT
FOLFOX-4+Bevacizumab	3.23	3.10	3.31	2.87	2.36	1.95	2.12	0	59.63	NA	WT
XELOX+Bevacizumab	2.91	3.04	3.32	2.27	1.81	2.52	0.55	0	79.77	MUT	WT
XELOX+Bevacizumab	2.91	3.04	3.32	2.27	1.81	2.52	0.55	0	80.89	WT	NA
XELOX+Bevacizumab	3.21	2.90	3.17	2.67	1.76	1.92	0.16	1	43.43	WT	NA
XELOX+Bevacizumab	3.36	2.93	3.65	2.41	2.49	2.51	2.93	1	32.26	WT	MUT
XELOX+Bevacizumab	2.74	2.63	3.24	2.75	2.16	2.29	0.53	0	62.39	WT	WT
XELOX+Bevacizumab	3.04	2.80	2.92	2.82	2.17	2.30	0.85	0	66.2	WT	MUT
XELOX+Bevacizumab	2.92	2.49	3.08	2.31	1.96	2.09	-0.80	1	63.41	WT	MUT
XELOX+Bevacizumab	2.92	2.67	3.05	2.04	1.99	2.46	-0.40	0	85.62	WT	NA
XELOX+Bevacizumab	3.04	2.59	3.22	2.80	1.62	1.86	-0.63	1	24.57	WT	NA
XELOX+Bevacizumab	2.82	2.44	2.91	1.84	1.76	1.79	-2.58	0	74.81	WT	NA
XELOX+Bevacizumab	3.12	2.83	3.35	3.00	2.29	2.20	1.95	0	77.54	WT	MUT
XELOX+Bevacizumab	2.94	2.73	3.45	2.69	1.83	1.88	0.06	0	77.67	WT	WT
XELOX+Bevacizumab	3.13	2.87	3.26	2.56	2.00	2.67	1.37	0	66.86	WT	MUT
XELOX+Bevacizumab	2.81	2.60	3.32	2.67	2.01	2.03	0.00	0	67.09	WT	WT
XELOX+Bevacizumab	2.89	2.40	2.86	2.63	2.03	1.84	-1.11	0	62.88	MUT	NA
XELOX+Bevacizumab	3.22	2.84	3.22	2.81	2.10	1.64	0.63	1	74.35	WT	WT
XELOX+Bevacizumab	3.38	2.66	3.14	2.36	2.18	2.29	0.87	0	73.26	WT	WT
XELOX+Bevacizumab	3.37	3.26	2.96	2.72	2.21	2.16	1.83	1	79.18	WT	NA
XELOX+Bevacizumab	3.06	2.72	3.30	2.58	2.15	1.81	0.39	0	86.51	WT	MUT
XELOX+Bevacizumab	2.98	2.45	3.24	2.11	2.09	1.95	-0.70	0	80.76	WT	NA
XELOX+Bevacizumab	2.99	2.61	3.27	2.48	1.95	2.27	0.15	0	72.84	WT	NA
XELOX+Bevacizumab	3.24	2.87	3.11	2.83	2.21	1.86	1.05	0	64.69	WT	MUT
XELOX+Bevacizumab	3.01	2.57	3.27	2.03	2.01	2.19	-0.41	0	59.79	WT	WT
XELOX+Bevacizumab	3.05	3.03	3.03	2.68	2.05	2.76	1.52	0	64.69	WT	MUT
XELOX+Bevacizumab	3.32	2.80	3.08	2.53	1.83	2.46	0.66	0	82.99	WT	NA
XELOX+Bevacizumab	3.26	2.68	3.25	2.45	2.03	2.03	0.41	0	79.24	WT	NA
XELOX+Bevacizumab	3.21	2.99	3.00	2.74	1.95	2.52	1.22	0	77.77	WT	MUT
XELOX+Bevacizumab	3.41	2.68	3.52	2.60	2.37	2.28	2.15	0	60.06	MUT	WT
XELOX+Bevacizumab	2.86	2.58	3.03	2.23	2.12	1.98	-0.76	0	79.34	WT	WT
XELOX+Bevacizumab	2.91	3.04	3.32	2.27	1.81	2.52	0.55	0	80.59	WT	MUT
XELOX+Bevacizumab	3.06	2.68	3.54	2.63	2.06	2.78	1.73	0	60.22	WT	MUT
XELOX+Bevacizumab	3.34	2.75	3.33	2.57	2.08	2.34	1.37	0	79.21	WT	MUT
XELOX+Bevacizumab	2.93	2.70	3.26	2.30	2.14	2.19	0.23	0	78	WT	WT
XELOX+Bevacizumab	3.07	2.63	3.13	2.41	2.01	2.37	0.23	0	63.21	WT	NA
XELOX+Bevacizumab	3.07	2.90	3.28	2.62	2.37	2.15	1.53	0	78.75	WT	MUT
XELOX+Bevacizumab	2.99	2.75	3.35	2.93	1.90	1.79	0.34	0	71.98	WT	NA
XELOX+Bevacizumab	2.79	2.76	3.28	2.57	2.04	2.23	0.35	0	79.31	WT	MUT
XELOX+Bevacizumab	2.91	3.04	3.32	2.27	1.81	2.52	0.55	0	77.5	WT	NA
XELOX+Bevacizumab	3.00	2.66	3.10	2.30	1.75	2.67	-0.14	1	18.76	WT	MUT
XELOX+Bevacizumab	3.50	2.49	3.47	2.86	2.74	2.24	2.87	0	60.98	WT	WT
XELOX+Bevacizumab	3.16	2.70	3.00	2.71	2.29	2.09	0.80	0	67.75	NA	WT
XELOX+Bevacizumab	3.17	2.84	3.32	2.45	1.76	2.26	0.41	0	65.68	WT	WT

XELOX+Bevacizumab	2.82	2.53	3.03	2.25	1.86	2.54	-0.68	0	78.85	WT	MUT
XELOX+Bevacizumab	2.93	2.77	3.19	2.80	1.98	1.93	0.20	0	81.87	WT	WT
XELOX+Bevacizumab	2.98	2.82	3.11	2.40	1.97	2.14	-0.01	1	44.45	WT	WT
XELOX+Bevacizumab	3.26	3.00	3.57	2.70	2.58	3.26	4.21	0	75.24	MUT	WT
XELOX+Bevacizumab	2.87	2.59	3.27	2.67	1.70	2.09	-0.52	0	73.49	WT	NA
XELOX+Bevacizumab	3.55	3.25	3.22	2.92	2.40	2.69	3.69	0	60.22	MUT	NA
XELOX+Bevacizumab	2.73	2.62	2.81	2.56	1.65	1.94	-1.75	0	78.06	WT	NA
XELOX+Bevacizumab	2.89	2.75	3.15	2.43	1.79	2.16	-0.46	0	67.94	WT	MUT
XELOX+Bevacizumab	3.40	3.27	3.10	2.98	1.99	1.79	1.53	1	52.99	WT	MUT
XELOX+Bevacizumab	3.05	2.82	3.30	2.88	1.89	1.70	0.26	0	80.99	WT	NA
XELOX+Bevacizumab	3.69	2.94	3.13	2.81	2.15	2.69	2.67	0	81.45	WT	MUT
XELOX+Bevacizumab	3.09	3.06	3.40	2.90	1.97	2.01	1.37	0	48.59	WT	MUT
XELOX+Bevacizumab	3.06	3.04	3.28	3.02	2.02	2.03	1.37	0	60.02	WT	NA
XELOX+Bevacizumab	2.46	2.32	3.10	2.26	1.59	1.63	-2.97	0	81.48	WT	WT
XELOX+Bevacizumab	3.01	2.63	3.30	2.35	2.39	1.74	0.28	0	64	MUT	NA
XELOX+Bevacizumab	2.75	2.51	2.96	2.63	1.86	1.56	-1.66	0	81.48	WT	NA
XELOX+Bevacizumab	3.22	2.95	3.04	3.00	2.14	2.56	1.96	0	72.44	WT	MUT
XELOX+Bevacizumab	2.93	2.72	3.52	2.80	1.97	1.95	0.63	0	61.73	WT	NA
XELOX+Bevacizumab	3.59	2.86	3.31	2.65	2.04	1.88	1.28	0	83.38	WT	MUT
XELOX+Bevacizumab	2.88	3.05	2.92	2.63	2.11	2.65	1.07	1	72.67	WT	NA
XELOX+Bevacizumab	2.96	2.77	3.07	2.59	2.09	1.90	0.01	0	60.29	WT	WT
XELOX+Bevacizumab	3.32	2.78	3.31	2.56	2.23	2.46	1.75	0	61.17	MUT	NA
XELOX+Bevacizumab	3.32	2.95	2.91	2.58	2.24	2.39	1.38	0	75.01	NA	NA
XELOX+Bevacizumab	2.81	2.42	3.41	2.54	2.30	2.19	0.46	0	77.21	WT	WT
XELOX+Bevacizumab	3.22	2.84	3.40	3.05	2.08	1.78	1.31	0	82.63	WT	WT
XELOX+Bevacizumab	2.82	3.00	2.94	2.76	1.91	2.54	0.59	0	85.59	WT	MUT
XELOX+Bevacizumab	3.14	2.79	3.27	2.86	2.00	1.80	0.61	0	59.37	WT	WT
XELOX+Bevacizumab	2.88	2.92	3.19	2.62	2.08	2.21	0.67	0	68.5	WT	MUT
XELOX+Bevacizumab	3.10	2.61	3.17	2.69	1.82	1.69	-0.56	0	83.19	WT	NA
XELOX+Bevacizumab	3.13	2.86	3.31	2.94	1.89	2.33	1.27	0	84.93	WT	MUT
XELOX+Bevacizumab	2.73	2.67	2.95	2.70	2.03	1.85	-0.70	0	76.48	WT	NA
XELOX+Bevacizumab	3.32	2.71	3.34	2.68	2.50	2.92	2.91	0	60.09	MUT	WT
XELOX+Bevacizumab	3.37	2.80	3.33	2.62	2.17	2.65	2.07	0	82.83	WT	MUT
XELOX+Bevacizumab	3.11	2.79	3.27	2.59	2.14	2.06	0.82	0	73.2	WT	WT
XELOX+Bevacizumab	2.72	2.58	3.23	1.63	1.73	2.95	-0.91	0	75.07	WT	NA
XELOX+Bevacizumab	2.97	2.64	2.99	2.68	2.26	2.21	0.52	0	76.42	MUT	NA
XELOX+Bevacizumab	3.10	2.72	3.14	2.57	2.03	2.29	0.55	0	76.42	WT	NA
XELOX+Bevacizumab	3.07	2.82	3.25	2.73	1.72	1.79	-0.17	0	65.74	WT	NA
XELOX+Bevacizumab	2.95	2.67	2.77	1.91	1.94	2.56	-0.90	0	71.89	WT	NA
XELOX+Bevacizumab	2.93	2.63	2.97	2.31	1.57	2.34	-1.19	0	77.27	WT	MUT
XELOX+Bevacizumab	2.72	2.73	3.10	2.32	1.81	1.93	-1.16	1	50.04	WT	NA
XELOX+Bevacizumab	3.05	2.71	3.40	2.53	2.26	2.64	1.66	0	61.93	MUT	NA
XELOX+Bevacizumab	3.03	2.72	3.21	2.48	2.06	2.43	0.69	0	75.1	WT	MUT
XELOX+Bevacizumab	3.21	2.70	2.94	2.58	2.13	1.95	0.16	0	79.41	WT	MUT
XELOX+Bevacizumab	3.55	3.00	3.29	2.47	2.47	1.52	1.58	0	78.26	MUT	WT
XELOX+Bevacizumab	3.18	2.63	3.01	2.24	2.12	2.31	0.14	0	80.33	WT	NA

XELOX+Bevacizumab	3.11	2.90	3.06	2.89	2.24	1.83	0.92	0	66.14	WT	NA
XELOX+Bevacizumab	3.02	2.81	3.45	2.62	1.82	1.90	0.23	0	77.96	WT	MUT
XELOX+Bevacizumab	2.79	2.45	3.18	2.41	1.94	2.11	-0.79	0	79.74	WT	MUT
XELOX+Bevacizumab	2.75	2.56	3.08	2.49	2.00	1.92	-0.86	0	69.91	WT	NA
XELOX+Bevacizumab	2.66	3.11	3.47	2.71	1.63	1.56	-0.45	0	83.81	WT	NA
XELOX+Bevacizumab	2.71	2.54	3.30	2.44	1.91	1.63	-1.17	0	77.93	WT	NA
XELOX+Bevacizumab	3.24	2.87	3.28	2.82	1.95	1.95	0.90	0	69.03	WT	MUT
XELOX+Bevacizumab	3.10	2.88	3.50	2.53	2.23	2.47	1.89	0	78.16	WT	NA
XELOX+Bevacizumab	3.35	2.47	2.99	2.78	2.19	2.11	0.61	1	29.11	WT	MUT
XELOX+Bevacizumab	2.67	2.42	3.07	2.09	1.91	2.42	-1.22	0	61.9	WT	NA
XELOX+Bevacizumab	3.08	2.93	3.06	2.50	1.81	2.14	0.05	0	78.85	WT	MUT
XELOX+Bevacizumab	3.05	2.70	2.86	2.42	2.01	1.83	-0.72	0	65.12	WT	MUT
XELOX+Bevacizumab	3.05	3.14	3.28	2.75	2.12	2.46	1.91	0	61.21	MUT	NA
XELOX+Bevacizumab	3.35	3.07	3.10	2.80	2.10	2.19	1.63	0	66.17	WT	WT
XELOX+Bevacizumab	3.28	3.21	3.51	3.11	2.00	2.30	2.68	0	72.02	WT	NA
XELOX+Bevacizumab	3.09	2.55	3.29	2.26	2.21	1.96	0.07	0	53.72	WT	MUT
XELOX+Bevacizumab	3.06	2.60	3.44	2.97	2.01	1.80	0.60	0	76.22	WT	WT
XELOX+Bevacizumab	2.91	3.06	3.08	2.81	1.76	2.73	1.03	0	83.94	WT	WT
XELOX+Bevacizumab	2.90	2.66	3.37	2.29	1.68	2.68	0.01	0	69.75	WT	WT
XELOX+Bevacizumab	2.88	2.98	3.09	2.85	1.82	1.83	-0.04	0	77.31	WT	WT
XELOX+Bevacizumab	3.49	2.82	3.35	2.93	2.04	2.01	1.64	0	72.67	WT	MUT
XELOX+Bevacizumab	2.99	2.65	3.16	2.69	2.04	2.02	0.15	0	64.53	WT	WT
XELOX+Bevacizumab	3.06	2.54	2.97	2.11	1.76	1.82	-1.66	0	61.14	WT	NA
XELOX+Bevacizumab	2.80	2.64	2.86	2.65	1.76	1.65	-1.59	0	80.89	WT	NA
XELOX+Bevacizumab	3.18	2.49	3.35	1.88	2.12	2.37	0.08	0	75.4	WT	NA
XELOX+Bevacizumab	3.16	2.74	3.01	2.32	2.20	2.03	0.20	0	59.93	WT	MUT
XELOX+Bevacizumab	2.57	2.67	2.87	2.22	2.27	2.37	-0.50	0	77.9	WT	NA
XELOX+Bevacizumab	3.09	2.94	3.08	3.17	1.81	1.75	0.39	0	61.17	WT	MUT
XELOX+Bevacizumab	2.40	2.44	2.68	1.80	1.54	2.38	-3.23	0	60.25	WT	WT
XELOX+Bevacizumab	2.91	3.04	3.32	2.27	1.81	2.52	0.55	0	73	WT	MUT
XELOX+Bevacizumab	2.82	2.31	2.96	2.27	1.96	1.93	-1.64	0	83.55	WT	NA
XELOX+Bevacizumab	3.04	2.76	3.08	2.13	1.81	2.43	-0.31	0	79.64	WT	MUT
XELOX+Bevacizumab	3.19	3.29	3.50	2.91	2.26	2.50	3.18	0	76.65	WT	MUT
XELOX+Bevacizumab	3.19	2.67	4.06	2.86	1.91	1.92	1.65	0	84.44	WT	WT
XELOX+Bevacizumab	2.34	2.34	2.75	2.01	1.93	1.81	-3.07	0	68.14	WT	NA
XELOX+Bevacizumab	3.14	3.01	3.39	3.12	1.90	1.75	1.17	0	73.63	WT	MUT
XELOX+Bevacizumab	3.10	2.41	3.07	2.62	2.12	2.52	0.48	0	64.66	WT	MUT
XELOX+Bevacizumab	3.17	2.93	3.43	2.45	1.61	2.04	0.16	0	85.78	WT	MUT
XELOX+Bevacizumab	3.39	2.75	3.28	2.56	1.97	2.53	1.37	0	60.55	WT	MUT
XELOX+Bevacizumab	3.17	2.87	3.19	2.96	1.95	1.53	0.34	0	59.99	WT	WT
XELOX+Bevacizumab	3.00	2.38	3.25	2.41	1.94	2.05	-0.59	0	80.16	WT	WT
XELOX+Bevacizumab	3.19	3.00	3.40	2.97	1.89	1.91	1.21	0	73.03	WT	MUT
XELOX+Bevacizumab	2.86	2.89	3.65	2.95	2.16	1.81	1.37	0	77.6	WT	MUT
XELOX+Bevacizumab	2.78	2.51	2.93	2.27	1.85	1.54	-2.12	0	73.4	WT	MUT
XELOX+Bevacizumab	3.12	2.73	3.17	2.60	2.00	2.31	0.66	0	78.29	WT	MUT
XELOX+Bevacizumab	2.84	2.68	3.01	2.48	2.04	2.45	0.05	0	82.56	WT	WT

XELOX+Bevacizumab	3.16	2.70	3.29	2.39	1.92	2.24	0.34	0	60.25	WT	WT
XELOX+Bevacizumab	2.36	2.38	2.80	2.23	1.77	1.51	-3.31	0	83.42	WT	NA
XELOX+Bevacizumab	2.91	3.04	3.32	2.27	1.81	2.52	0.55	0	77.7	WT	NA
XELOX+Bevacizumab	2.84	3.01	3.56	2.50	2.18	2.41	1.62	0	79.08	MUT	NA
XELOX+Bevacizumab	2.88	2.54	2.87	2.08	2.08	2.49	-0.66	0	83.42	MUT	NA
XELOX+Bevacizumab	2.85	2.68	3.11	2.63	1.81	1.81	-0.81	0	83.19	NA	NA
XELOX+Bevacizumab	2.93	2.60	3.02	2.49	1.88	1.85	-0.97	0	84.4	WT	WT
XELOX+Bevacizumab	3.17	3.03	3.20	2.91	1.91	1.98	0.99	1	2.2	WT	MUT
XELOX+Bevacizumab	3.23	2.80	3.12	2.45	2.15	2.18	0.77	0	60.32	WT	MUT
XELOX+Bevacizumab	2.59	2.30	2.85	2.48	1.49	1.40	-3.40	0	59.86	WT	NA
XELOX+Bevacizumab	3.41	2.63	3.19	2.24	2.20	2.61	1.21	0	57.03	WT	NA
XELOX+Bevacizumab	2.92	2.20	2.66	2.30	1.83	2.05	-2.20	0	61.31	WT	MUT
XELOX+Bevacizumab	3.08	2.73	3.23	2.66	2.03	2.16	0.62	0	60.48	WT	MUT
XELOX+Bevacizumab	2.67	2.89	3.10	2.34	1.94	2.80	0.35	0	80.23	WT	MUT
XELOX+Bevacizumab	2.99	2.32	3.65	2.58	1.97	2.13	0.25	0	76.75	WT	NA
XELOX+Bevacizumab	3.22	2.95	3.31	2.88	2.24	2.97	2.89	0	61.4	WT	WT
XELOX+Bevacizumab	3.10	2.88	3.22	2.73	2.25	2.71	2.01	1	36.7	WT	NA
XELOX+Bevacizumab	2.84	2.42	2.87	1.88	1.85	1.90	-2.31	0	84.17	WT	WT
XELOX+Bevacizumab	3.09	2.95	3.53	2.95	1.98	1.80	1.22	1	20.6	WT	WT
XELOX+Bevacizumab	2.94	2.61	3.36	2.72	1.92	1.83	-0.09	0	61.83	WT	NA
XELOX+Bevacizumab	3.57	2.65	3.36	2.53	2.23	2.56	2.06	1	22.31	MUT	WT
XELOX+Bevacizumab	2.85	2.54	3.39	3.01	1.98	1.91	0.29	0	84.86	WT	NA
XELOX+Bevacizumab	3.49	3.00	3.41	3.03	2.12	2.04	2.31	0	82.04	WT	MUT
XELOX+Bevacizumab	2.62	2.47	2.87	2.38	1.67	1.98	-2.17	0	84.8	WT	NA
XELOX+Bevacizumab	3.14	2.72	3.13	2.76	2.20	2.00	0.78	0	86.31	MUT	WT
XELOX+Bevacizumab	2.97	2.91	3.04	2.92	1.71	1.90	-0.17	0	79.9	WT	NA
XELOX+Bevacizumab	3.29	2.76	3.16	2.49	2.30	2.44	1.49	0	81.97	WT	MUT
XELOX+Bevacizumab	2.90	2.62	3.20	2.37	1.95	1.90	-0.65	0	76.62	WT	NA
XELOX+Bevacizumab	2.70	2.58	3.14	2.63	1.71	2.18	-0.89	0	79.08	WT	NA
XELOX+Bevacizumab	3.01	2.58	3.34	2.14	2.14	2.26	0.18	1	43.01	WT	MUT
XELOX+Bevacizumab	2.64	2.62	2.77	2.32	1.88	1.95	-1.76	0	59.7	WT	NA
XELOX+Bevacizumab	3.02	2.65	3.17	2.56	2.29	1.78	0.25	0	61.44	WT	NA
XELOX+Bevacizumab	2.89	2.70	3.27	2.55	1.83	2.26	-0.02	0	80.03	WT	WT
XELOX+Bevacizumab	3.02	2.91	3.22	2.74	2.12	2.05	0.94	0	84.24	WT	MUT
XELOX+Bevacizumab	3.19	2.68	3.41	2.59	2.08	1.96	0.75	0	79.24	WT	NA
XELOX+Bevacizumab	2.84	2.80	3.31	2.62	2.34	2.37	1.32	0	59.83	MUT	NA
XELOX+Bevacizumab	2.93	2.70	3.17	2.59	1.79	1.66	-0.86	0	79.9	WT	MUT
XELOX+Bevacizumab	3.02	3.09	3.07	2.41	2.24	2.38	1.23	0	76.12	WT	MUT
XELOX+Bevacizumab	2.94	2.56	3.24	2.30	1.71	2.11	-0.90	0	82.99	WT	MUT
XELOX+Bevacizumab	2.73	2.48	2.77	2.21	1.69	1.66	-2.70	0	80.13	WT	NA
XELOX+Bevacizumab	2.81	2.63	3.11	2.65	2.14	1.88	-0.22	0	81.61	WT	MUT
XELOX+Bevacizumab	2.91	3.07	3.35	3.14	2.13	1.83	1.44	0	81.35	WT	MUT
XELOX+Bevacizumab	3.08	2.72	3.24	2.53	1.98	2.16	0.38	0	84.6	WT	WT
XELOX+Bevacizumab	2.72	2.79	2.99	2.43	2.02	2.38	-0.15	0	81.41	MUT	NA
XELOX+Bevacizumab	2.77	2.93	2.99	2.94	2.02	1.30	-0.58	0	69.13	WT	MUT
XELOX+Bevacizumab	2.95	2.94	3.05	2.89	1.92	1.53	-0.21	0	84.21	WT	MUT

XELOX+Bevacizumab	2.94	2.34	2.95	2.25	1.76	1.59	-2.28	0	68.01	WT	MUT
XELOX+Bevacizumab	2.35	2.43	2.93	2.74	1.45	1.43	-3.15	0	83.65	WT	NA
XELOX+Bevacizumab	2.85	2.77	3.11	2.75	1.86	1.81	-0.47	0	75.53	WT	MUT
XELOX+Bevacizumab	3.24	2.94	3.19	2.90	2.39	2.70	2.71	0	65.12	MUT	WT
XELOX+Bevacizumab	2.83	2.54	3.00	2.26	1.90	1.85	-1.44	0	72.25	WT	WT
XELOX+Bevacizumab	3.44	2.49	3.15	2.70	2.16	2.09	0.84	0	60.71	MUT	WT
XELOX+Bevacizumab	2.88	2.80	3.25	2.68	1.96	1.99	0.17	0	86.01	WT	NA
XELOX+Bevacizumab	3.35	2.93	3.35	2.11	2.27	2.27	1.41	0	84.14	WT	WT
XELOX+Bevacizumab	3.36	2.79	3.50	2.87	1.97	2.90	2.50	0	83.78	WT	MUT
XELOX+Bevacizumab	3.14	2.95	3.27	2.83	2.03	2.75	2.01	0	68.8	WT	MUT
XELOX+Bevacizumab	3.03	2.65	3.58	2.73	1.96	1.86	0.53	0	68.24	WT	WT
XELOX+Bevacizumab	2.96	2.61	3.08	2.92	1.89	1.85	-0.28	0	80.07	WT	NA
XELOX+Bevacizumab	3.29	2.89	3.50	2.73	2.31	2.25	2.28	0	77.34	WT	MUT
XELOX+Bevacizumab	3.14	2.71	3.13	2.72	1.91	1.79	-0.08	1	26.64	WT	MUT
XELOX+Bevacizumab	3.31	2.66	3.43	2.38	2.23	2.08	1.07	0	75.76	WT	MUT
XELOX+Bevacizumab	3.11	2.39	2.76	1.88	2.09	2.10	-1.41	0	77.11	WT	MUT
XELOX+Bevacizumab	3.04	2.77	3.10	2.60	2.12	2.07	0.44	0	78.42	WT	NA
XELOX+Bevacizumab	2.91	3.04	3.32	2.27	1.81	2.52	0.55	0	72.28	WT	MUT
XELOX+Bevacizumab	3.23	3.11	3.01	2.92	2.38	1.34	1.07	0	78.62	WT	NA
XELOX+Bevacizumab	3.18	2.63	3.33	2.48	2.00	2.06	0.36	0	84.96	WT	WT
XELOX+Bevacizumab	2.87	2.60	3.23	2.46	2.02	1.87	-0.47	0	60.65	WT	WT
XELOX+Bevacizumab	3.06	2.79	3.05	2.19	2.05	2.33	0.11	0	59.99	WT	WT
XELOX+Bevacizumab	3.03	2.42	3.37	2.48	2.13	2.12	0.22	0	76.58	WT	WT
XELOX+Bevacizumab	2.93	2.50	2.95	1.81	1.88	2.04	-1.78	0	54.24	NA	NA
XELOX+Bevacizumab	3.01	3.12	3.05	2.69	2.12	2.49	1.44	0	61.08	WT	MUT
XELOX+Bevacizumab	3.21	2.80	2.80	2.50	2.05	2.39	0.39	0	78.52	WT	NA
XELOX+Bevacizumab	3.21	2.86	3.31	2.78	2.59	2.60	2.85	0	84.93	MUT	NA
XELOX+Bevacizumab	3.13	2.78	3.35	2.34	2.06	2.61	1.18	0	62.55	WT	WT
XELOX+Bevacizumab	3.11	2.32	3.06	2.16	2.23	1.72	-0.96	0	83.15	MUT	NA
XELOX+Bevacizumab	2.73	2.55	2.73	2.59	1.90	1.96	-1.45	1	64.23	WT	NA
XELOX+Bevacizumab	2.89	2.33	2.82	2.22	1.84	1.76	-2.20	0	60.16	WT	NA
XELOX+Bevacizumab	3.10	2.90	3.28	2.62	2.12	2.51	1.52	0	84.17	MUT	MUT
XELOX+Bevacizumab	3.07	2.47	3.04	2.20	1.88	2.02	-1.07	0	60.19	WT	WT
XELOX+Bevacizumab	2.91	3.04	3.32	2.27	1.81	2.52	0.55	0	79.44	WT	MUT
XELOX+Bevacizumab	2.96	2.65	2.96	2.33	2.04	2.10	-0.52	0	78.46	WT	MUT
XELOX+Bevacizumab	3.18	2.76	3.38	2.09	1.94	2.41	0.47	0	82.56	WT	MUT
XELOX+Bevacizumab	3.14	2.64	3.21	2.20	2.36	1.77	0.16	0	84.01	WT	NA
XELOX+Bevacizumab	3.19	2.75	3.14	2.83	1.69	1.79	-0.24	0	86.93	WT	NA
XELOX+Bevacizumab	3.30	2.72	3.15	2.36	2.01	2.58	0.89	0	62.72	MUT	WT
XELOX+Bevacizumab	3.24	2.87	3.06	2.14	2.14	2.34	0.62	0	60.81	WT	WT
XELOX+Bevacizumab	3.48	2.60	3.15	2.98	2.28	2.05	1.56	0	68.67	WT	WT
XELOX+Bevacizumab	2.93	3.00	3.33	2.56	2.05	2.03	0.74	0	59.14	WT	NA
XELOX+Bevacizumab	2.96	3.31	3.11	2.68	2.31	2.46	2.07	0	78.23	MUT	WT
XELOX+Bevacizumab	2.74	2.88	2.66	2.64	2.12	2.62	0.25	0	62.36	WT	NA
XELOX+Bevacizumab	2.45	2.61	2.88	2.47	1.87	1.68	-2.04	0	65.87	WT	NA
XELOX+Bevacizumab	3.09	3.03	2.85	2.64	2.00	2.05	0.33	0	60.91	WT	NA

XELOX+Bevacizumab	3.29	2.91	3.39	2.70	2.06	2.21	1.60	0	58.97	WT	MUT
XELOX+Bevacizumab	2.95	2.67	3.46	2.70	1.97	1.92	0.34	0	64.95	WT	NA
XELOX+Bevacizumab	3.26	3.04	3.36	2.60	2.38	3.14	3.31	1	27.5	MUT	WT
XELOX+Bevacizumab	3.29	2.15	3.50	2.30	2.33	2.23	0.65	0	79.87	NA	NA
XELOX+Bevacizumab	3.14	2.65	3.24	2.58	1.92	1.81	-0.13	1	52.53	MUT	NA
XELOX+Bevacizumab	3.17	2.92	3.60	2.77	1.85	2.09	1.28	0	60.52	WT	NA
XELOX+Bevacizumab	3.24	2.69	3.47	2.26	2.12	2.46	1.20	0	69.52	NA	WT
XELOX+Bevacizumab	3.31	2.77	3.27	2.86	2.25	1.95	1.46	0	79.08	MUT	WT
XELOX+Bevacizumab	3.16	2.87	3.40	2.74	1.72	1.75	0.20	0	74.94	WT	NA
XELOX+Bevacizumab	3.14	2.63	3.32	2.41	1.96	1.98	0.04	0	84.14	WT	NA
XELOX+Bevacizumab	3.02	2.66	3.10	2.19	2.29	2.51	0.62	0	82.83	WT	NA
XELOX+Bevacizumab	3.45	2.90	3.47	2.88	1.96	1.97	1.63	0	67.98	WT	MUT
XELOX+Bevacizumab	3.81	3.18	3.59	2.99	2.76	2.33	4.79	1	38.67	WT	WT
XELOX+Bevacizumab	3.29	3.22	3.15	3.06	2.11	2.03	1.99	0	60.45	WT	NA
XELOX+Bevacizumab	2.65	2.59	2.84	1.88	1.81	2.16	-2.08	0	83.38	WT	NA
XELOX+Bevacizumab	3.59	2.81	3.26	2.82	1.88	1.83	0.96	0	75.79	WT	NA
XELOX+Bevacizumab	3.04	2.77	3.19	2.42	2.27	2.04	0.61	0	60.58	WT	WT
XELOX+Bevacizumab	3.05	2.61	3.14	2.15	2.08	2.14	-0.26	0	68.7	NA	WT
XELOX+Bevacizumab	3.28	2.88	3.26	2.47	1.94	2.49	1.16	0	65.97	WT	MUT
XELOX+Bevacizumab	3.31	2.69	3.09	2.48	2.09	2.39	0.85	0	78.19	WT	MUT
XELOX+Bevacizumab	2.92	2.43	3.38	2.29	2.19	2.01	-0.13	1	8.87	WT	WT
XELOX+Bevacizumab	3.25	2.67	3.50	2.31	1.98	2.12	0.60	0	59.63	WT	MUT
XELOX+Bevacizumab	3.50	3.24	3.43	3.09	2.06	1.83	2.42	0	77.4	WT	MUT
XELOX+Bevacizumab	3.02	2.89	3.36	2.47	1.89	2.23	0.57	0	65.84	WT	NA
XELOX+Bevacizumab	2.94	2.67	3.30	2.55	2.14	2.12	0.47	0	77.7	WT	NA
XELOX+Bevacizumab	3.01	2.63	3.24	2.92	2.19	2.03	0.84	0	61.9	WT	WT
XELOX+Bevacizumab	2.43	2.47	3.09	2.53	1.88	1.59	-1.99	0	82.4	WT	MUT
XELOX+Bevacizumab	3.16	3.05	3.34	2.90	2.23	2.11	2.00	0	79.9	WT	MUT
XELOX+Bevacizumab	2.85	2.40	2.95	1.62	1.92	2.03	-2.19	0	87.89	WT	NA
XELOX+Bevacizumab	3.36	2.92	3.13	2.52	2.03	2.76	1.70	0	63.51	WT	MUT
XELOX+Bevacizumab	3.30	2.92	2.99	2.72	2.18	2.22	1.28	0	78.46	WT	MUT
XELOX+Bevacizumab	2.90	2.37	2.94	1.54	1.86	2.30	-2.05	0	78.03	WT	NA
XELOX+Bevacizumab	2.66	2.68	3.09	2.20	1.97	2.24	-0.80	0	84.96	WT	NA
XELOX+Bevacizumab	3.06	2.92	3.22	2.57	1.64	2.45	0.36	0	79.7	WT	MUT
XELOX+Bevacizumab	3.42	2.91	2.91	2.69	1.96	2.55	1.24	0	58.22	WT	MUT
XELOX+Bevacizumab	3.44	3.05	3.40	2.94	1.99	2.29	2.27	0	73	WT	MUT
XELOX+Bevacizumab	2.45	2.43	2.74	2.36	1.86	1.36	-3.05	0	82.56	WT	MUT
XELOX+Bevacizumab	3.34	2.67	3.21	2.26	2.40	2.38	1.36	1	31.84	WT	MUT
XELOX+Bevacizumab	2.47	2.32	2.98	2.63	1.90	1.70	-2.02	0	85.78	WT	NA
XELOX+Bevacizumab	3.17	2.98	3.38	2.92	2.07	2.06	1.61	0	66.53	WT	WT
XELOX+Bevacizumab	2.82	2.44	2.96	2.21	1.72	1.53	-2.45	0	83.55	WT	MUT
XELOX+Bevacizumab	3.31	2.99	3.40	2.71	1.80	1.94	0.93	0	73.66	WT	MUT
XELOX+Bevacizumab	3.01	2.59	3.19	2.51	1.92	1.98	-0.35	0	63.05	WT	NA
XELOX+Bevacizumab	3.15	3.19	3.24	2.61	2.31	1.88	1.57	0	78.69	WT	WT
XELOX+Bevacizumab	2.83	2.58	3.34	2.53	1.78	1.95	-0.66	0	71.92	WT	WT
XELOX+Bevacizumab	3.13	2.87	3.34	2.63	2.18	2.08	1.24	0	78.92	WT	MUT

XELOX+Bevacizumab	3.51	2.92	3.36	2.52	2.49	2.02	2.22	0	76.19	MUT	NA
XELOX+Bevacizumab	3.22	2.90	3.34	2.52	1.73	2.26	0.61	0	59.86	WT	MUT
XELOX+Bevacizumab	2.77	2.88	3.29	2.45	1.78	1.84	-0.59	0	60.78	WT	WT
XELOX+Bevacizumab	2.97	2.67	3.32	2.54	2.03	2.16	0.37	0	81.87	WT	NA
XELOX+Bevacizumab	2.91	3.04	3.32	2.27	1.81	2.52	0.55	0	78.85	MUT	NA
XELOX+Bevacizumab	3.02	2.76	3.22	2.48	1.91	1.61	-0.53	0	85.42	WT	NA
XELOX+Bevacizumab	2.81	2.91	3.13	2.61	1.94	2.61	0.72	0	80.59	WT	WT
XELOX+Bevacizumab	2.94	2.83	3.34	3.05	2.16	1.73	0.94	1	83.15	WT	MUT
XELOX+Bevacizumab	3.10	2.30	3.31	2.57	2.21	2.24	0.43	0	64.23	MUT	NA
XELOX+Bevacizumab	2.99	2.90	3.45	2.88	1.90	1.85	0.71	0	83.71	WT	WT
XELOX+Bevacizumab	3.31	2.89	3.21	2.73	1.88	2.63	1.52	0	67.81	WT	MUT
XELOX+Bevacizumab	3.24	2.91	3.05	2.73	1.92	2.00	0.53	0	82.99	WT	MUT

Supplementary Table 13: Individual MAPK gene expression, MPAS and overall survival of patients with CRC in the adjuvant setting (AVANT).
